# Supplementary material for: Design, Synthesis and Biological Evaluation of Quinazolinamine Derivatives as Breast Cancer Resistance Protein and P-Glycoprotein Inhibitors with Improved Metabolic Stability
Source: Biomolecules. 2023 Jan 30;13(2):253. doi: 10.3390/biom13020253 (PMC9953095; doi:10.3390/biom13020253)
Supplement: Supplementary file 1 [file biomolecules-13-00253-s001.zip › biomolecules-2059973-supplementary.pdf]

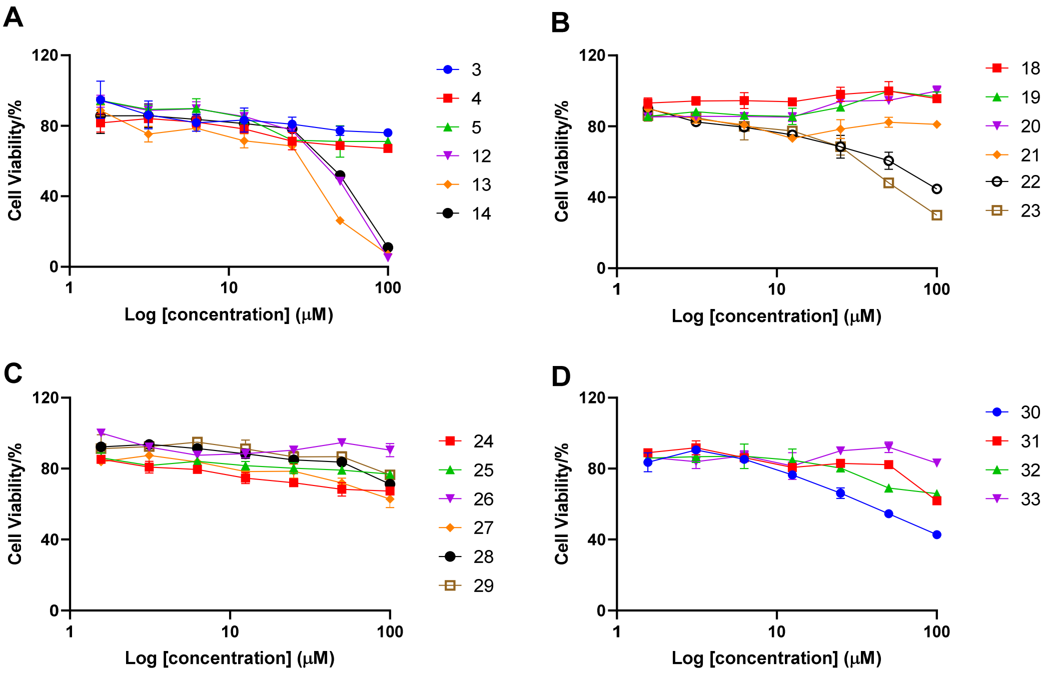

Figure S1. The cytotoxicities of quinazolinamine derivatives **3-5**, **12-14**, and **18-33** on H460 cell lines.

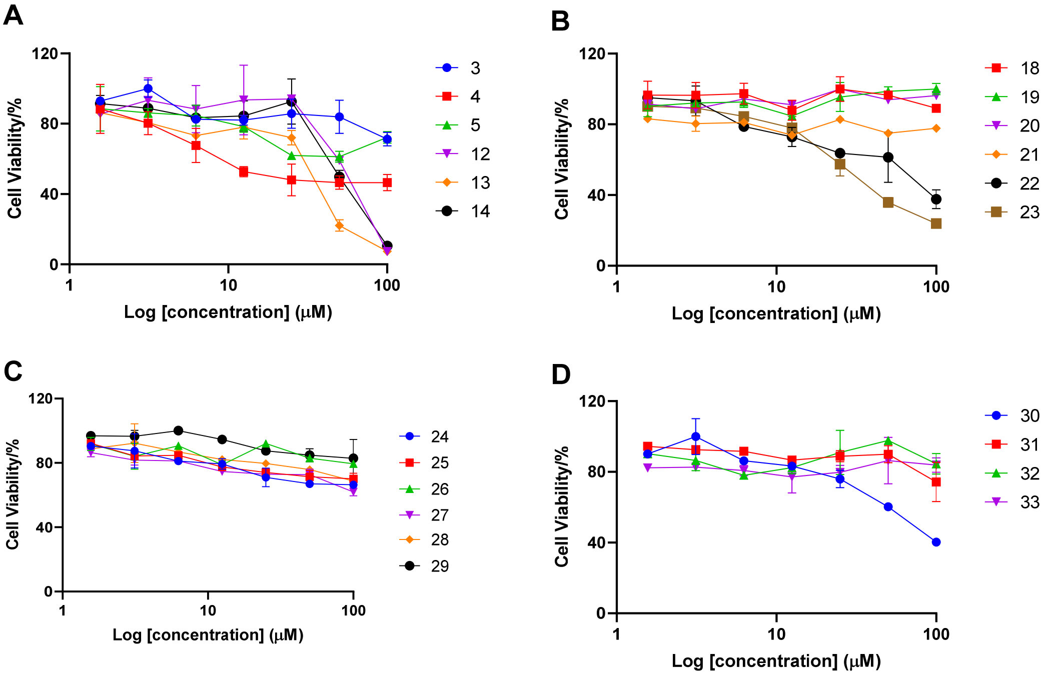

Figure S2. The cytotoxicities of quinazolinamine derivatives **3-5**, **12-14**, and **18-33** on H460/MX20 cell lines.

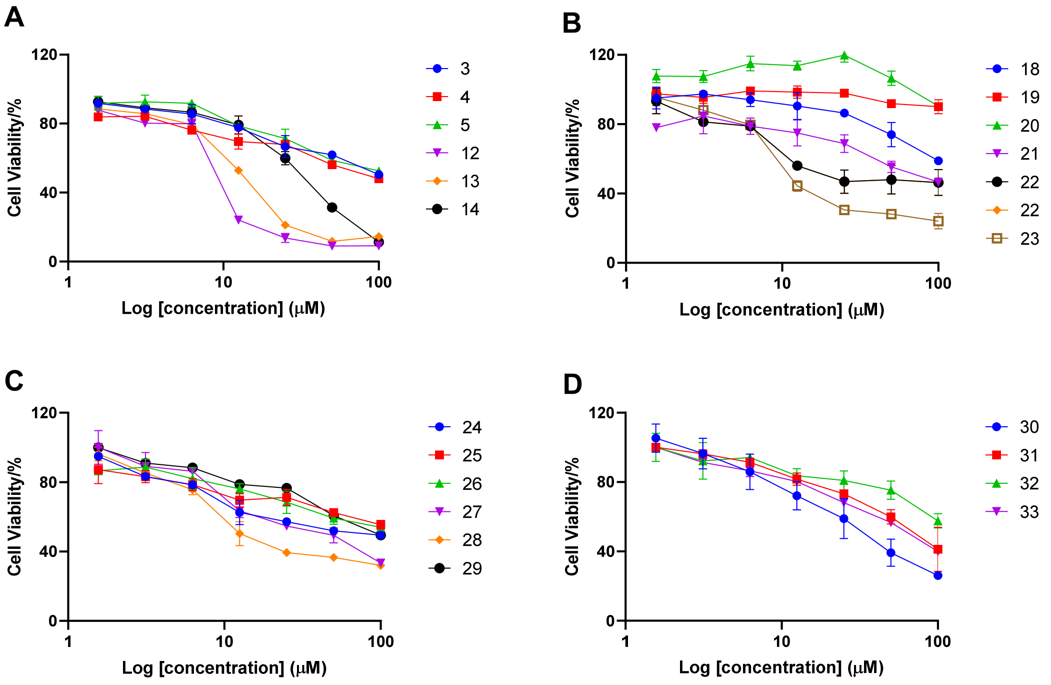

Figure S3. The cytotoxicities of quinazolinamine derivatives **3-5**, **12-14**, and **18-33** on KB-3-1 cell lines.

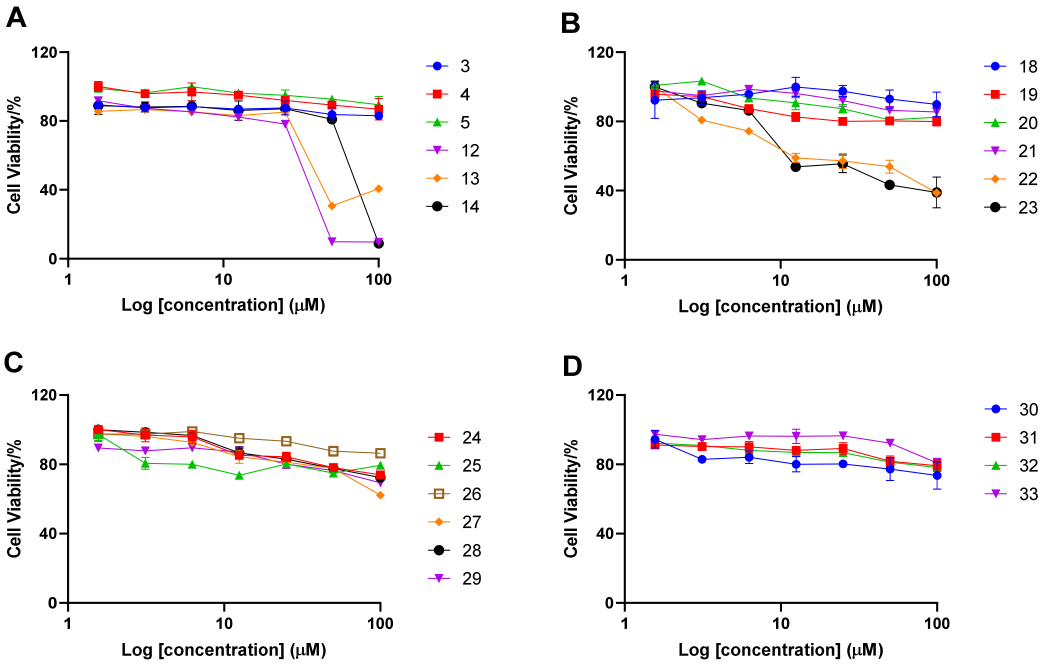

Figure S4. The cytotoxicities of quinazolinamine derivatives **3-5**, **12-14**, and **18-33** on KB-C2 cell lines.

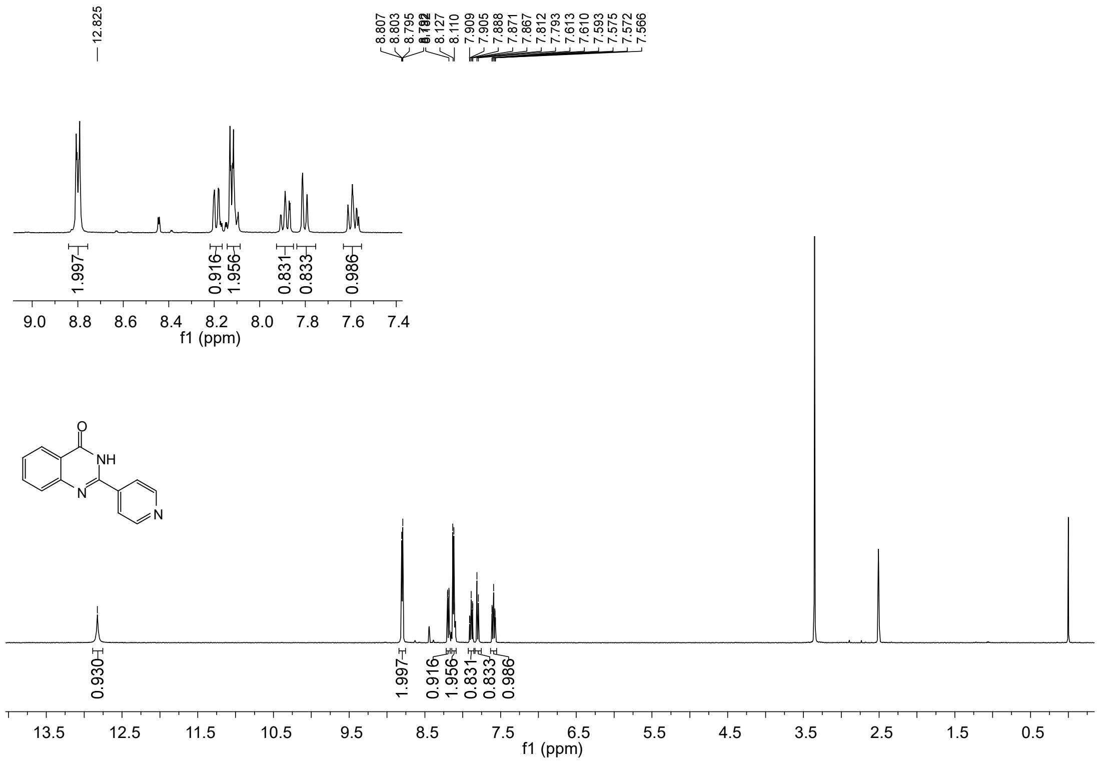

Figure S5. ^{1}H NMR (400 MHz, d_6-DMSO) of **1**

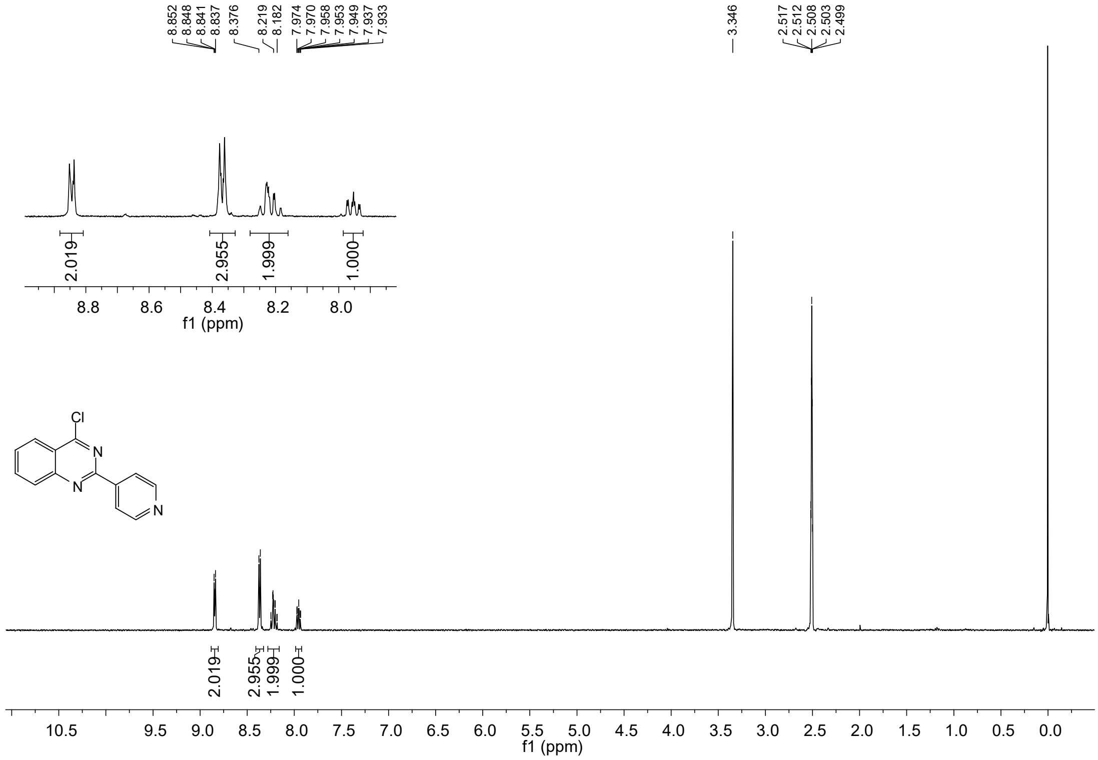

Figure S6. ^{1}H NMR (400 MHz, d_6-DMSO) of **2**

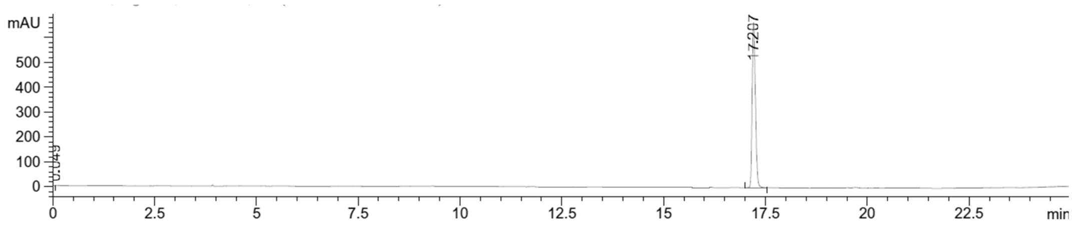

Figure S7. HPLC analysis of **3**

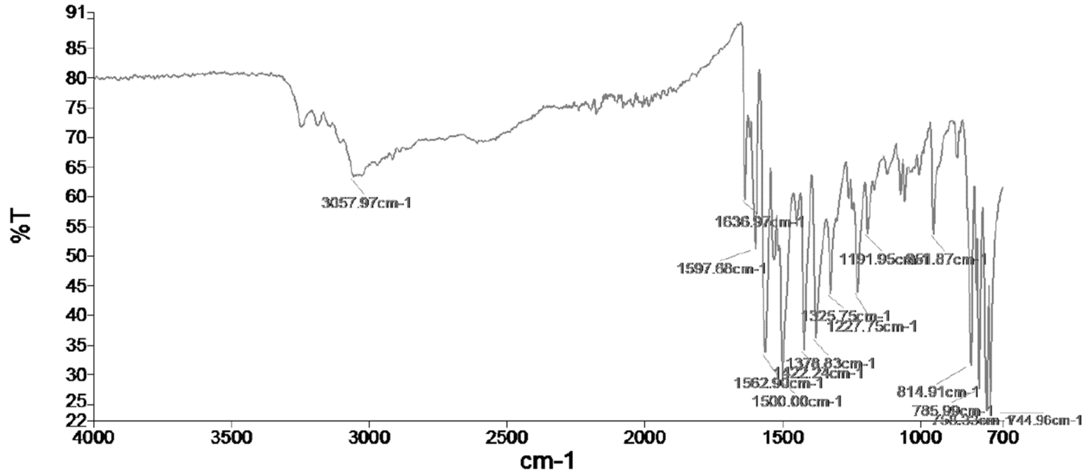

Figure S8. IR spectra of **3**

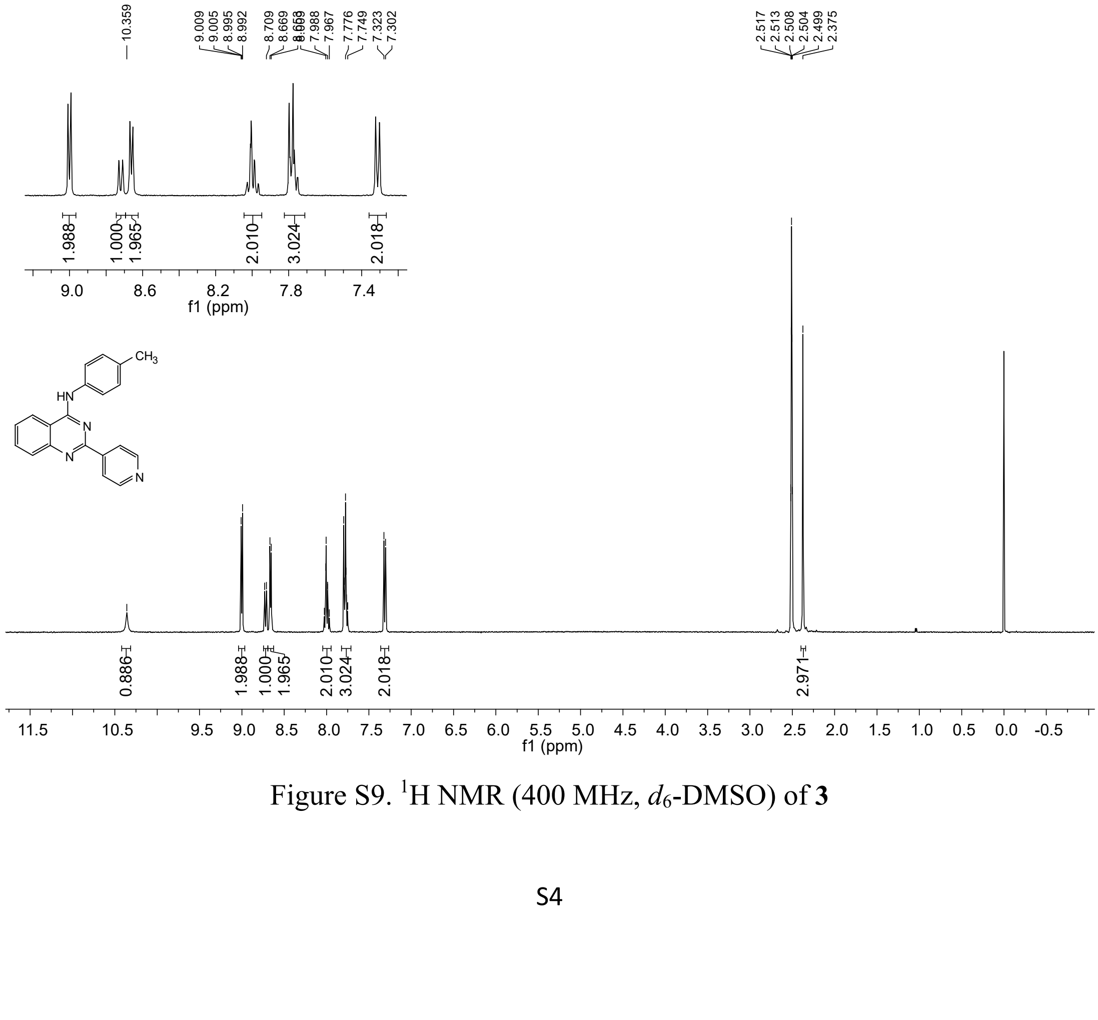

Figure S9. ^{1}H NMR (400 MHz, d_6-DMSO) of **3**

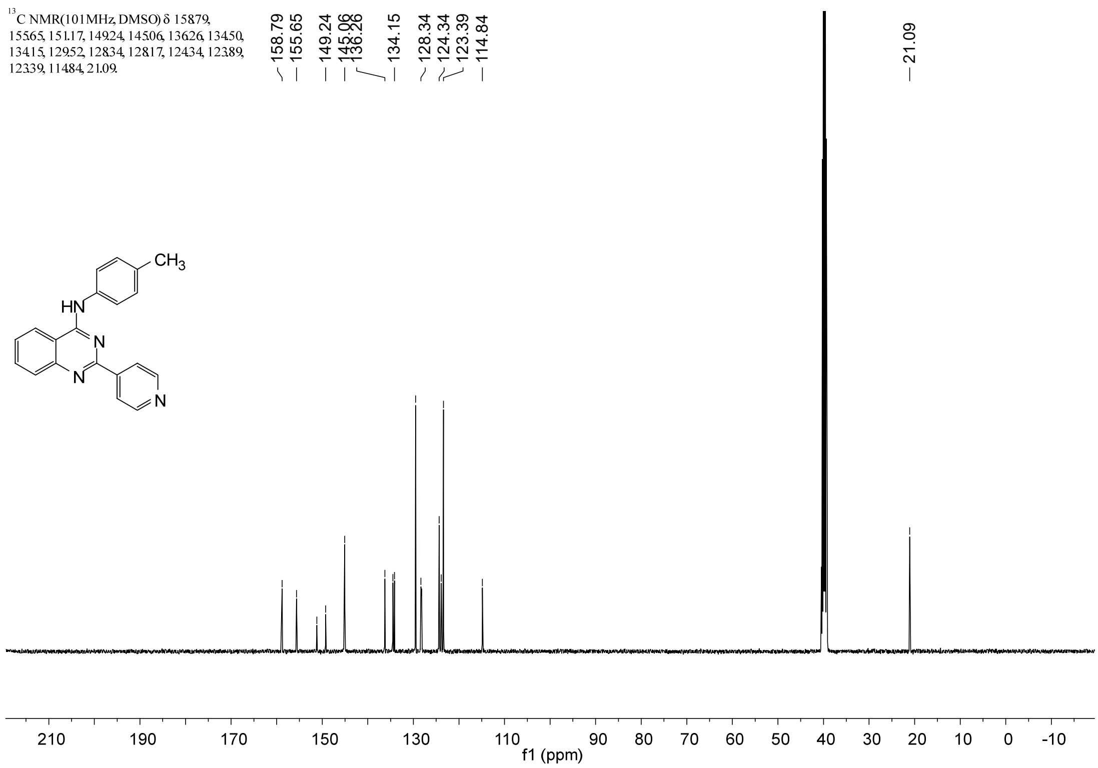

Figure S10. ^{13}C NMR (101 MHz, d_6-DMSO) of **3**

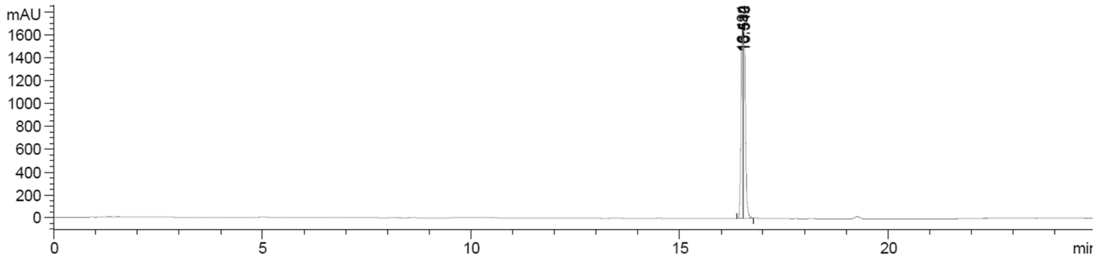

Figure S11. HPLC analysis of **4**

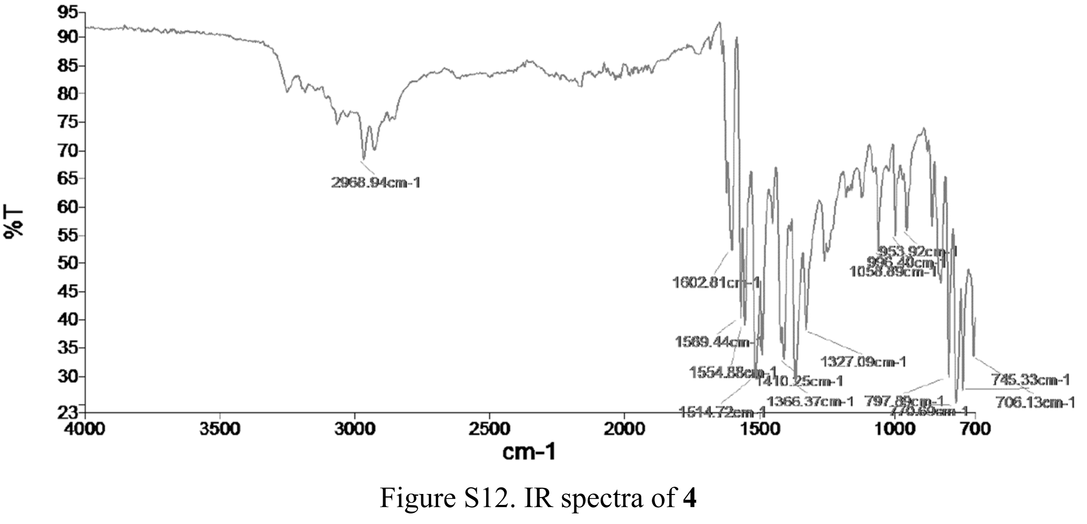

Figure S12. IR spectra of **4**

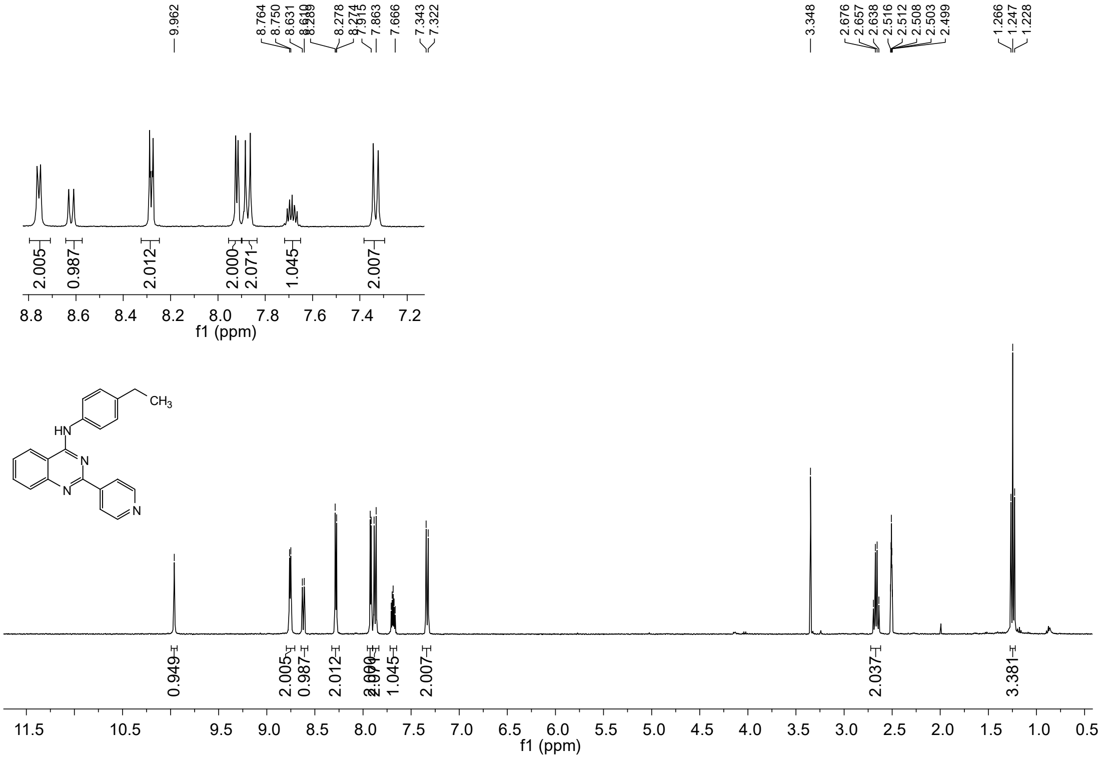

Figure S13. ^{1}H NMR (400 MHz, d_6-DMSO) of **4**

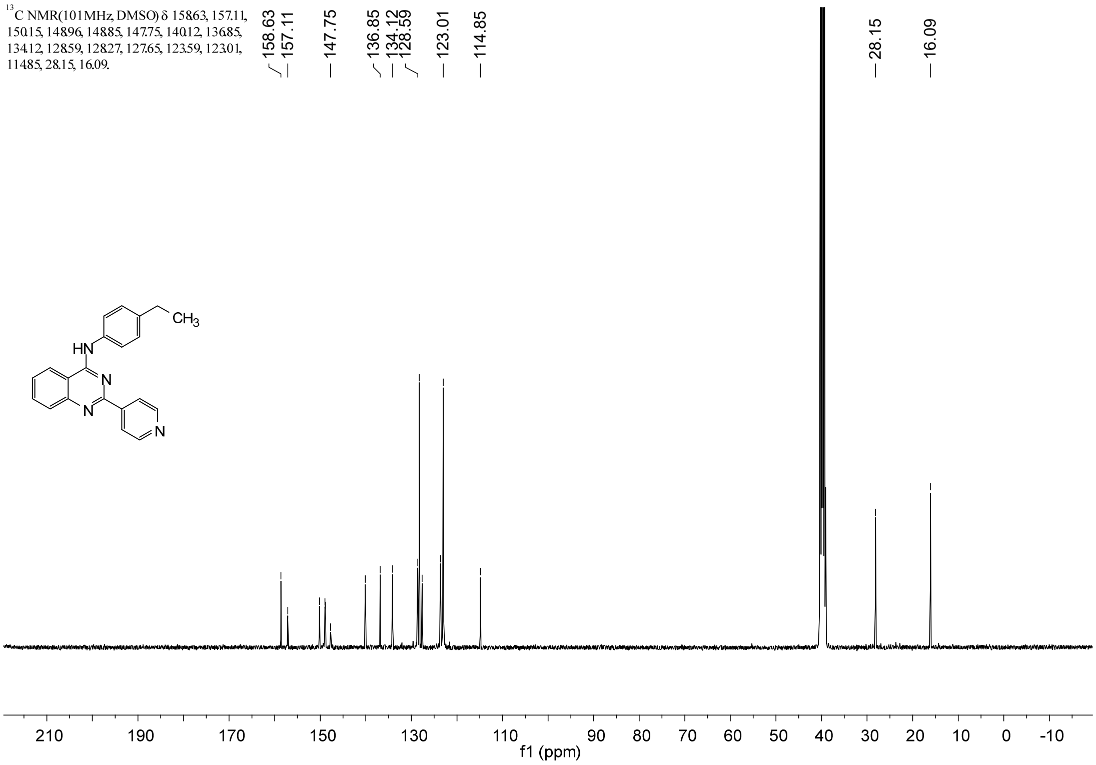

Figure S14. ^{13}C NMR (101 MHz, d_6-DMSO) of **4**

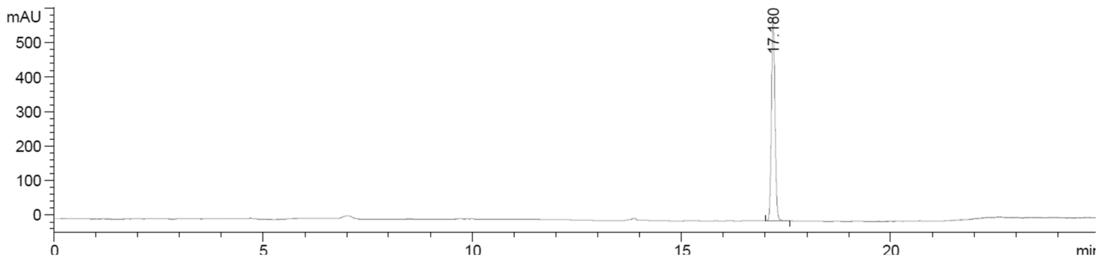

Figure S15. HPLC analysis of **5**

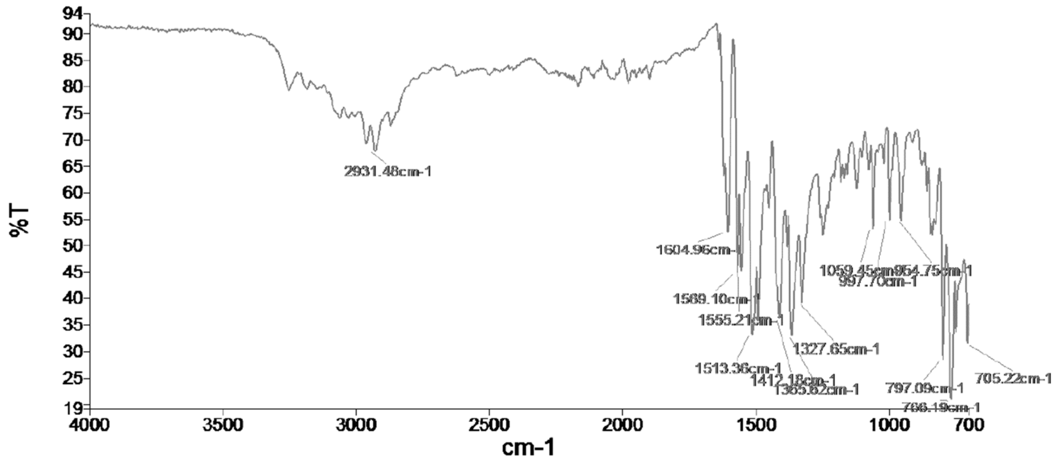

Figure S16. IR spectra of **5**

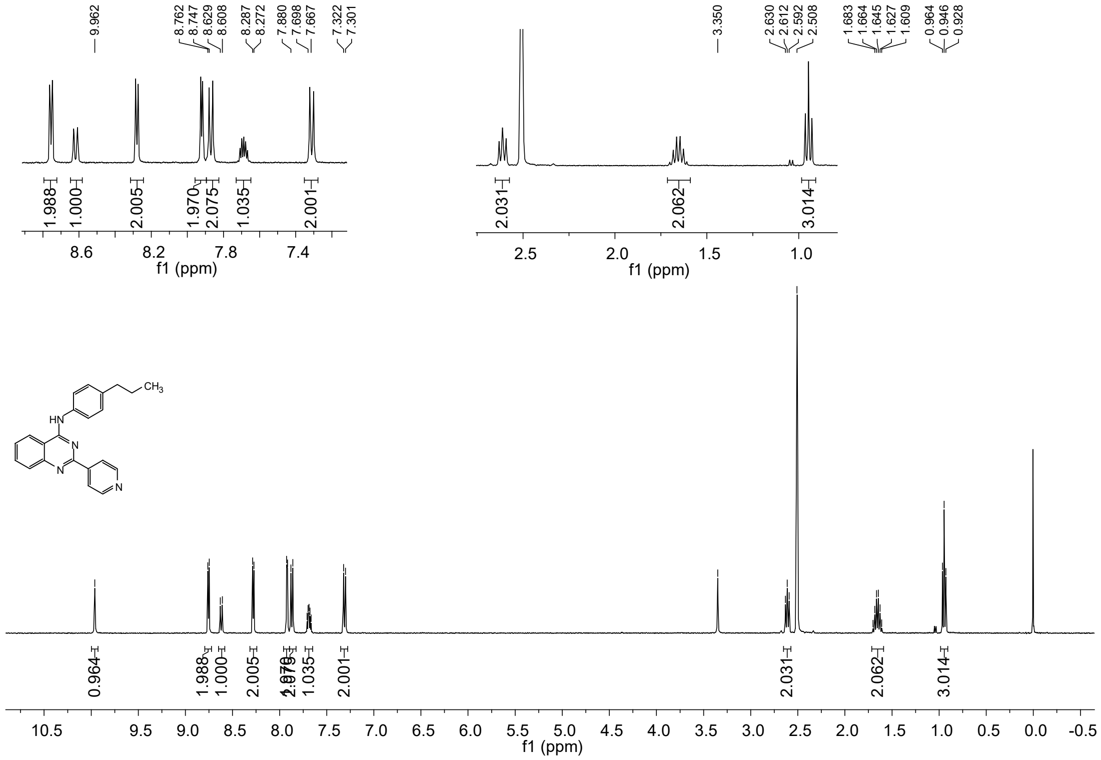

Figure S17. ^{1}H NMR (400 MHz, d_6-DMSO) of **5**

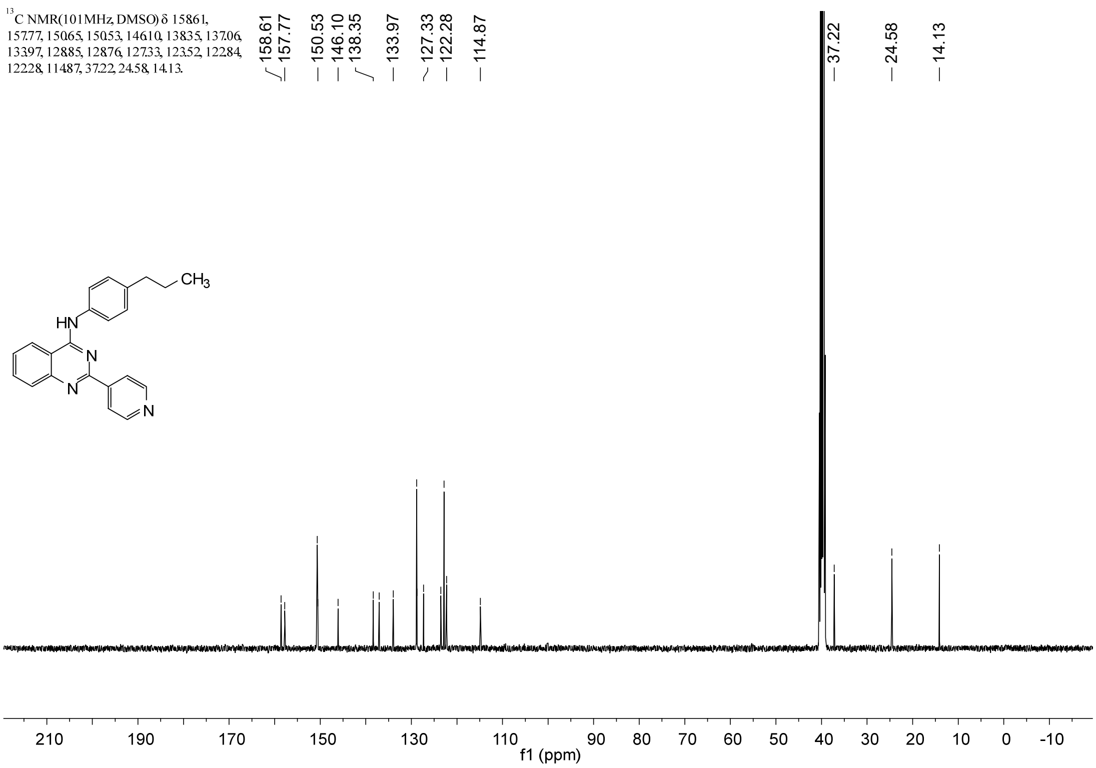

Figure S18. ^{13}C NMR (101 MHz, d_6-DMSO) of **5**

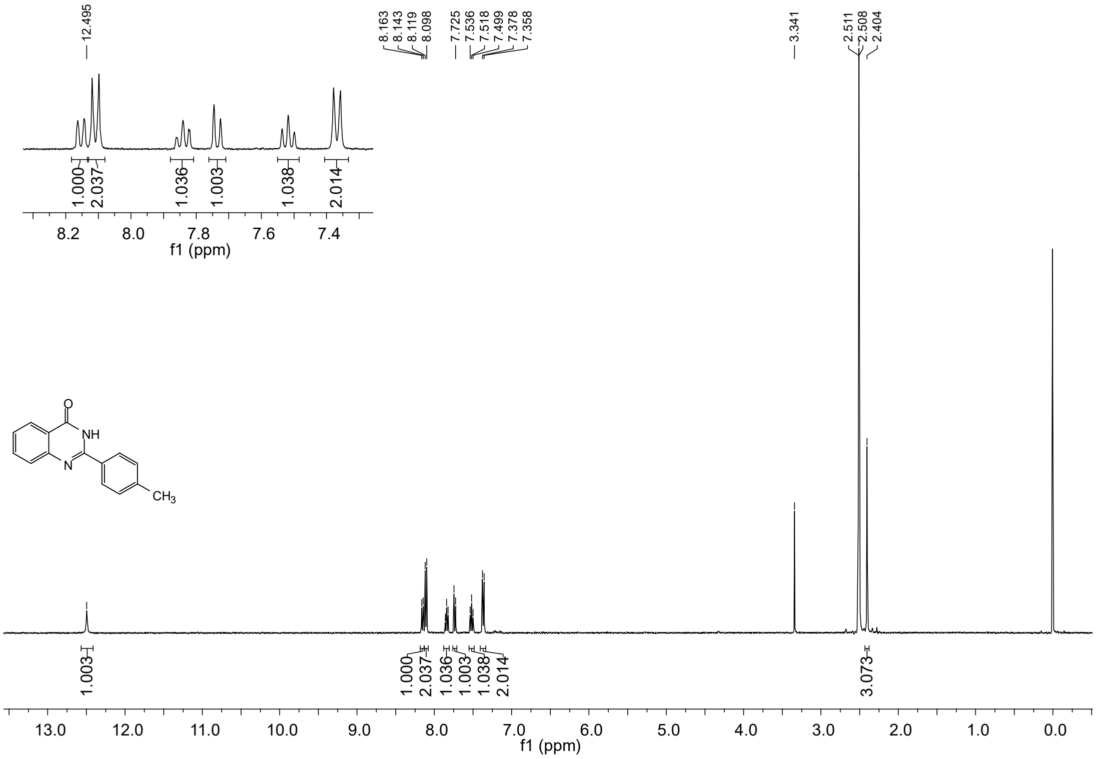

Figure S19. ^{1}H NMR (400 MHz, d_6-DMSO) of **6**

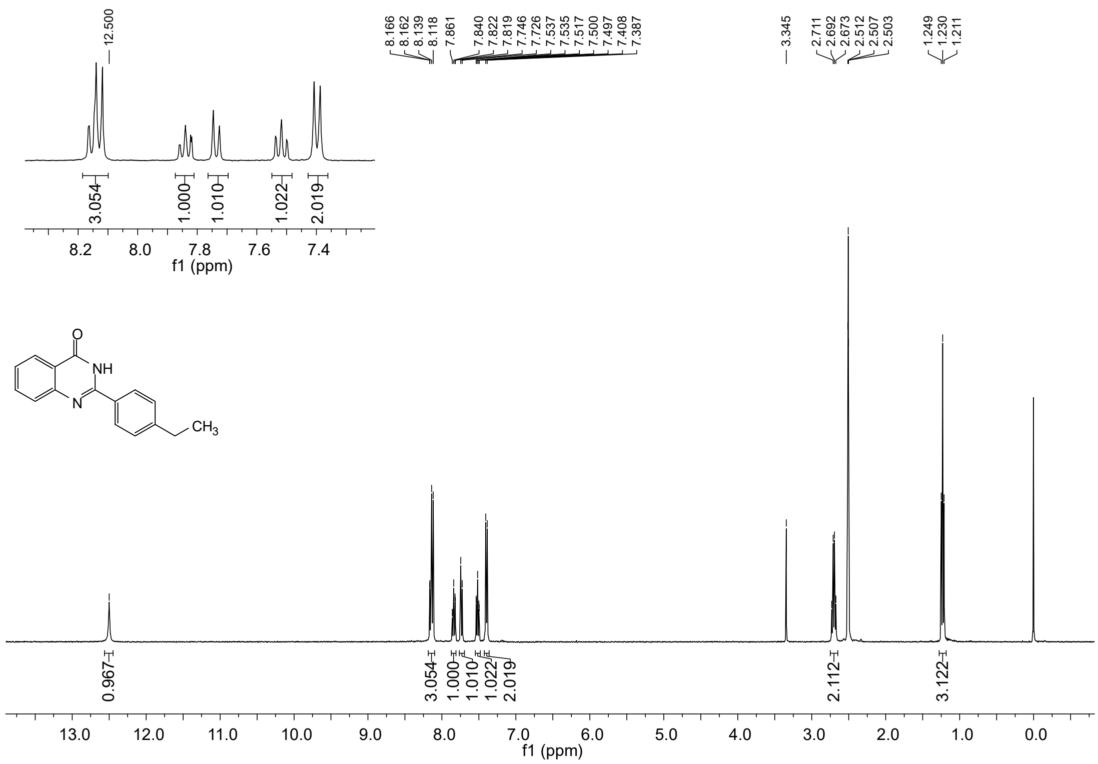

Figure S20. ^{1}H NMR (400 MHz, d_6-DMSO) of **7**

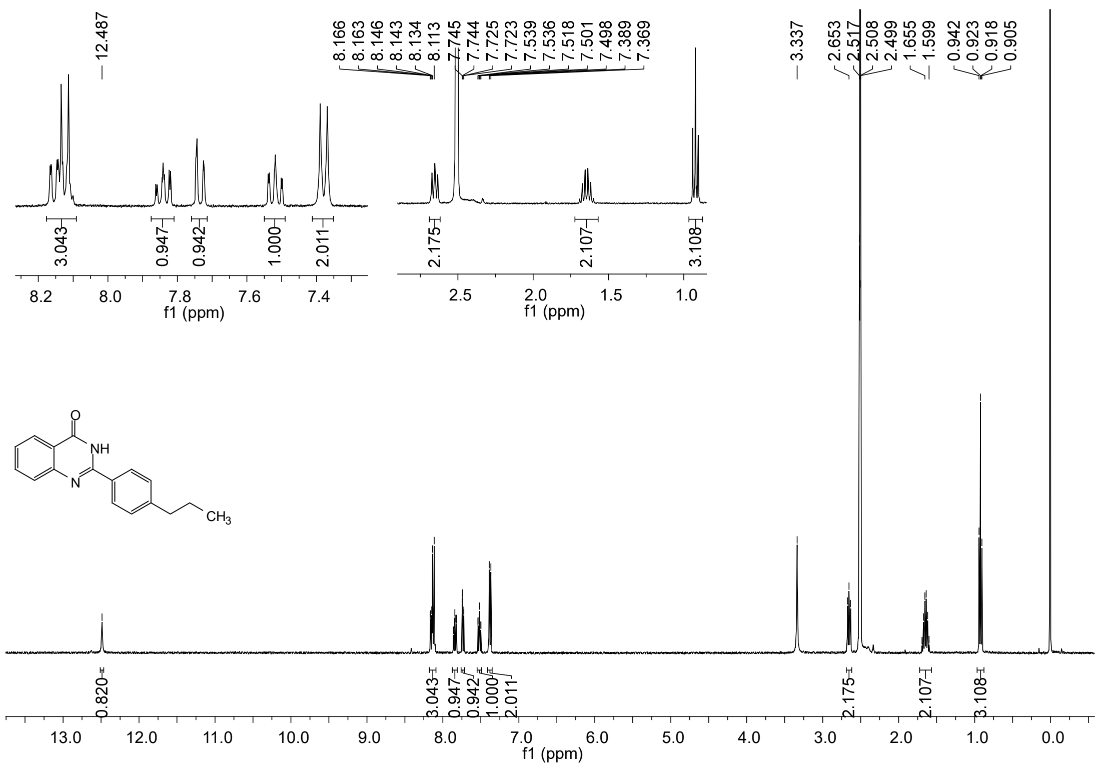

Figure S21. ^{1}H NMR (400 MHz, d_6-DMSO) of **8**

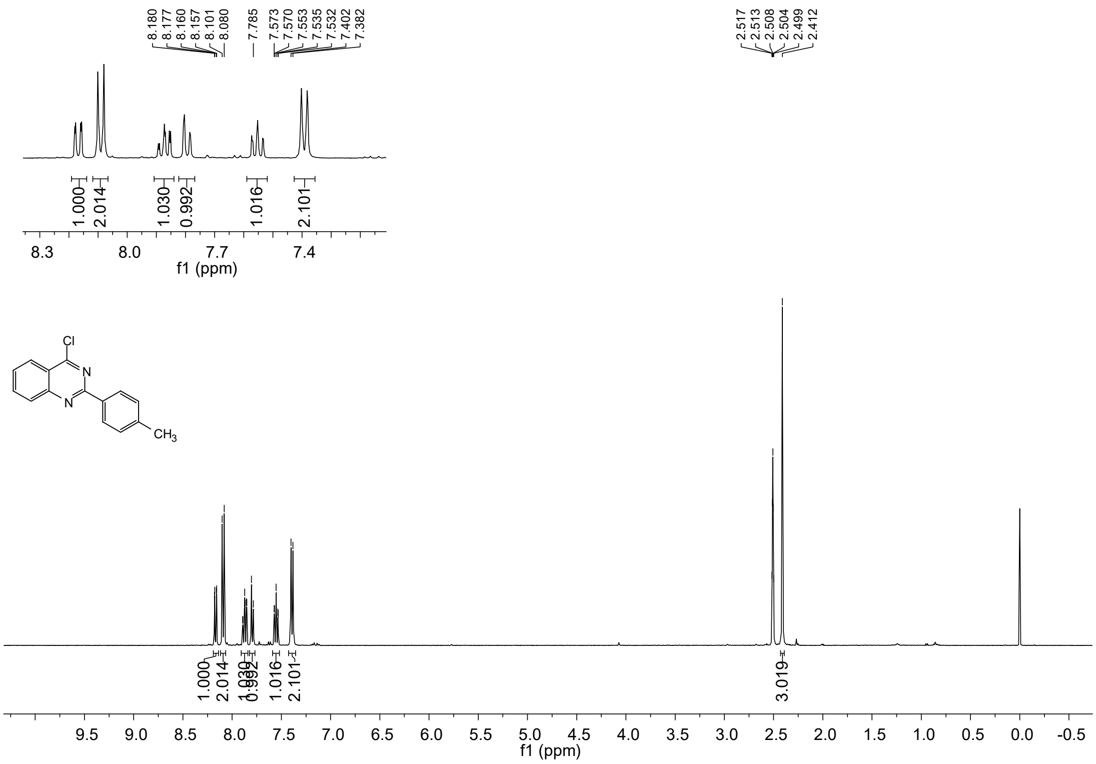

Figure S22. ^{1}H NMR (400 MHz, d_6-DMSO) of **9**

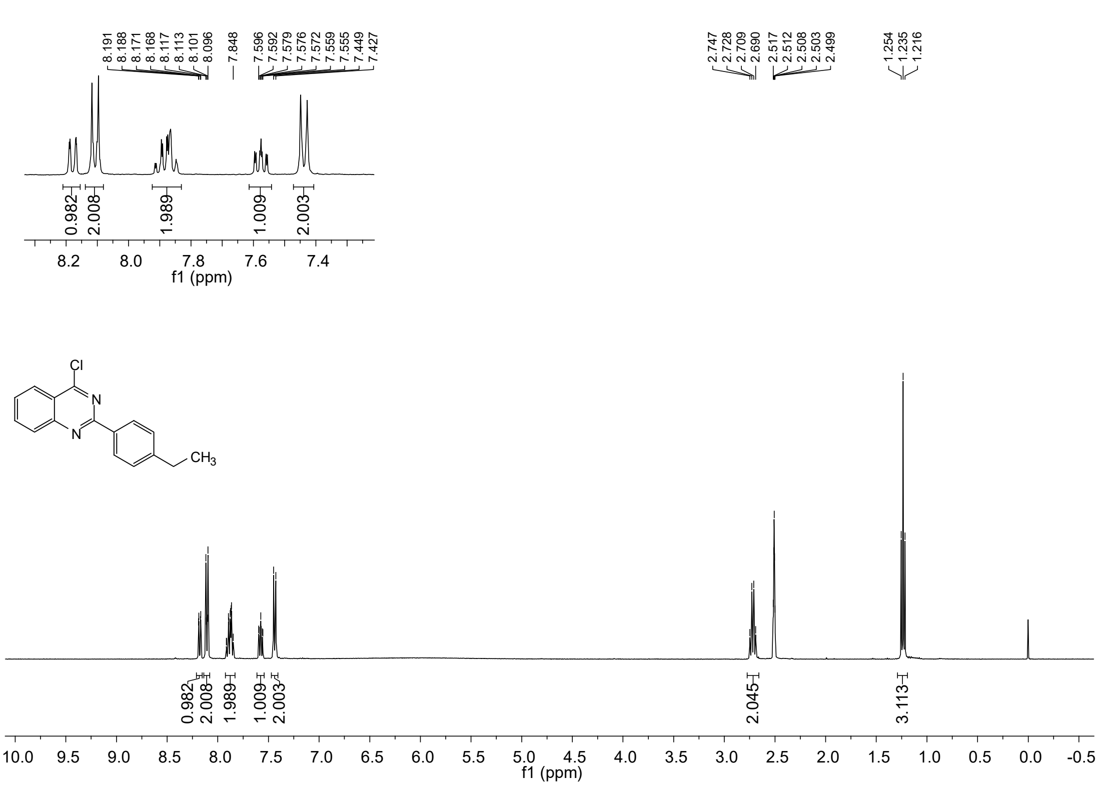

Figure S23. ^{1}H NMR (400 MHz, d_6-DMSO) of **10**

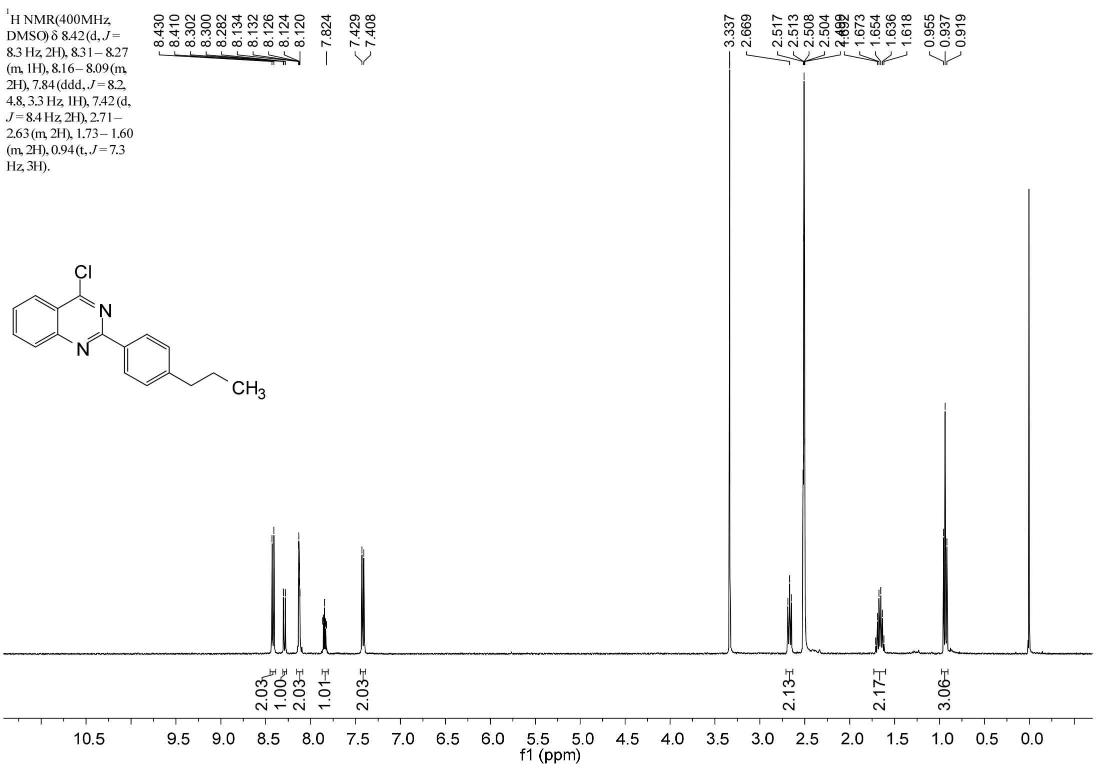

Figure S24. ^{1}H NMR (400 MHz, d_6-DMSO) of **11**

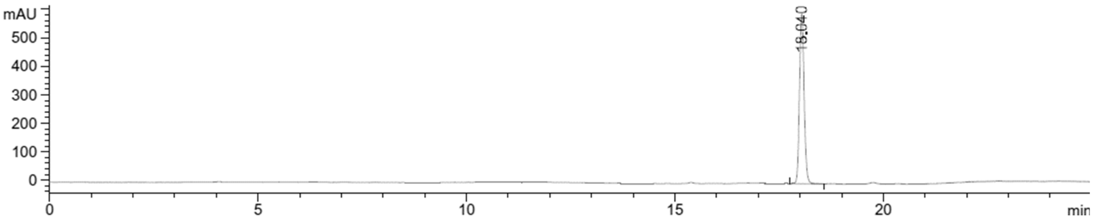

Figure S25. HPLC analysis of **12**

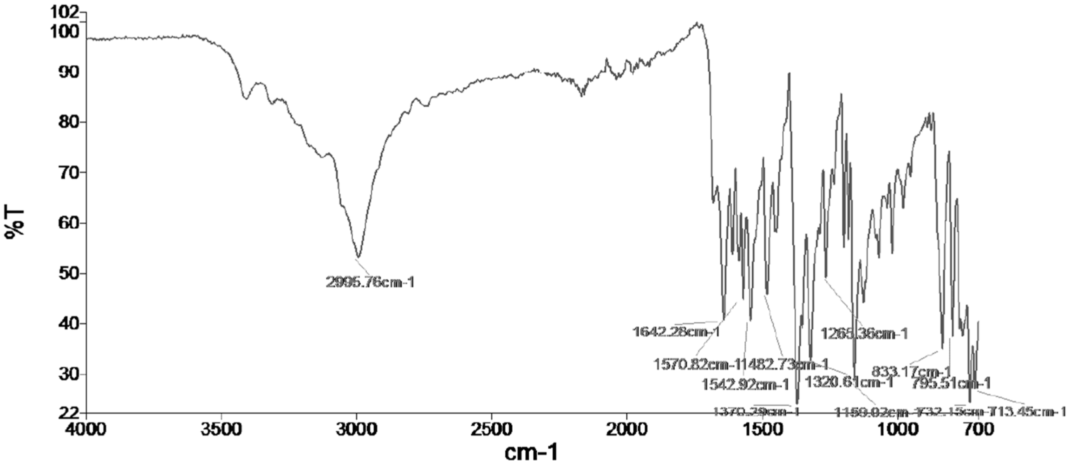

Figure S26. IR spectra of **12**

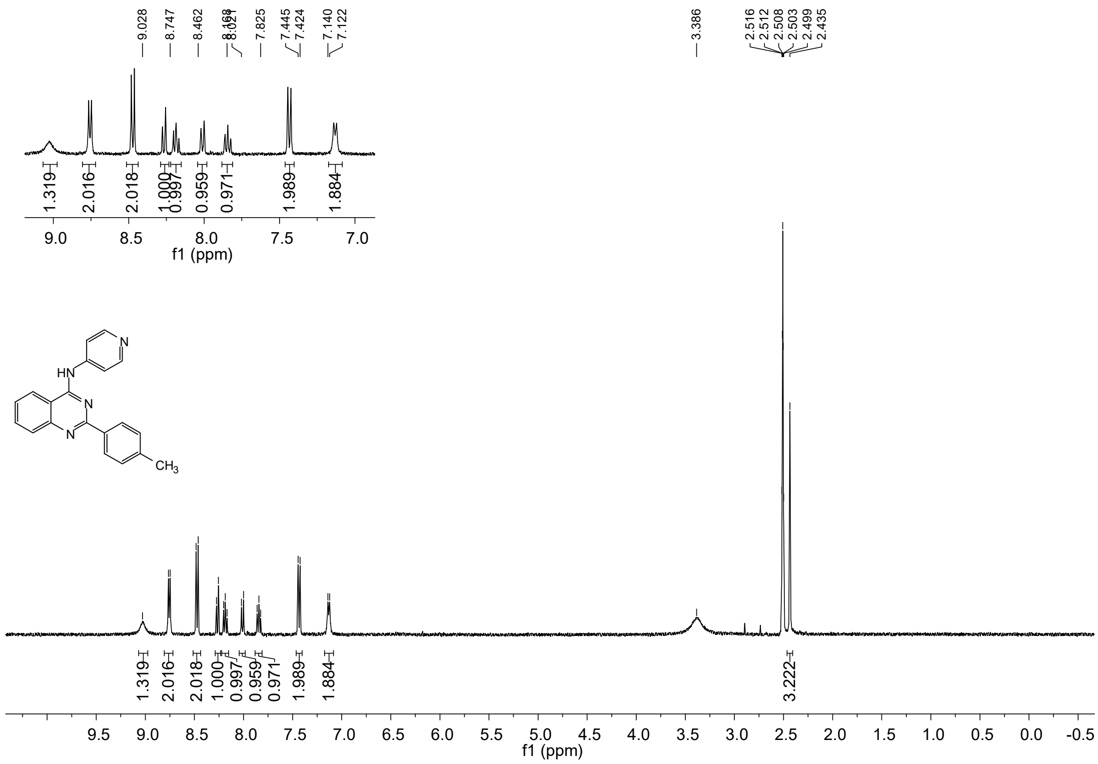

Figure S27. ^{1}H NMR (400 MHz, d_6-DMSO) of **12**

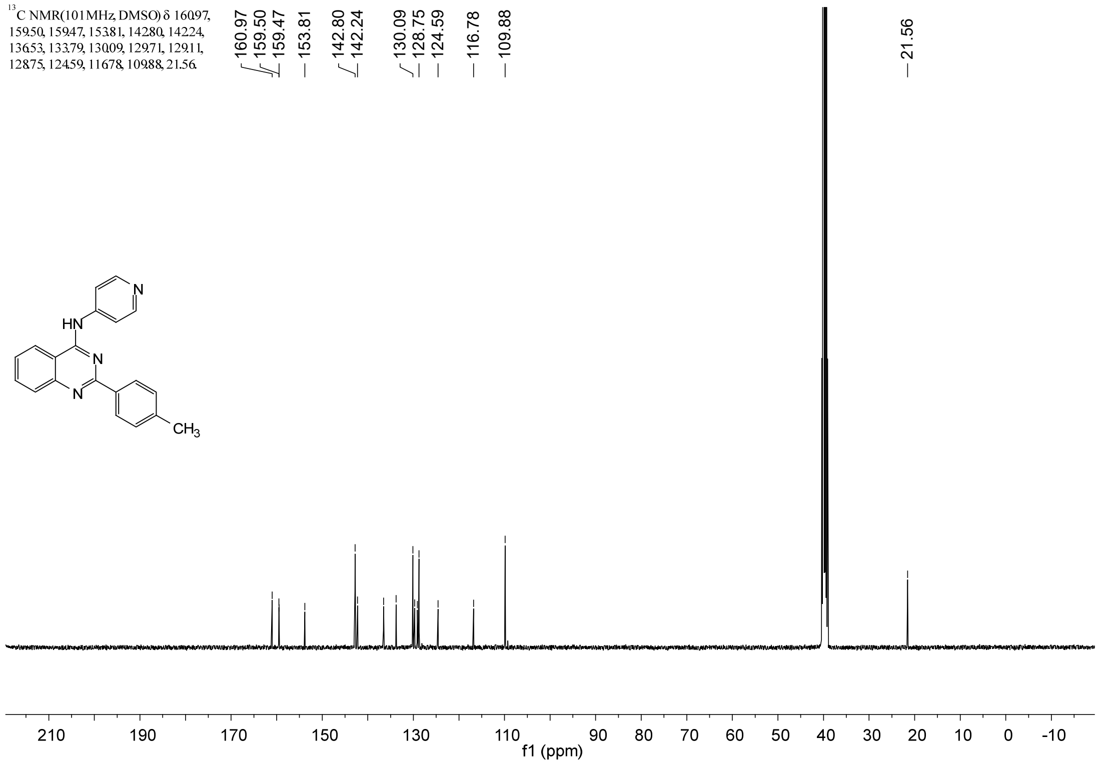

Figure S28. ^{13}C NMR (101 MHz, d_6-DMSO) of **12**

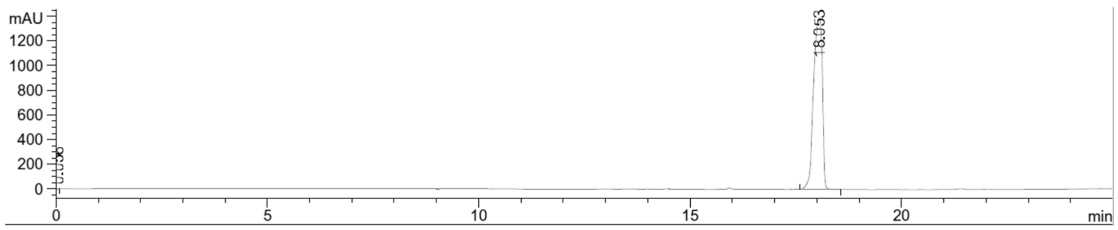

Figure S29. HPLC analysis of **13**

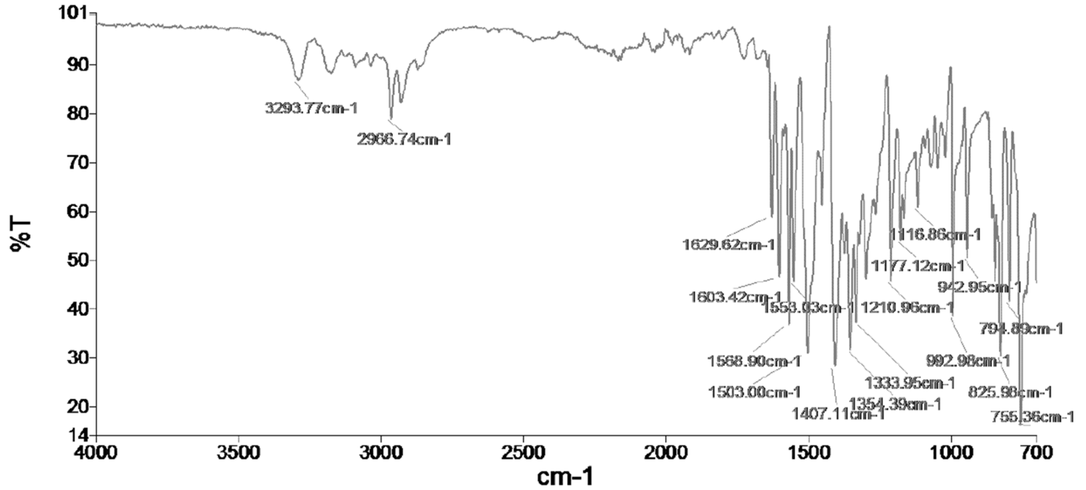

Figure S30. IR spectra of **13**

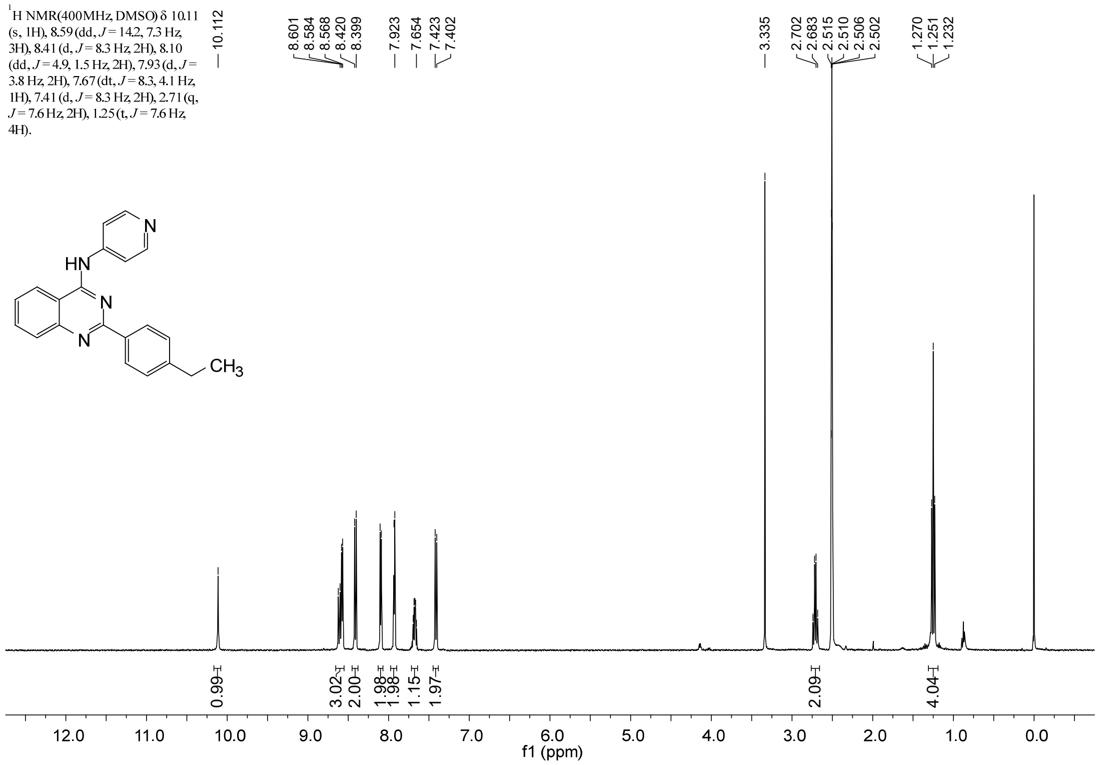

Figure S31. ^{1}H NMR (400 MHz, d_6-DMSO) of **13**

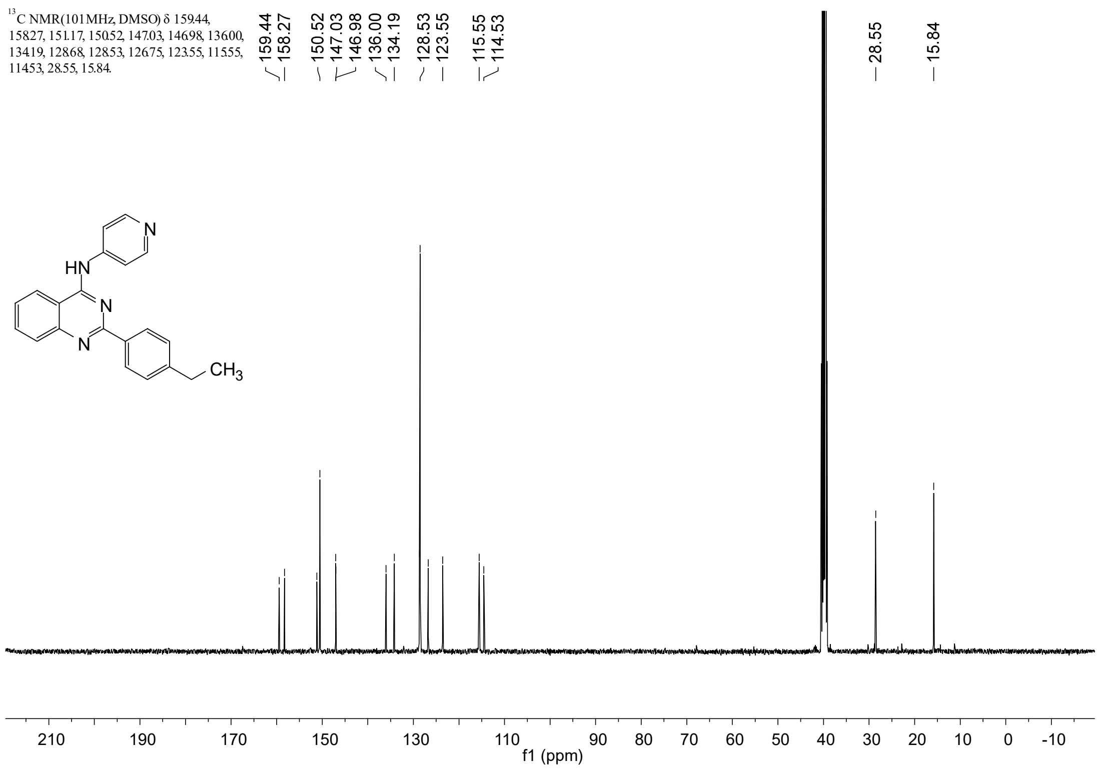

Figure S32. ^{13}C NMR (101 MHz, d_6-DMSO) of **13**

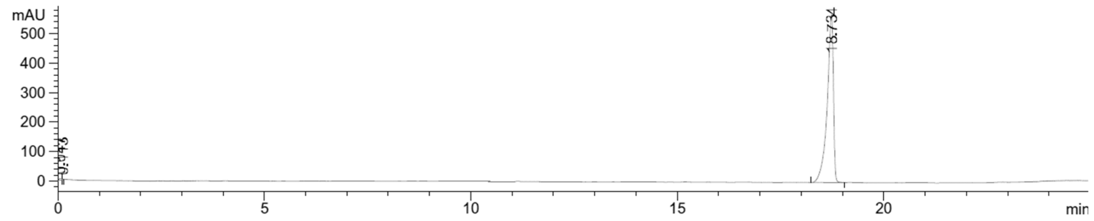

Figure S33. HPLC analysis of **14**

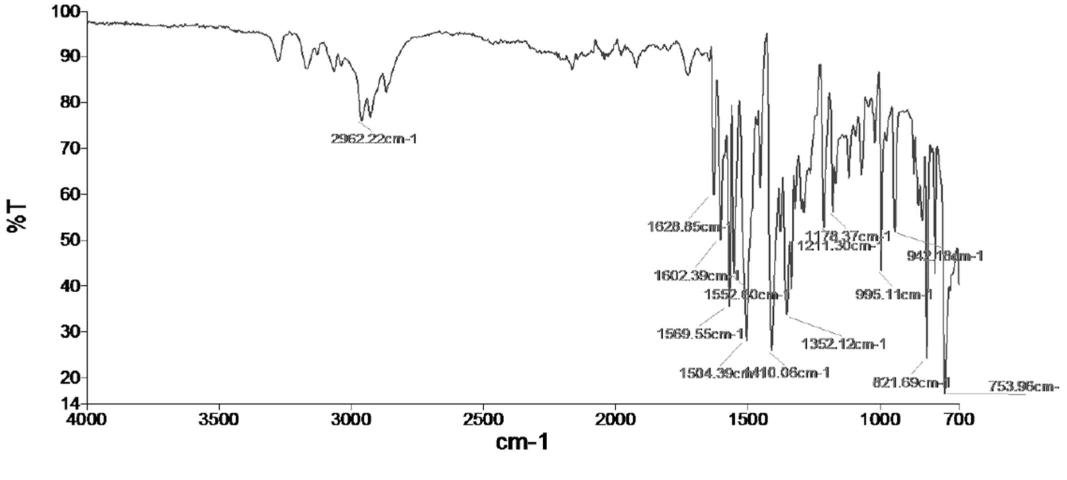

Figure S34. IR spectra of **14**

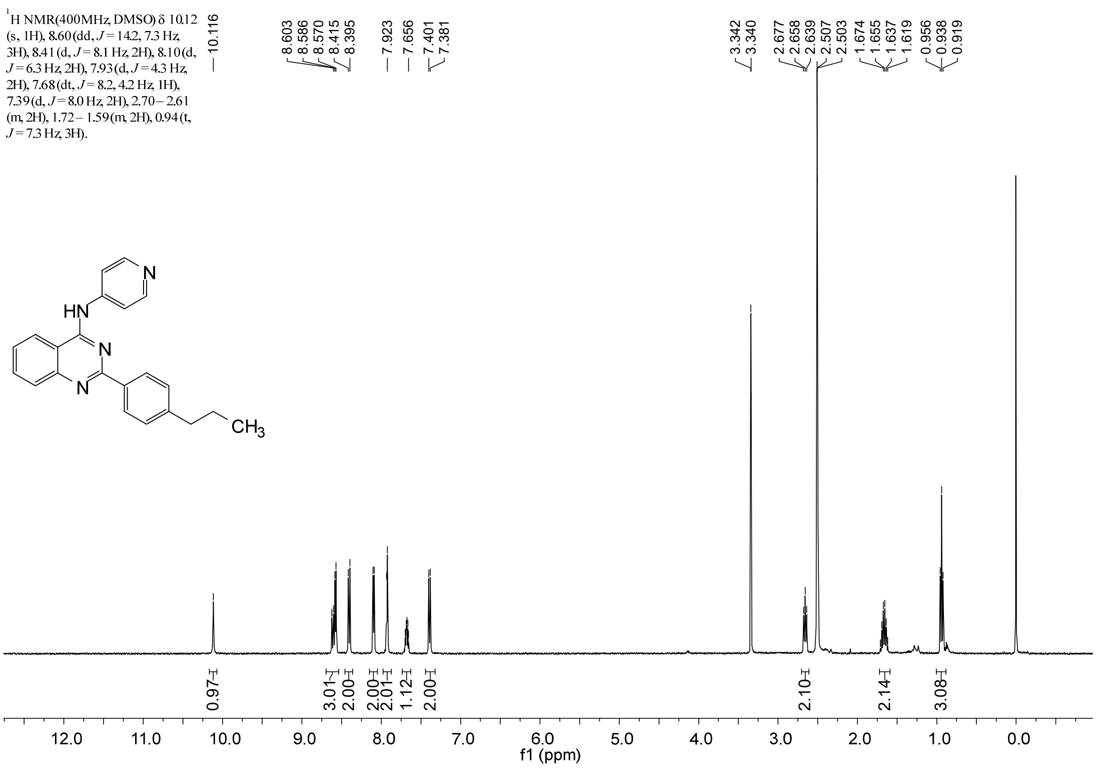

Figure S35. ^{1}H NMR (400 MHz, d_6-DMSO) of **14**

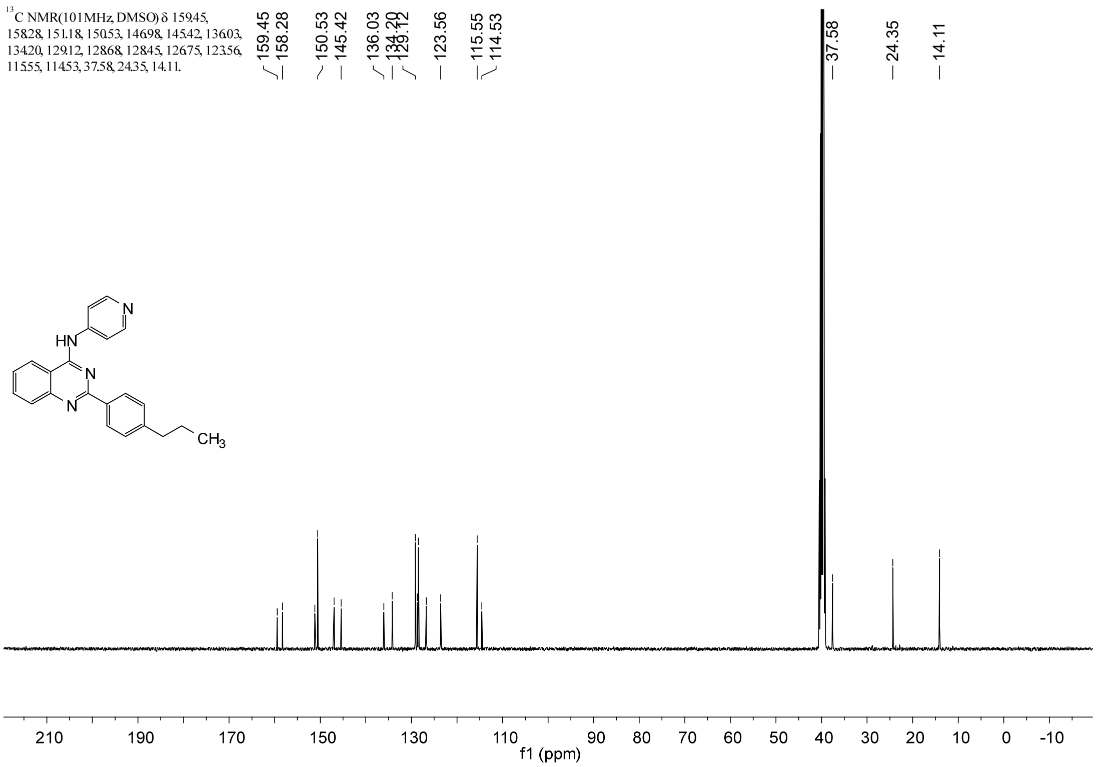

Figure S36. ^{13}C NMR (101 MHz, d_6-DMSO) of **14**

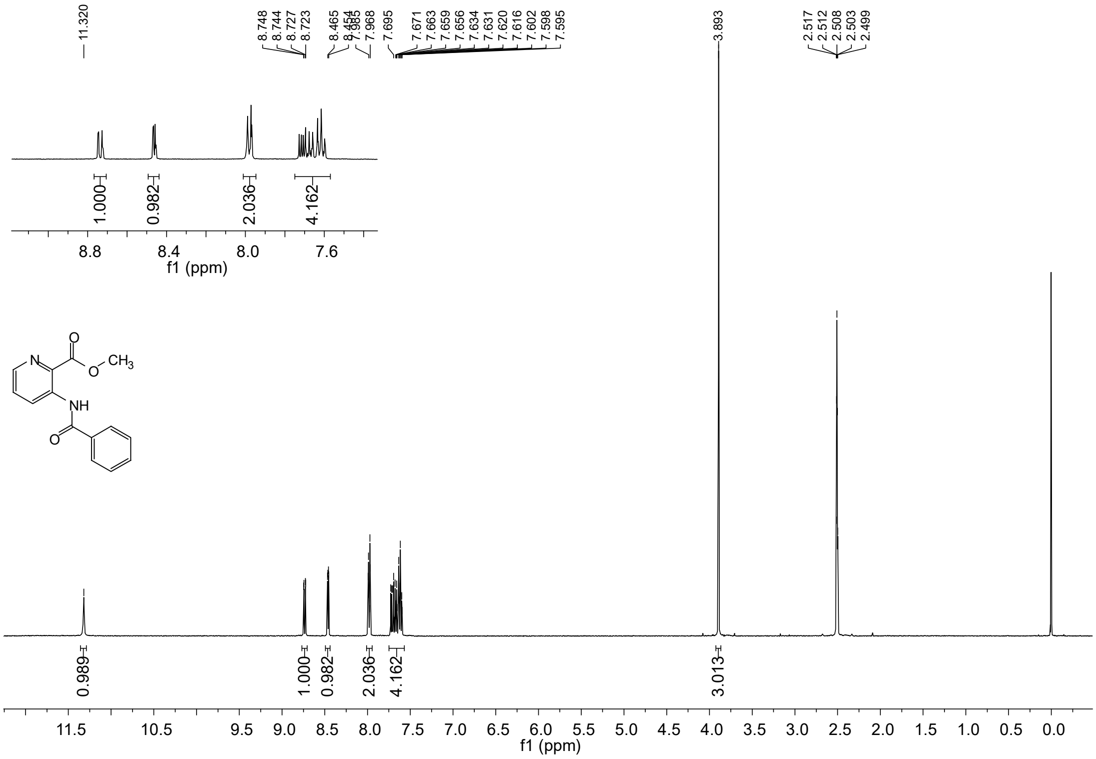

Figure S37. ^{1}H NMR (400 MHz, d_6-DMSO) of **15**

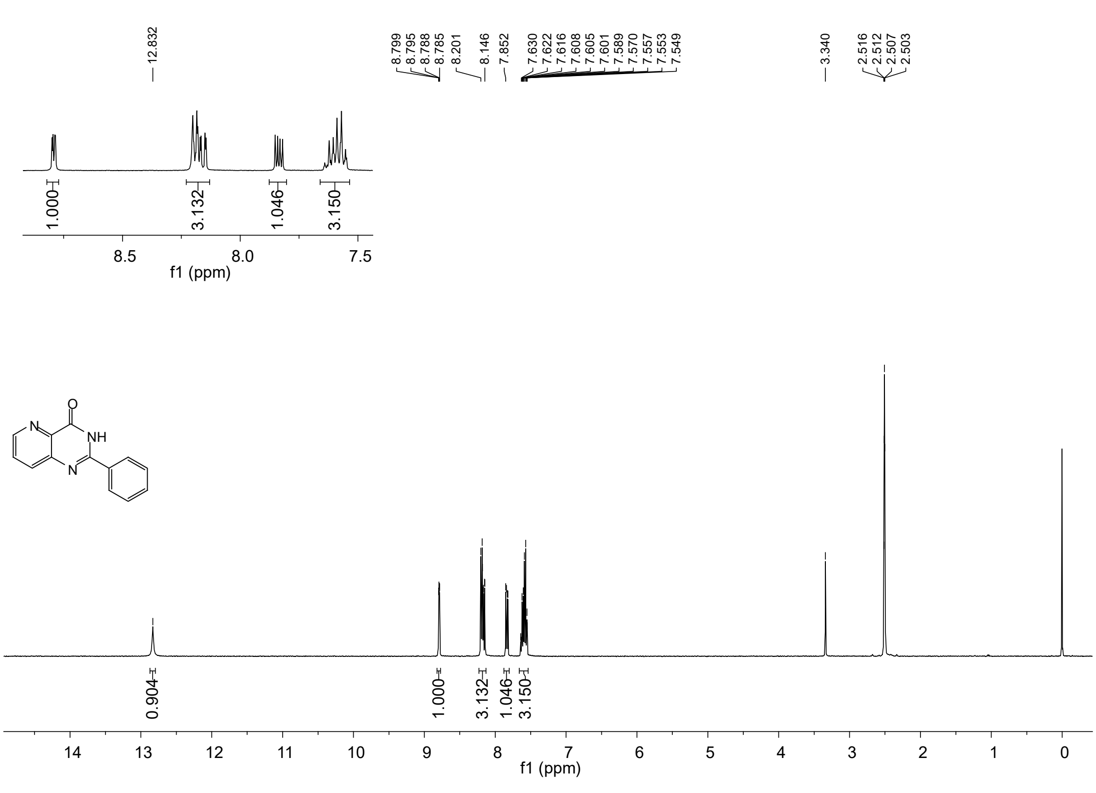

Figure S38. ^{1}H NMR (400 MHz, d_6-DMSO) of **16**

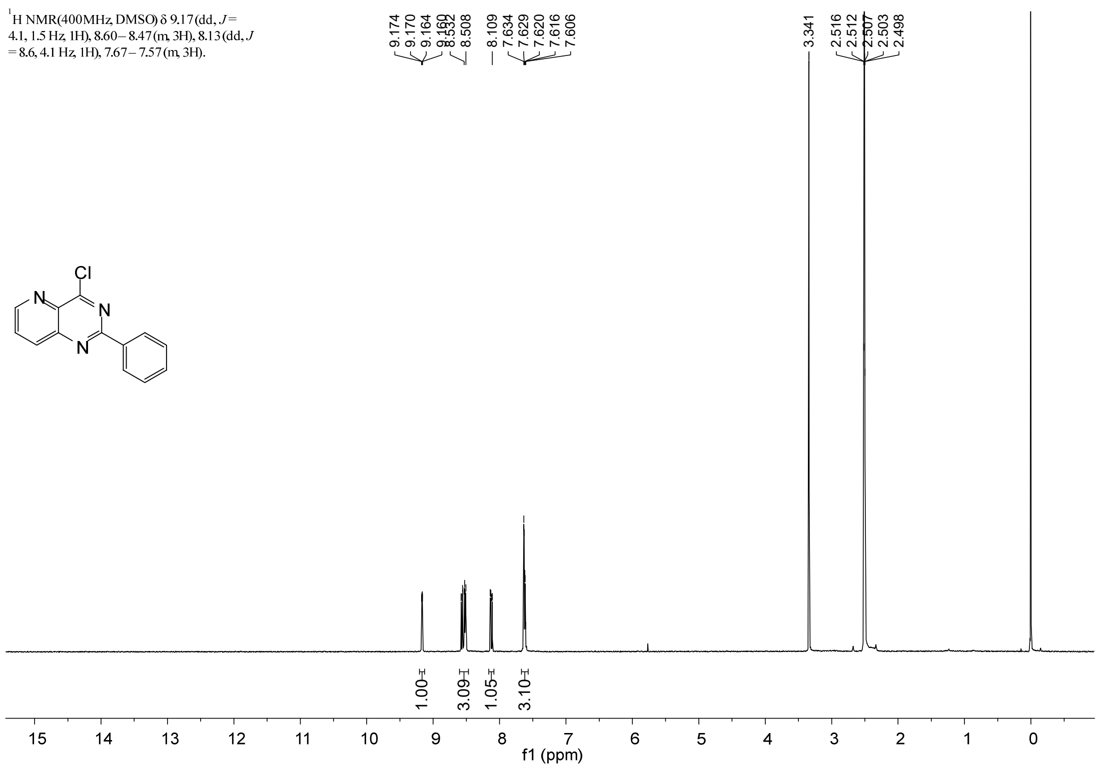

Figure S39. ^{1}H NMR (400 MHz, d_6-DMSO) of **17**

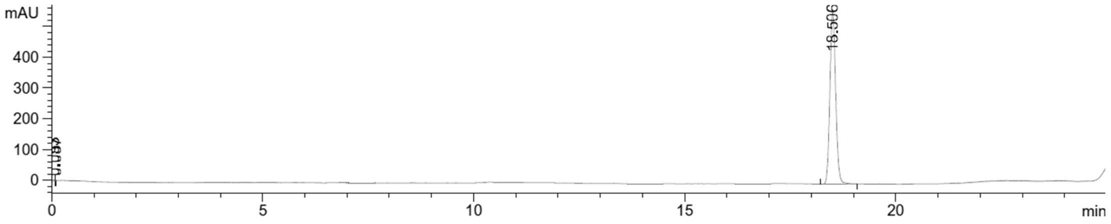

Figure S40. HPLC analysis of 18

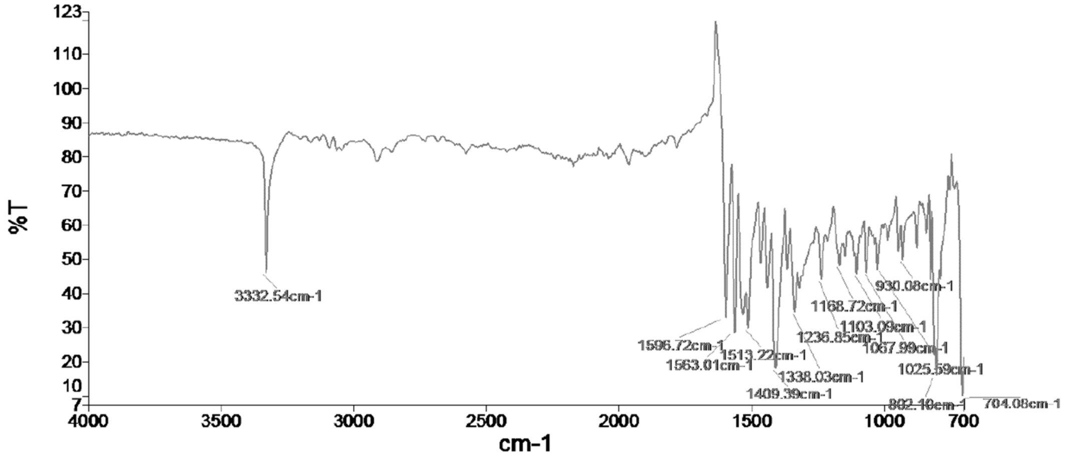

Figure S41. IR spectra of **18**

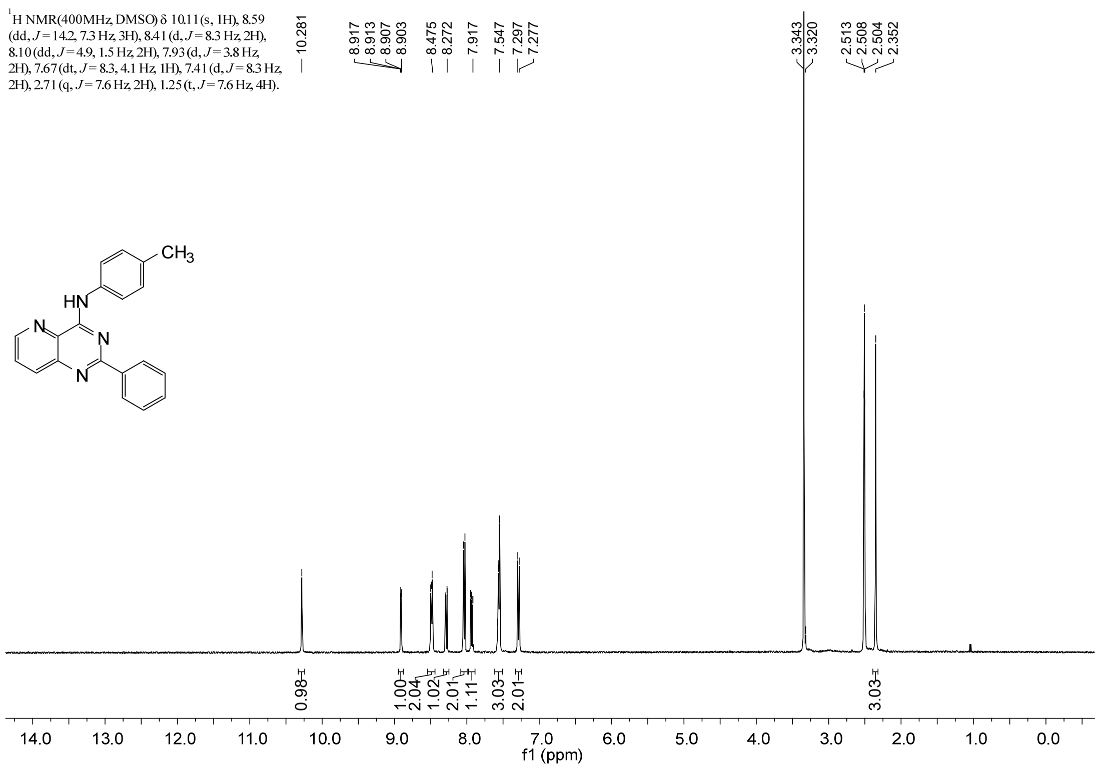

Figure S42. ^{1}H NMR (400 MHz, d_6-DMSO) of **18**

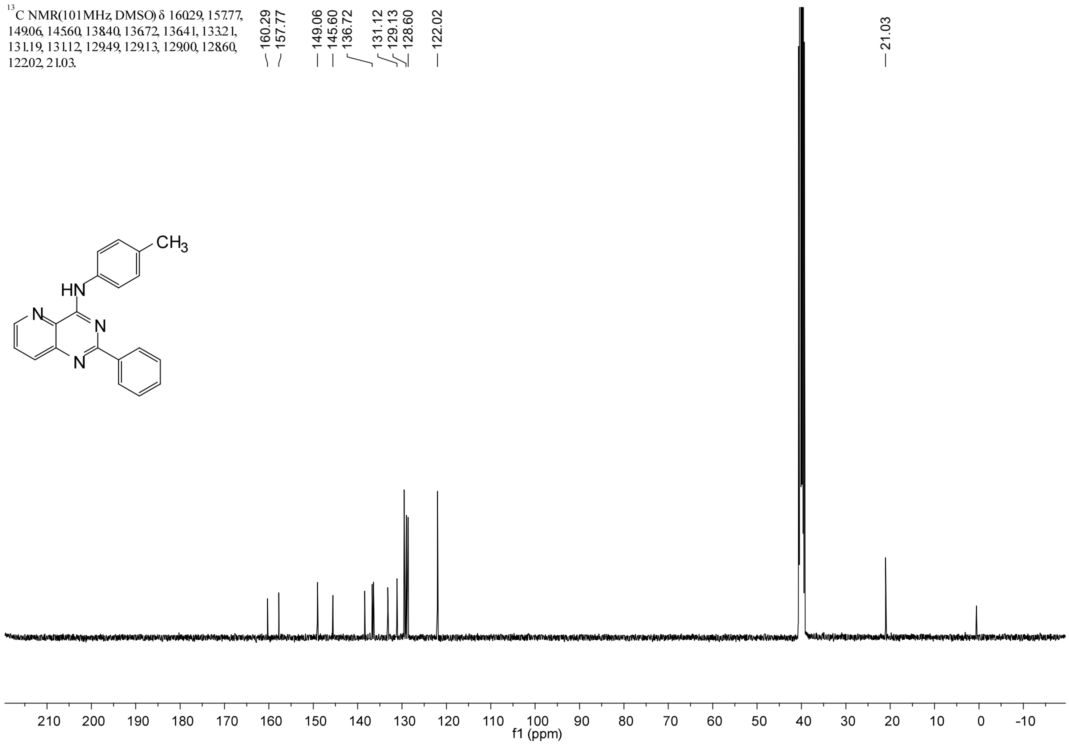

Figure S43. ^{13}C NMR (101 MHz, d_6-DMSO) of **18**

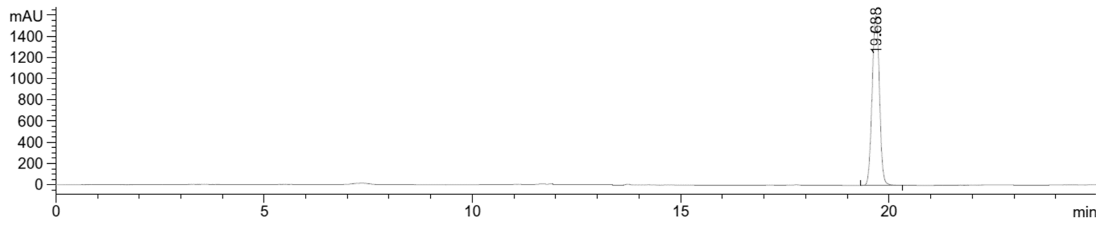

Figure S44. HPLC analysis of **19**

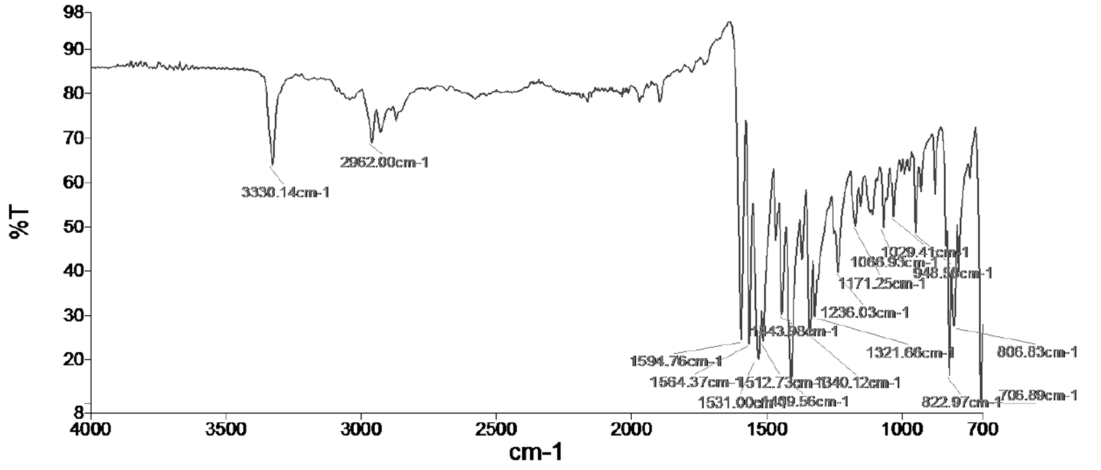

Figure S45. IR spectra of **19**

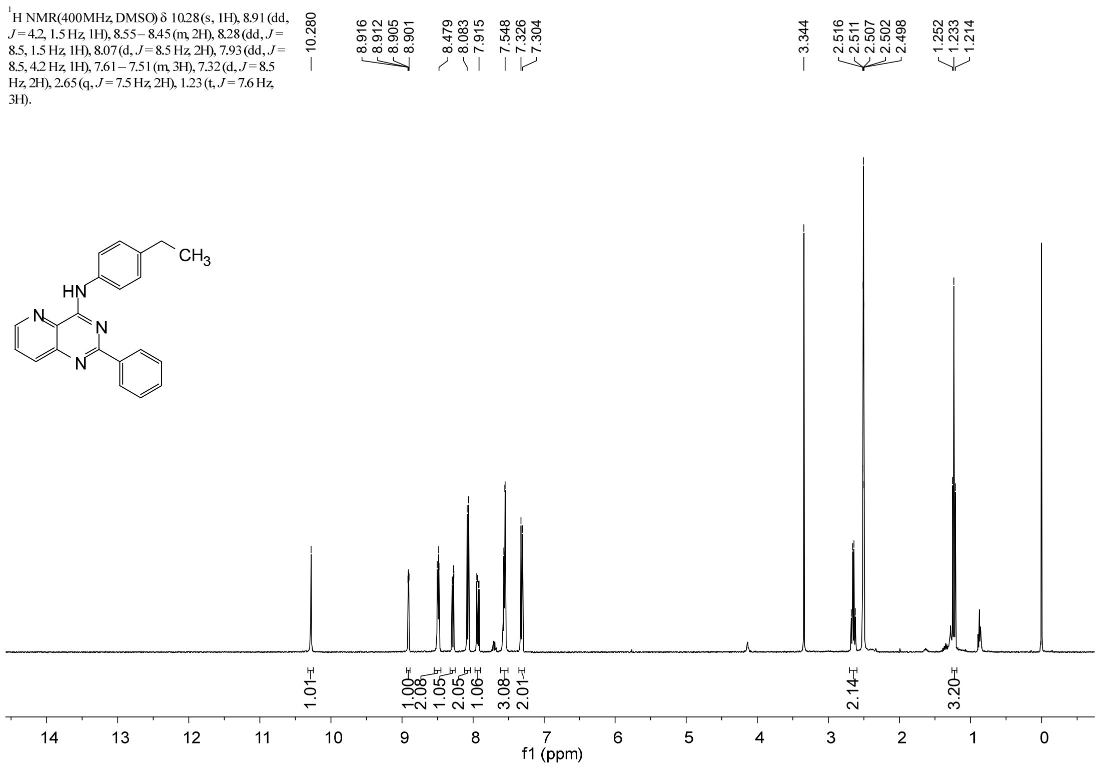

Figure S46. ^{1}H NMR (400 MHz, d_6-DMSO) of **19**

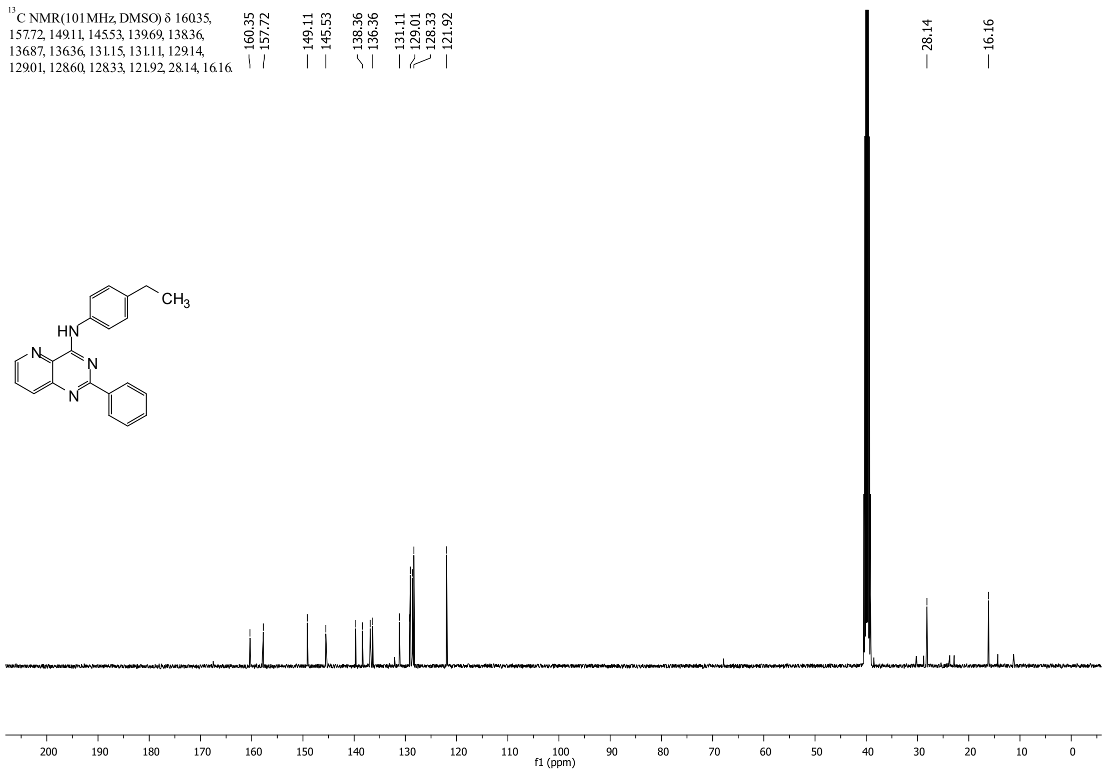

Figure S47. ^{13}C NMR (101 MHz, d_6-DMSO) of **19**

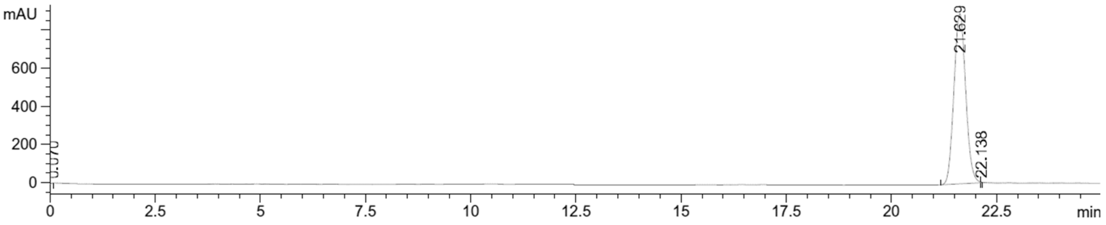

Figure S48. HPLC analysis of **20**

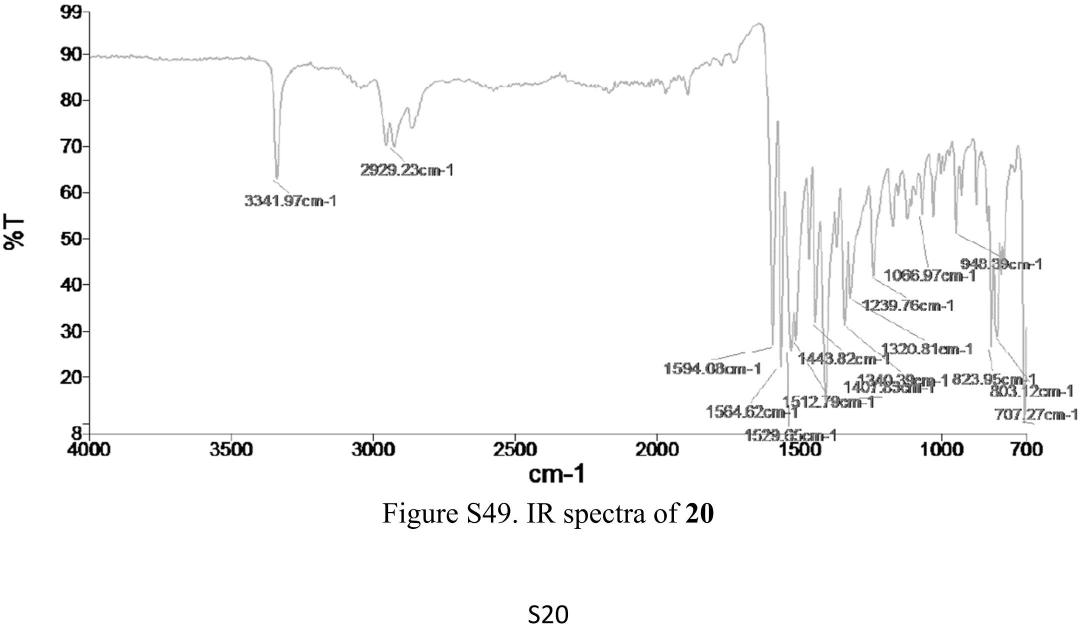

Figure S49. IR spectra of **20**

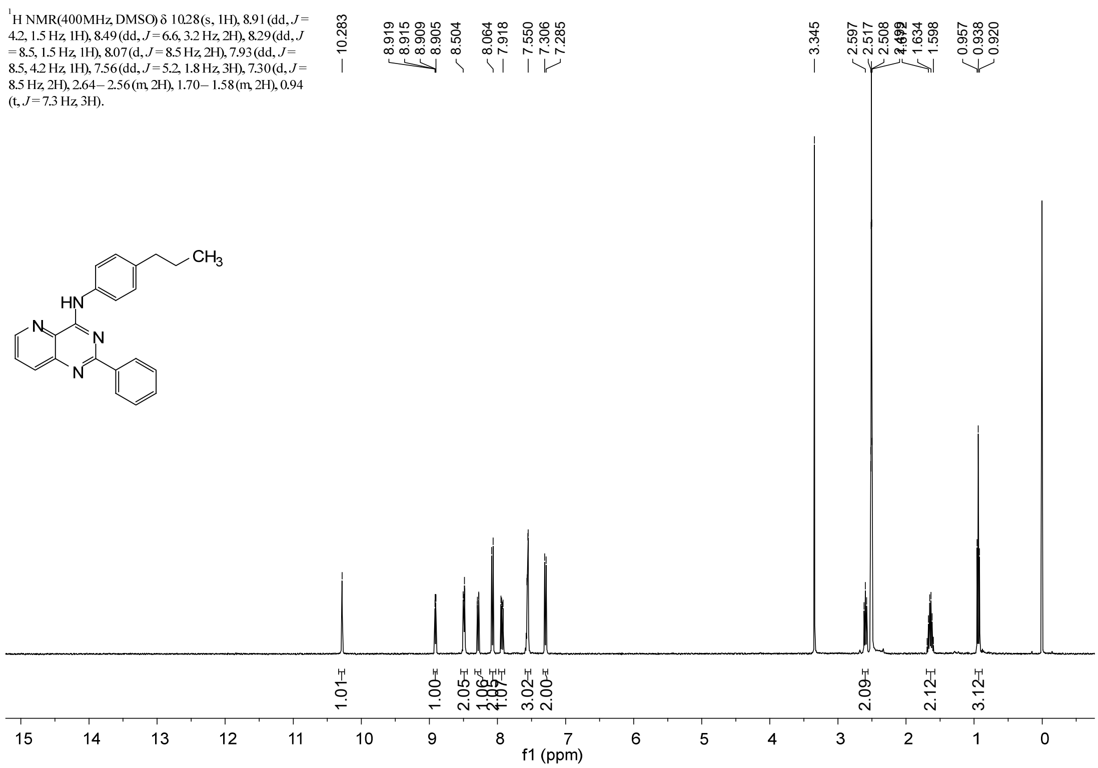

Figure S50. ^{1}H NMR (400 MHz, d_6-DMSO) of **20**

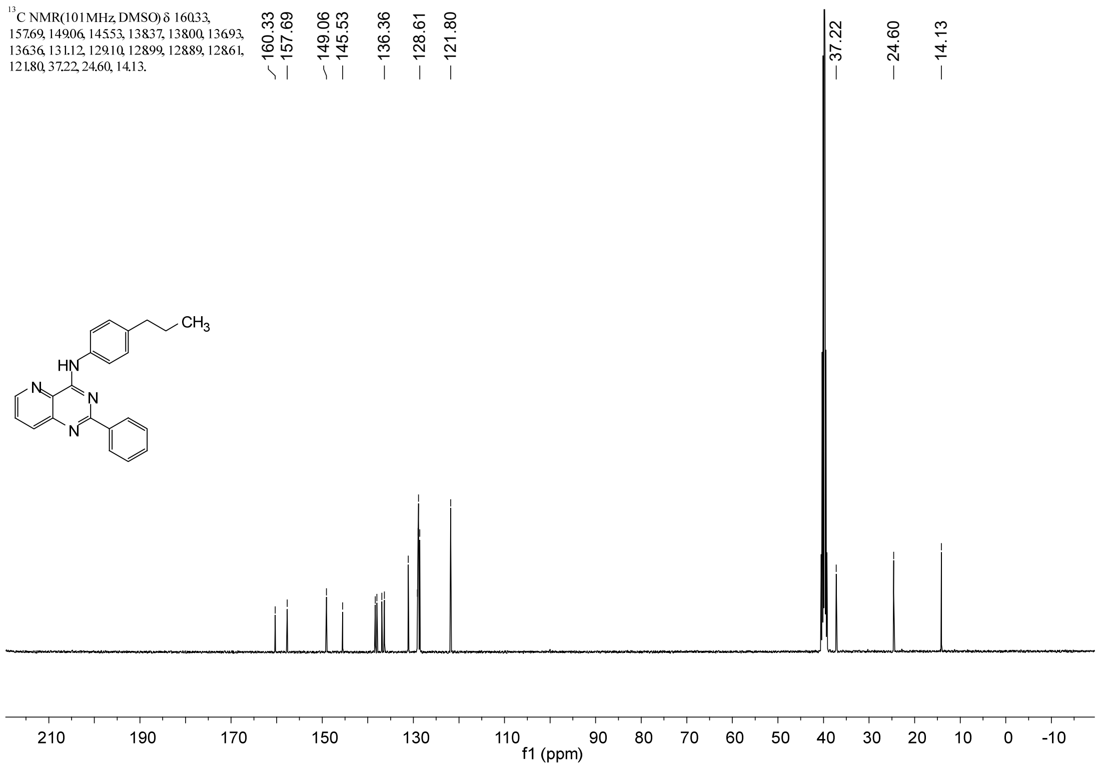

Figure S51. ^{13}C NMR (101 MHz, d_6-DMSO) of **20**

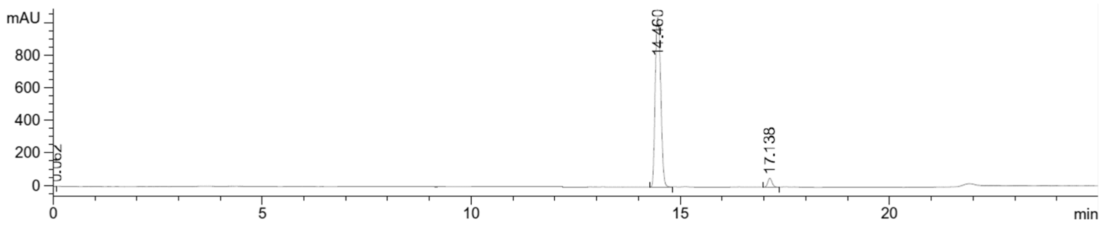

Figure S52. HPLC analysis of **21**

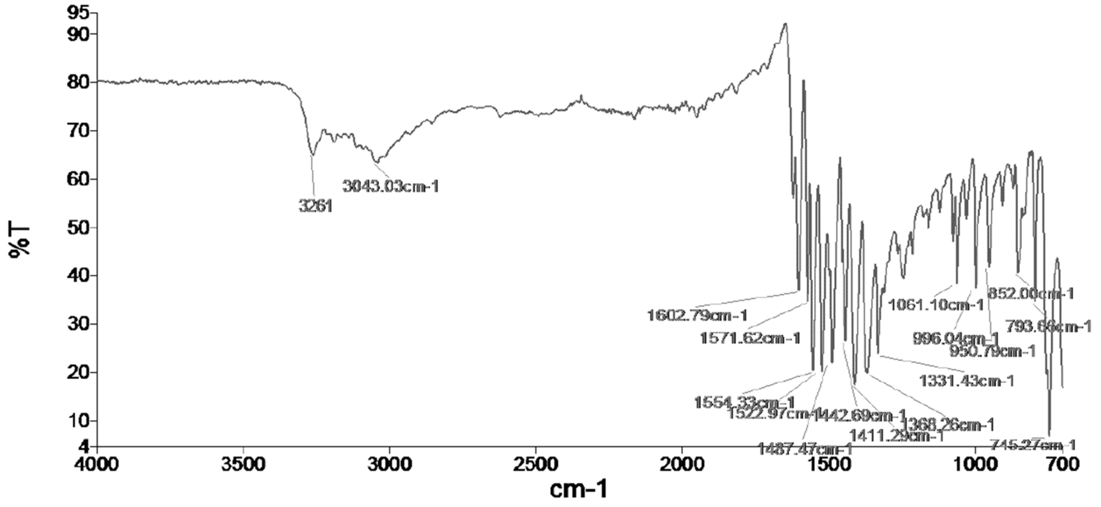

Figure S53. IR spectra of **21**

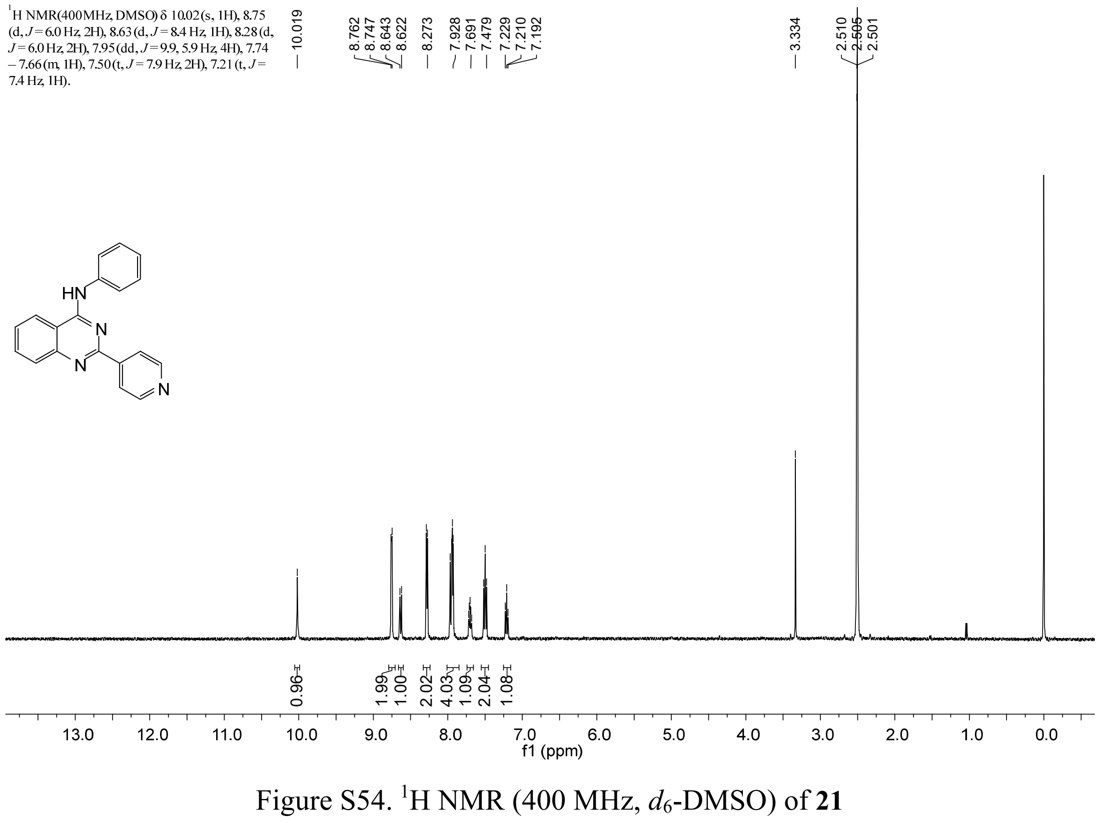

Figure S54. ^{1}H NMR (400 MHz, d_6-DMSO) of **21**

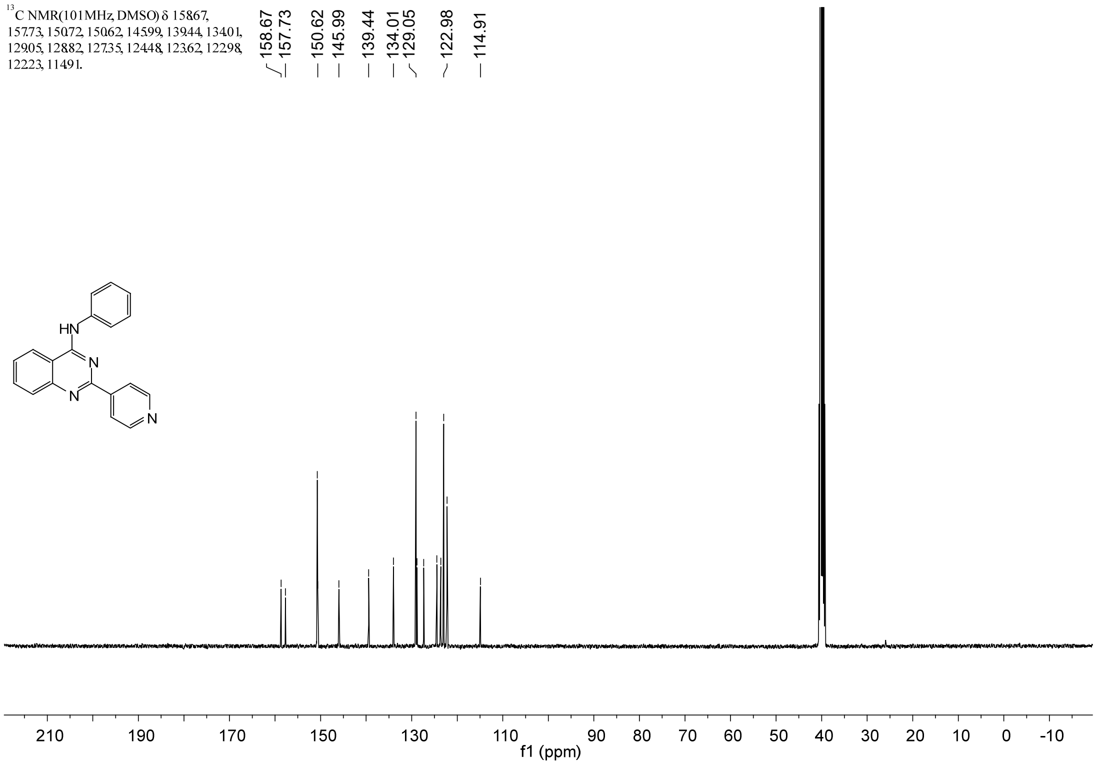

Figure S55. ^{13}C NMR (101 MHz, d_6-DMSO) of **21**

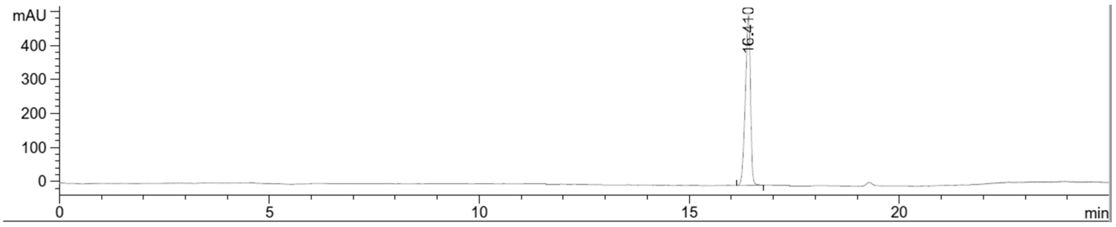

Figure S56. HPLC analysis of **22**

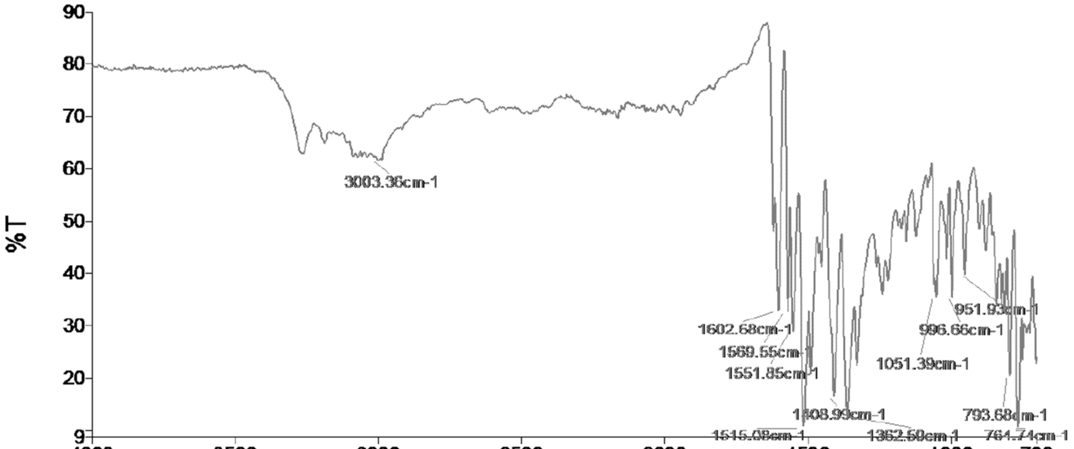

Figure S57. IR spectra of **22**

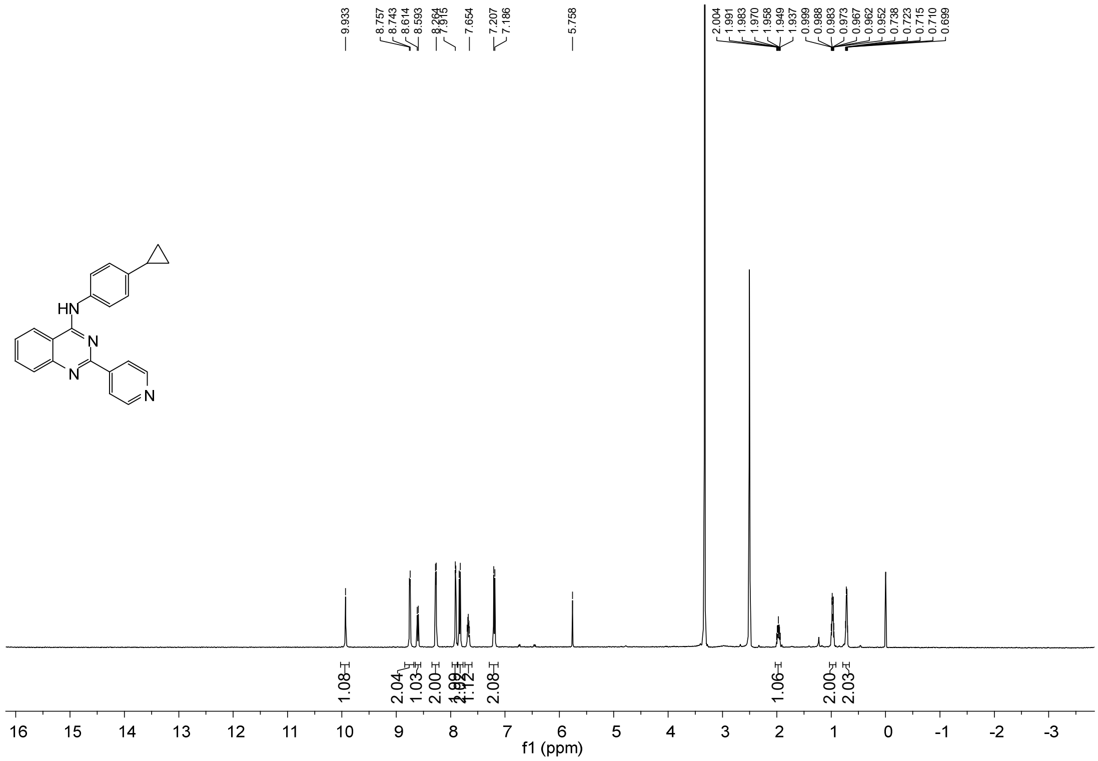

Figure S58. ^{1}H NMR (400 MHz, d_6-DMSO) of **22**

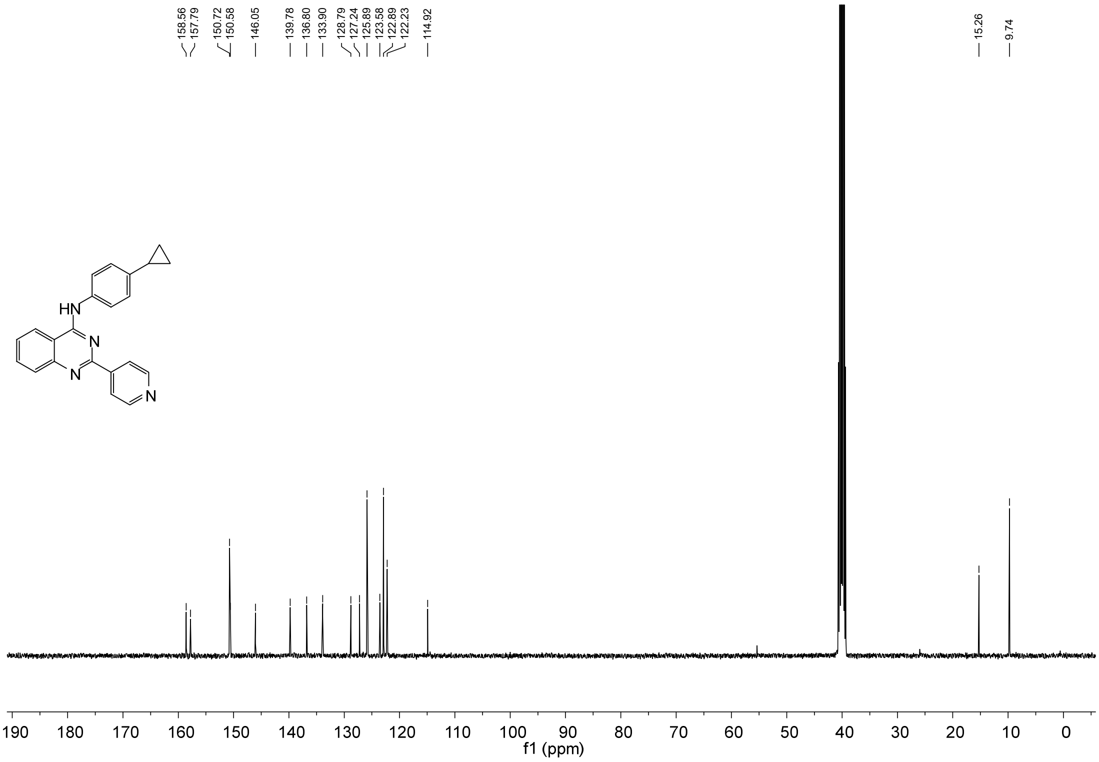

Figure S59. ^{13}C NMR (101 MHz, d_6-DMSO) of **22**

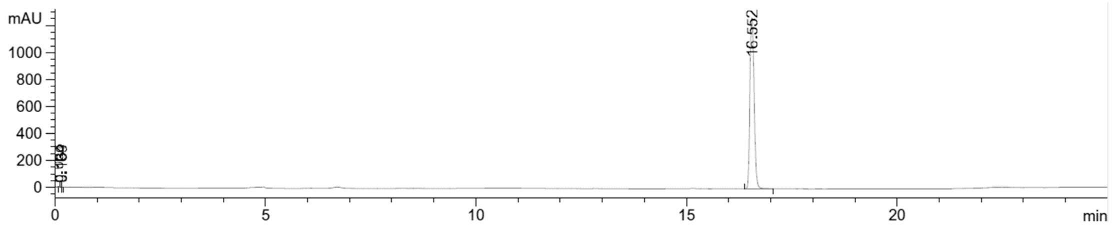

Figure S60. HPLC analysis of **23**

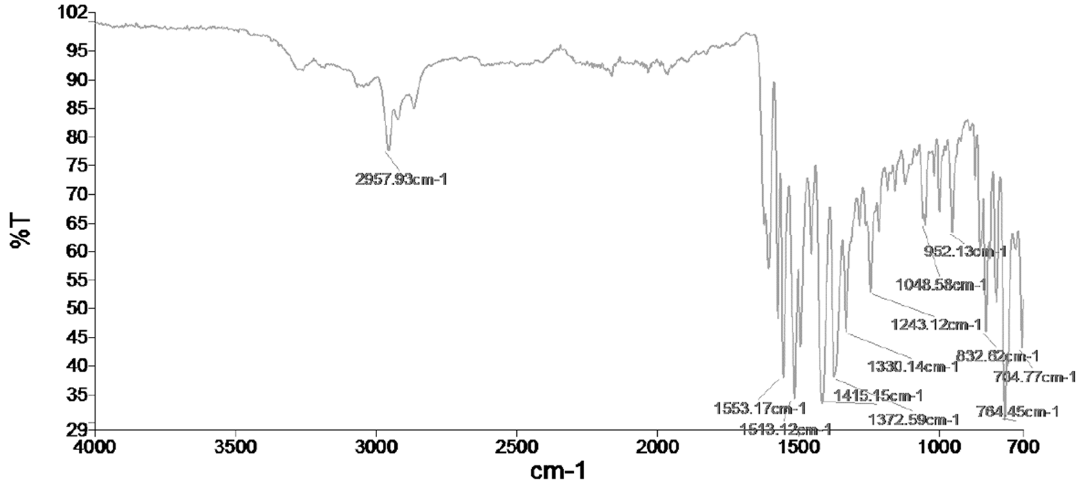

Figure S61. IR spectra of **23**

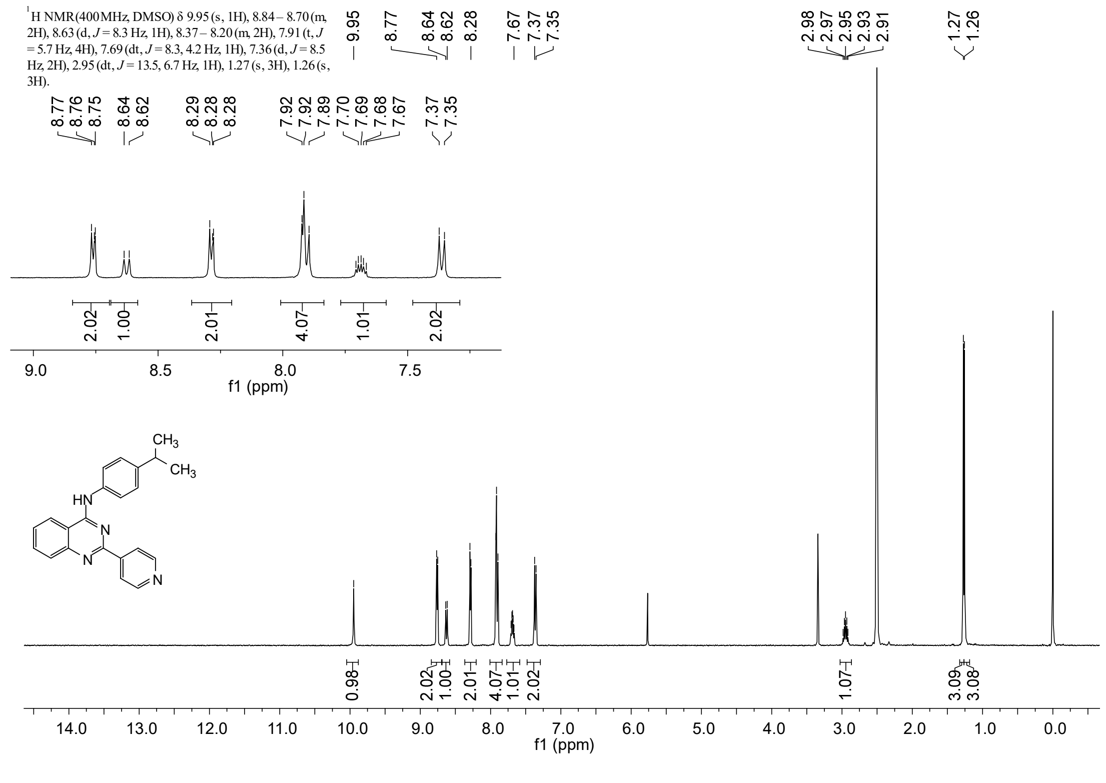

Figure S62. ^{1}H NMR (400 MHz, d_6-DMSO) of **23**

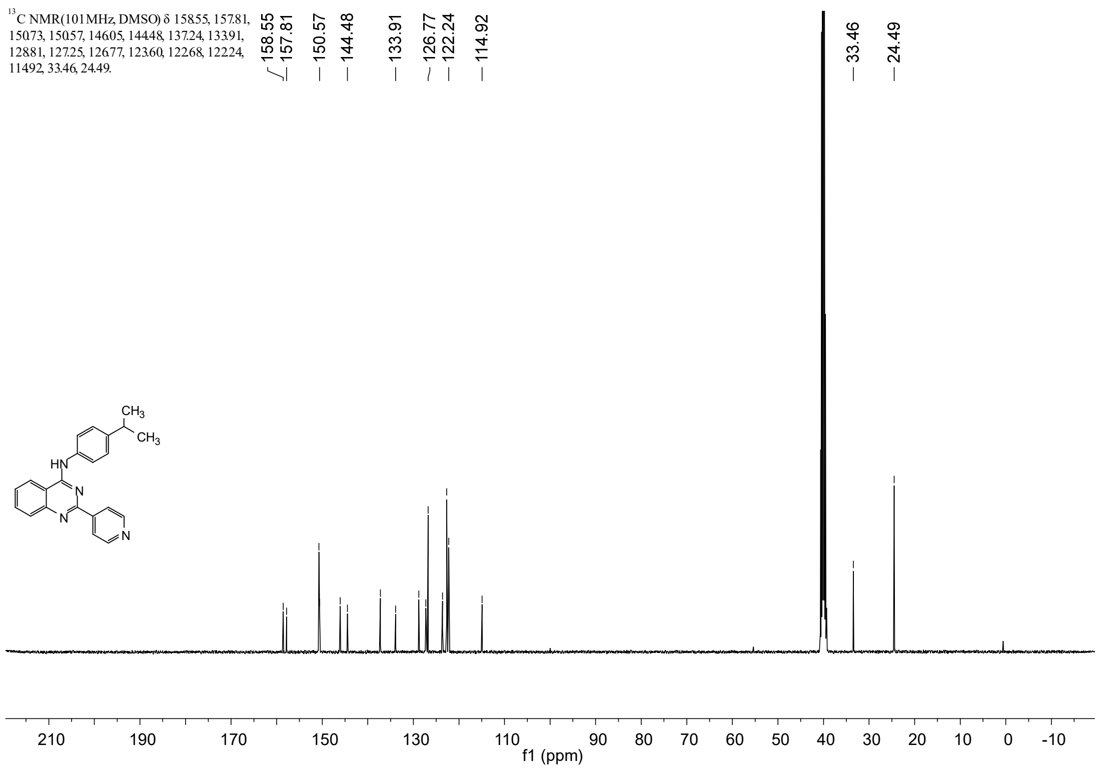

Figure S63. ^{13}C NMR (101 MHz, d_6-DMSO) of **23**

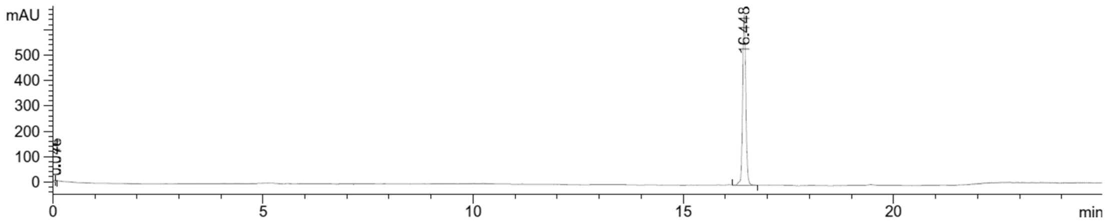

Figure S64. HPLC analysis of **24**

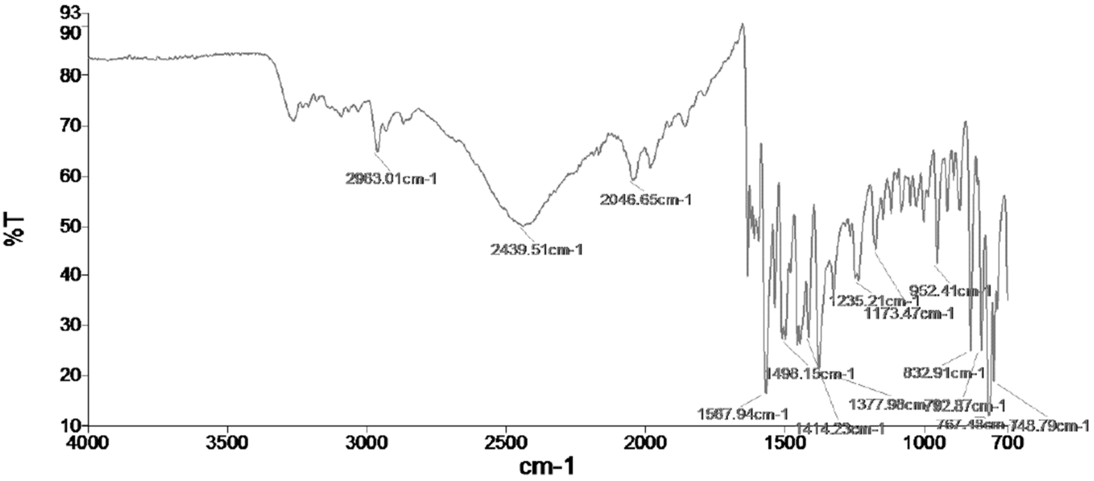

Figure S65. IR spectra of **24**

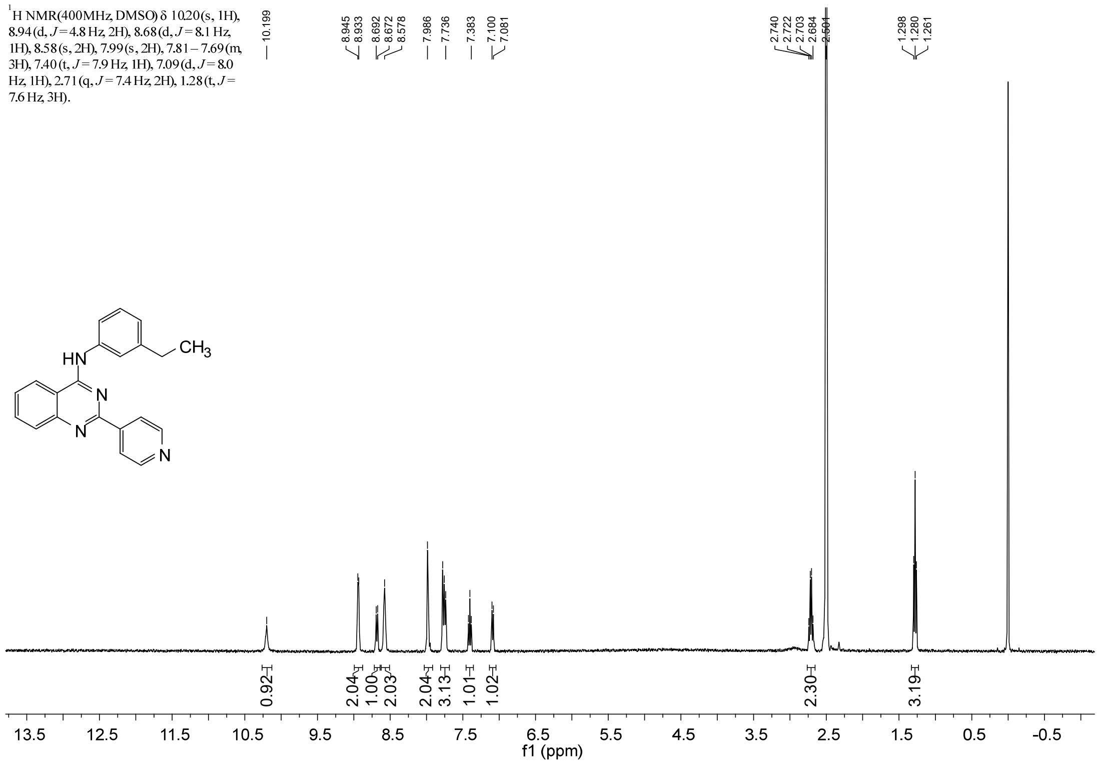

Figure S66. ^{1}H NMR (400 MHz, d_6-DMSO) of **24**

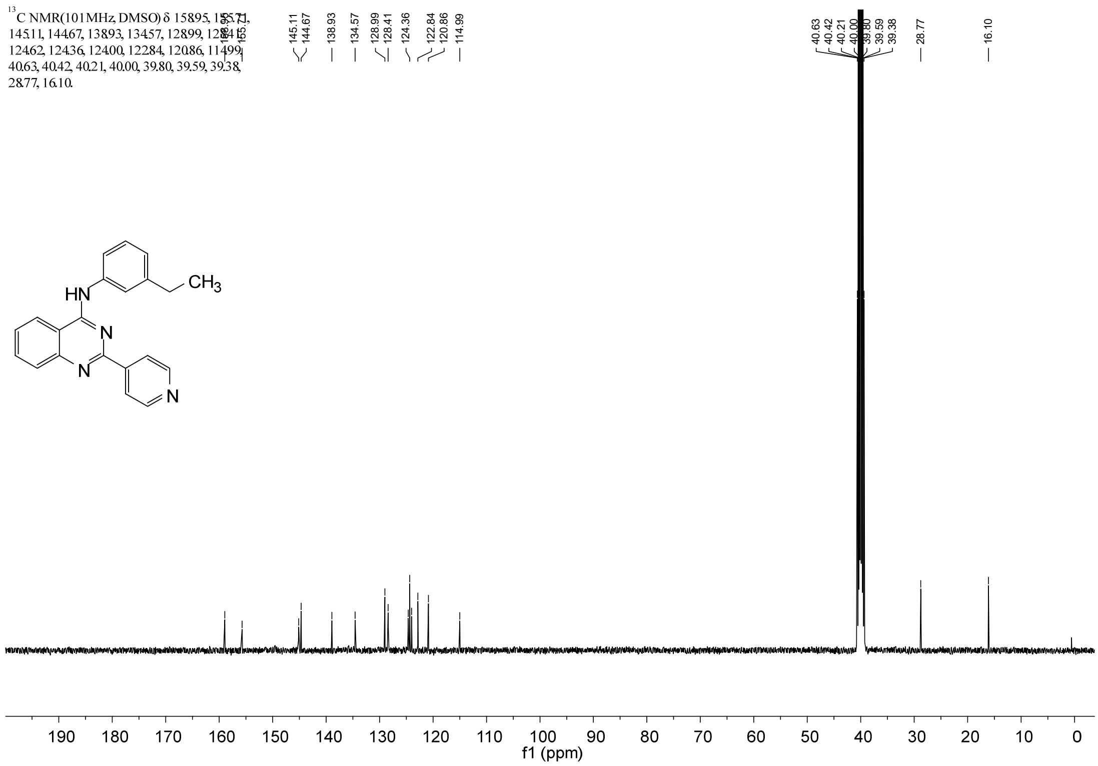

Figure S67. ^{13}C NMR (101 MHz, d_6-DMSO) of **24**

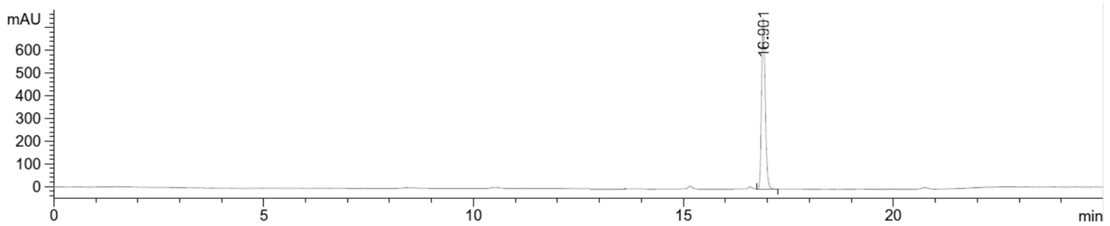

Figure S68. HPLC analysis of **25**

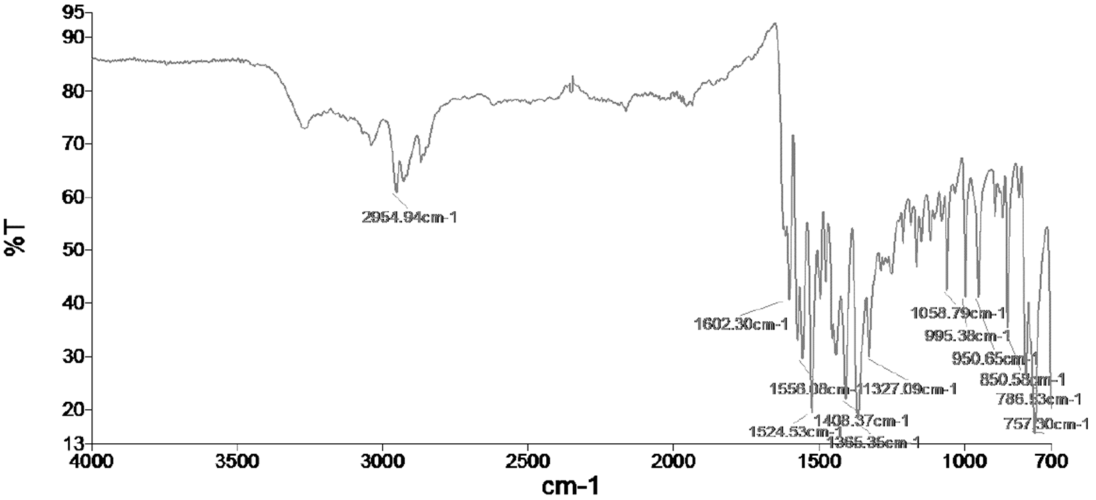

Figure S69. IR spectra of **25**

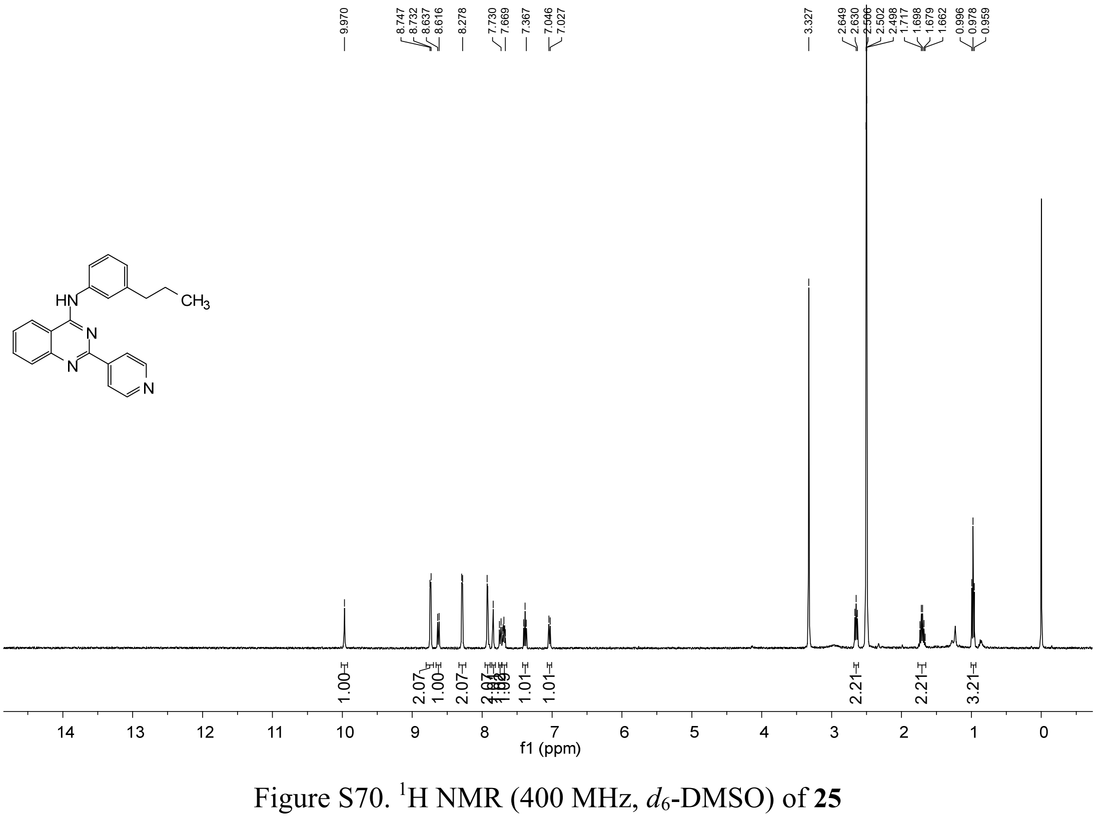

Figure S70. ^{1}H NMR (400 MHz, d_6-DMSO) of **25**

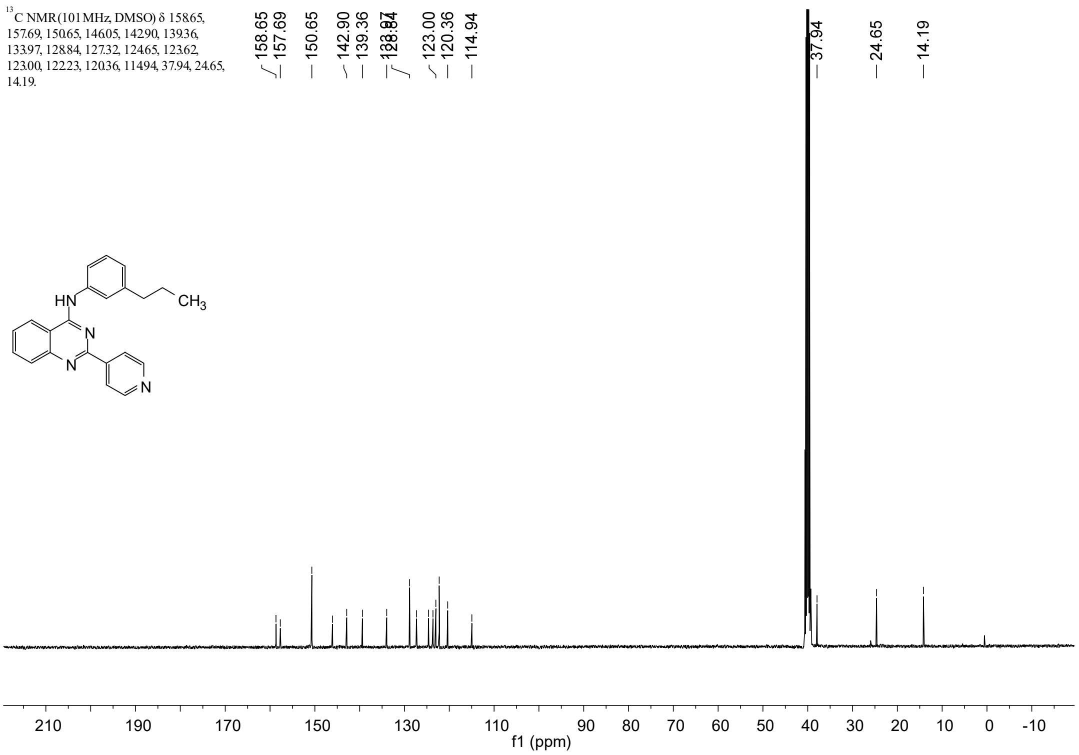

Figure S71. ^{13}C NMR (101 MHz, d_6-DMSO) of **25**

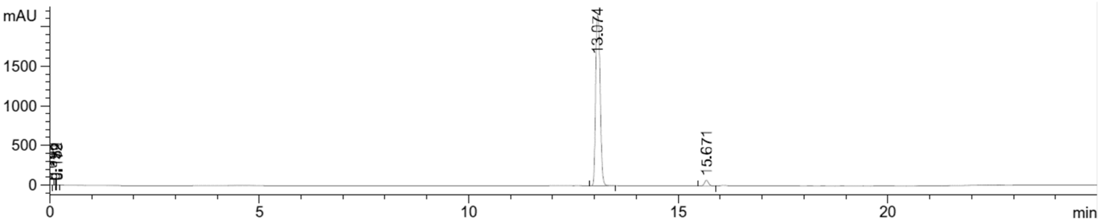

Figure S72. HPLC analysis of **26**

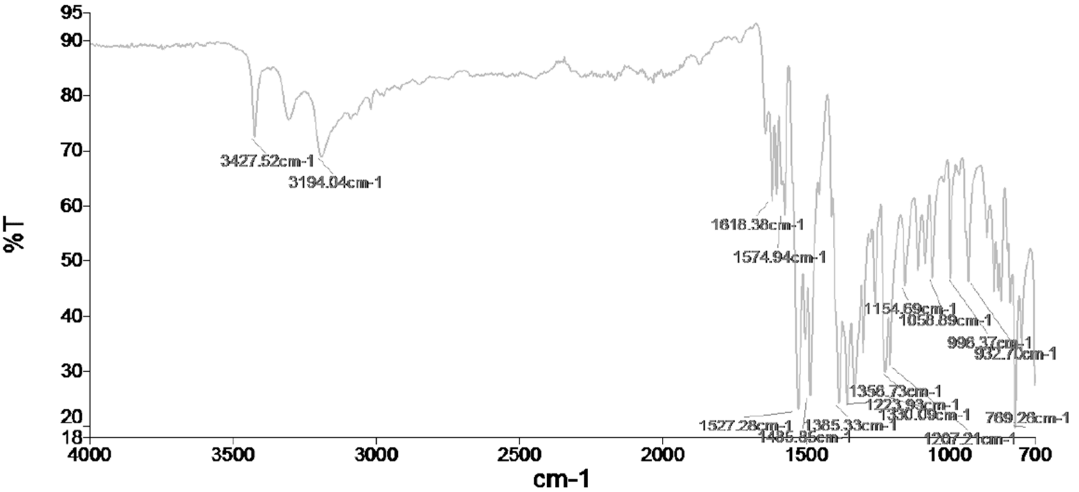

Figure S73. IR spectra of **26**

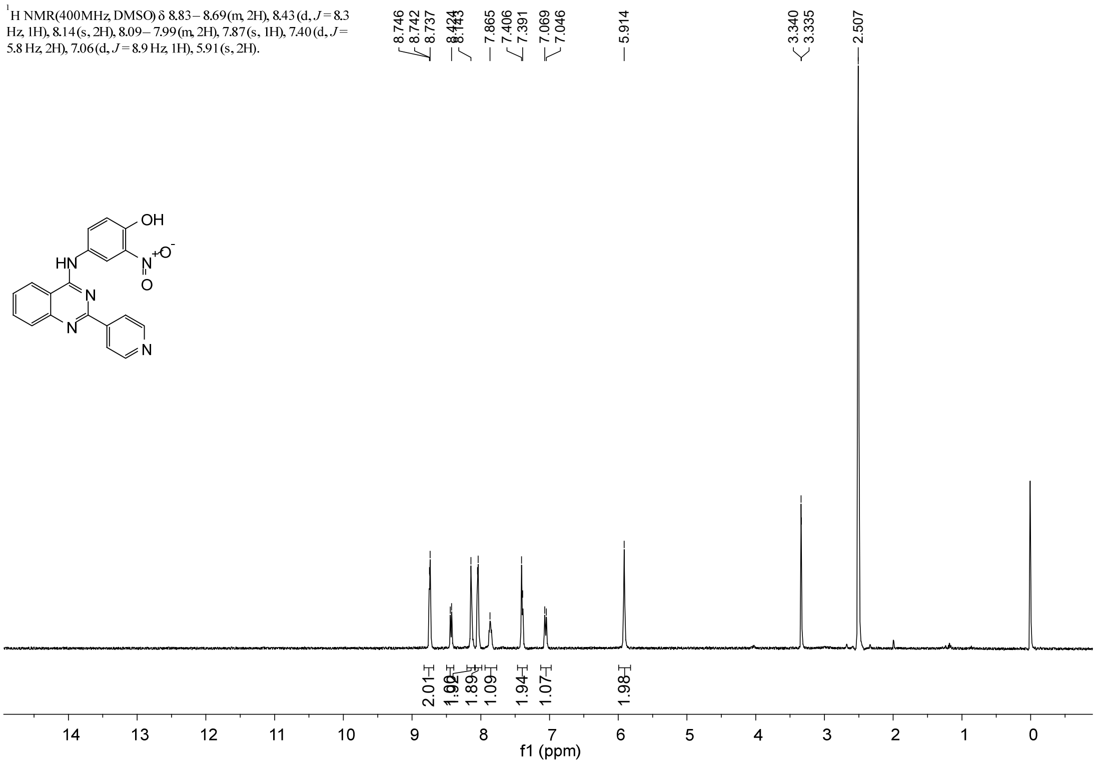

Figure S74. ^{1}H NMR (400 MHz, d_6-DMSO) of **26**

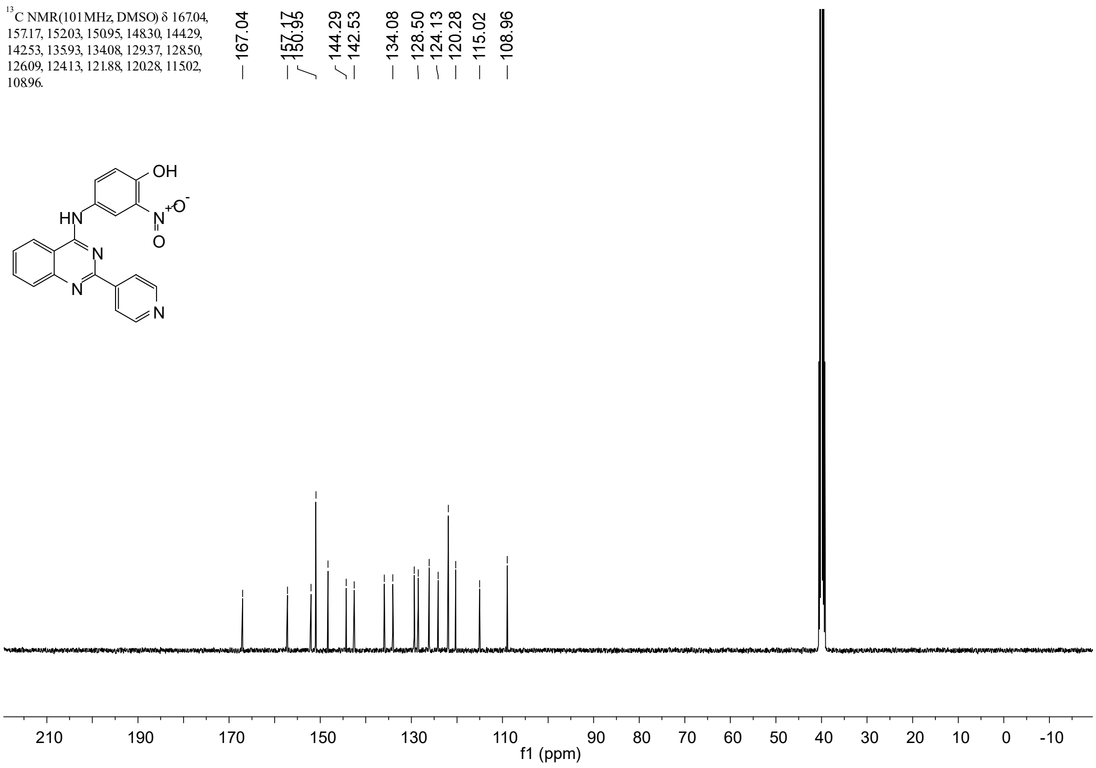

Figure S75. ^{13}C NMR (101 MHz, d_6-DMSO) of **26**

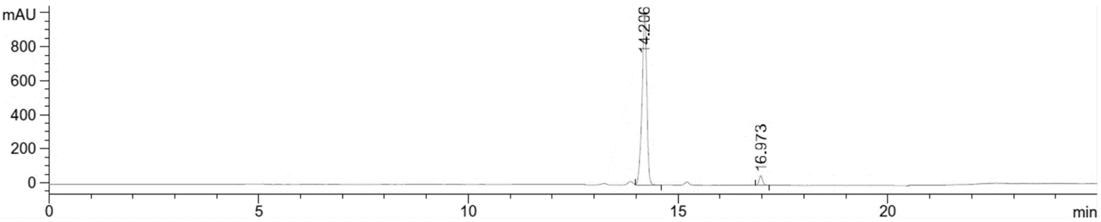

Figure S76. HPLC analysis of **27**

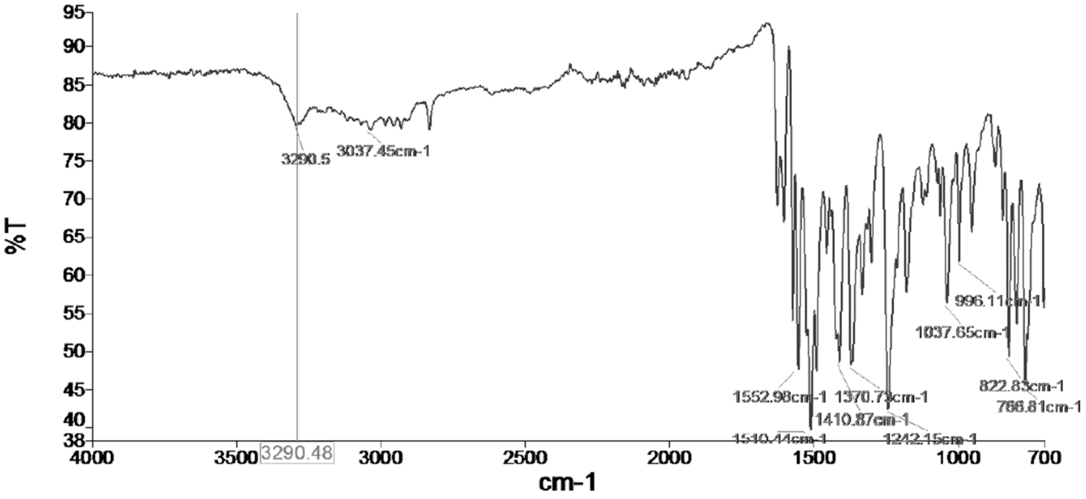

Figure S77. IR spectra of **27**

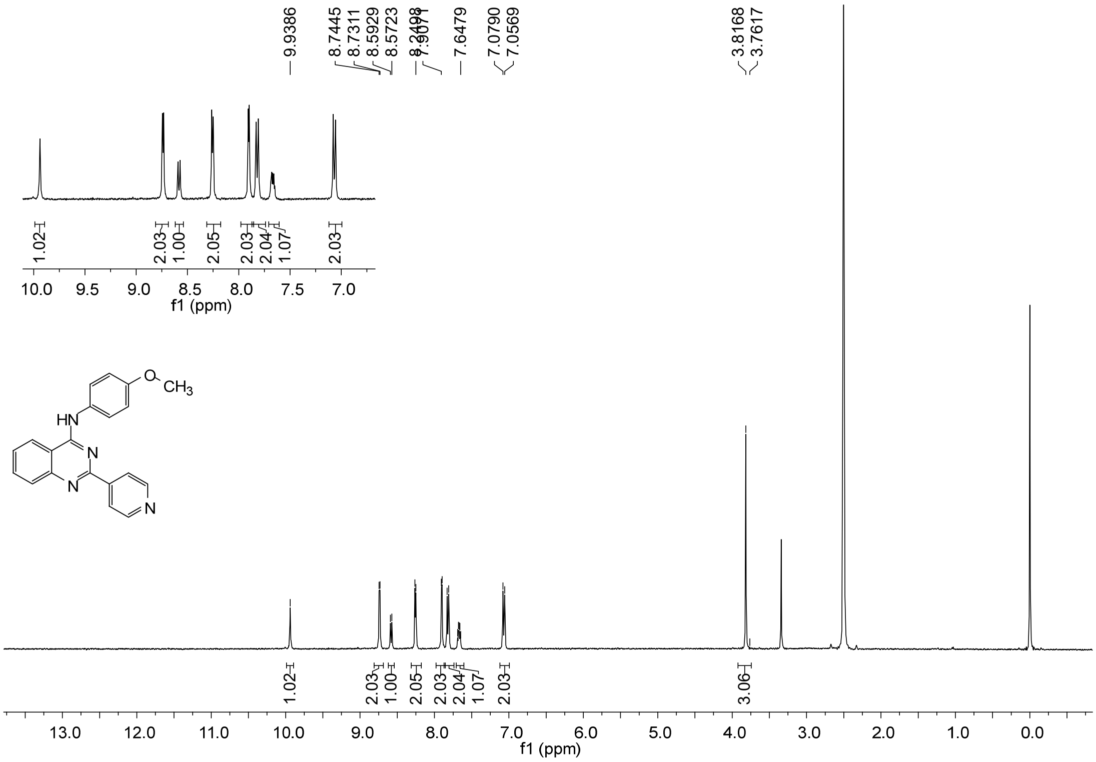

Figure S78. ^{1}H NMR (400 MHz, d_6-DMSO) of **27**

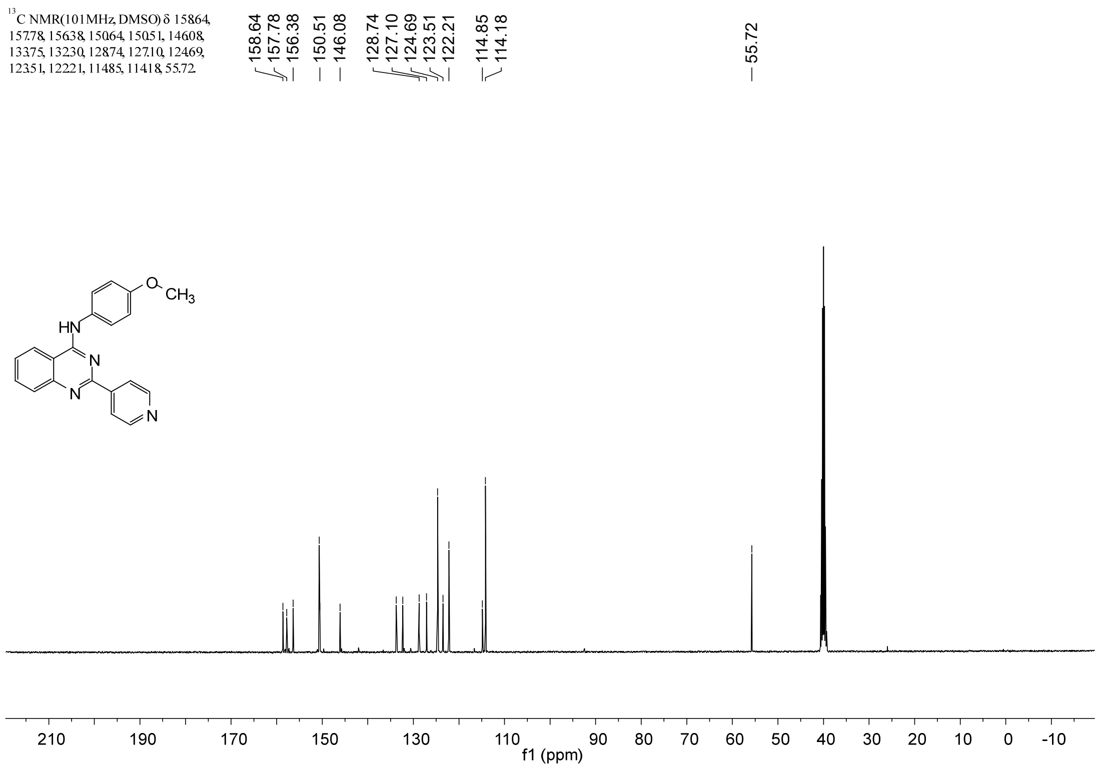

Figure S79. ^{13}C NMR (101 MHz, DMSO) of **27**

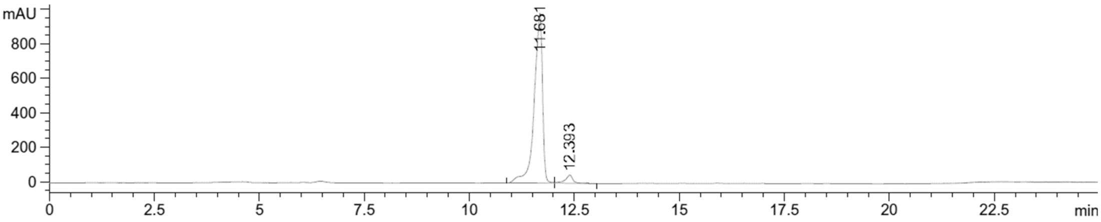

Figure S80. HPLC analysis of **28**

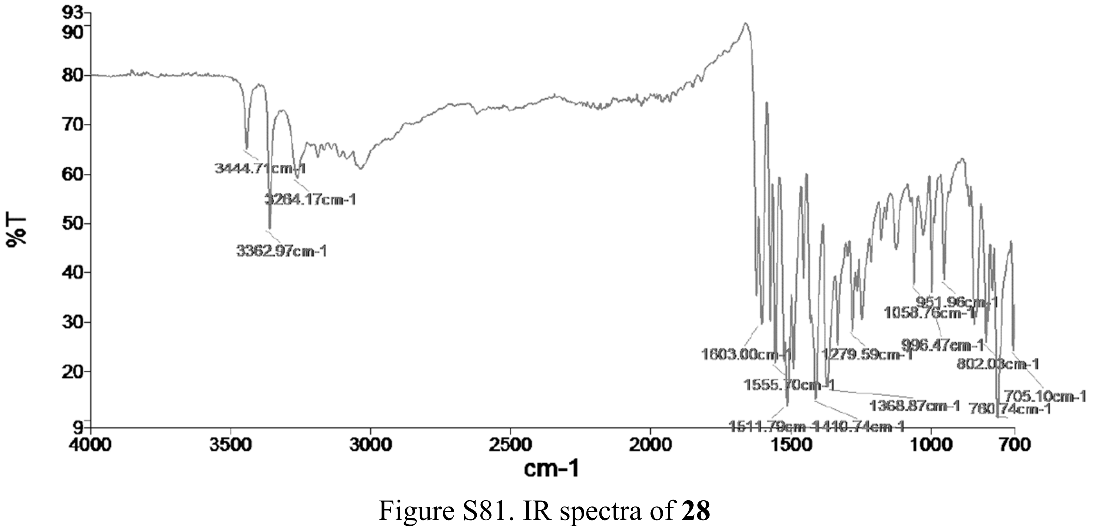

Figure S81. IR spectra of **28**

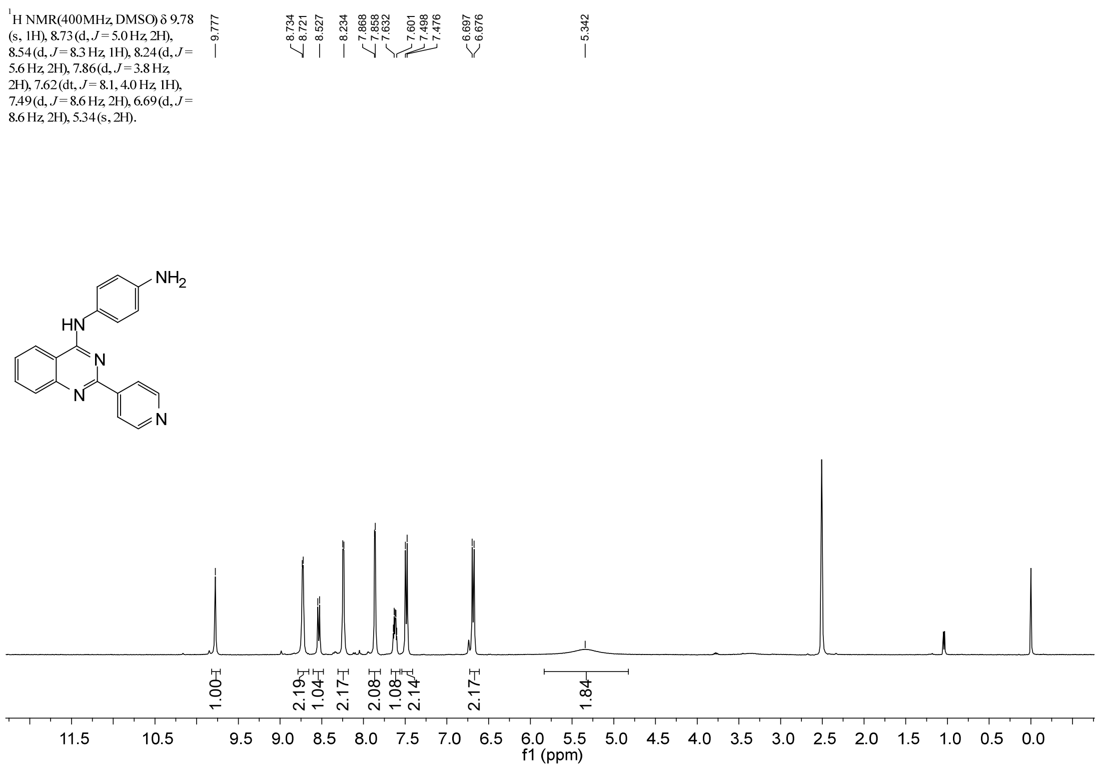

Figure S82. ^{1}H NMR (400 MHz, d_6-DMSO) of **28**

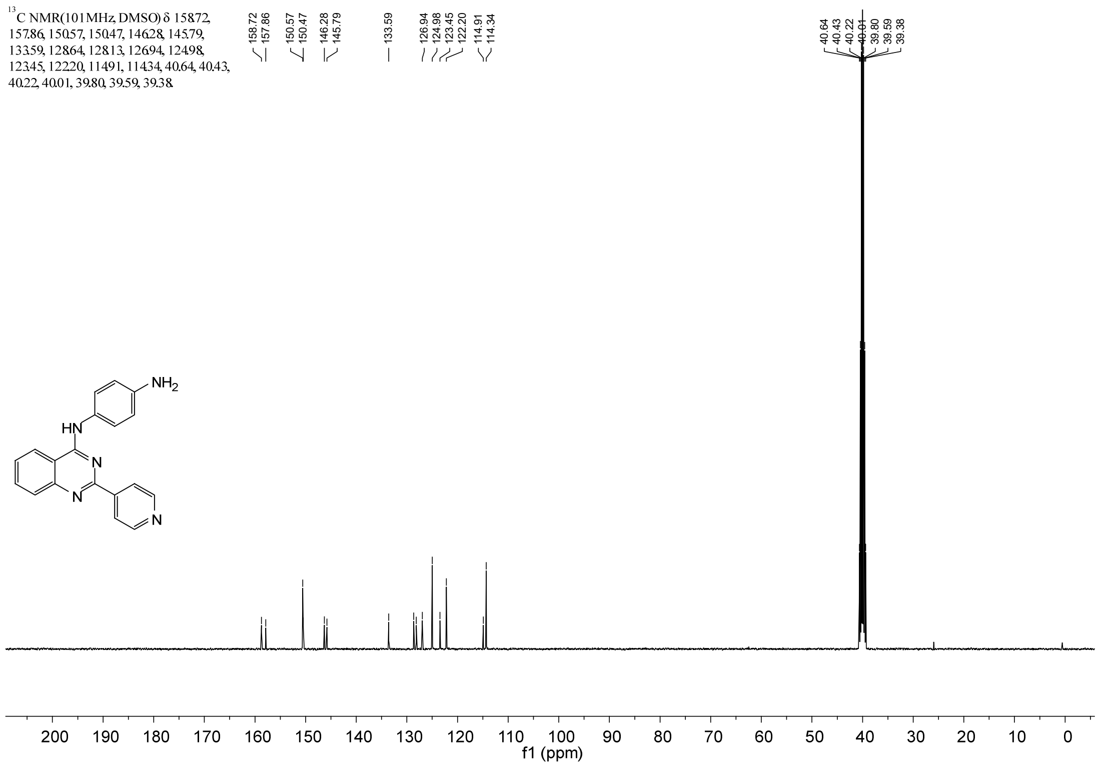

Figure S83. ^{13}C NMR (101 MHz, d_6-DMSO) of **28**

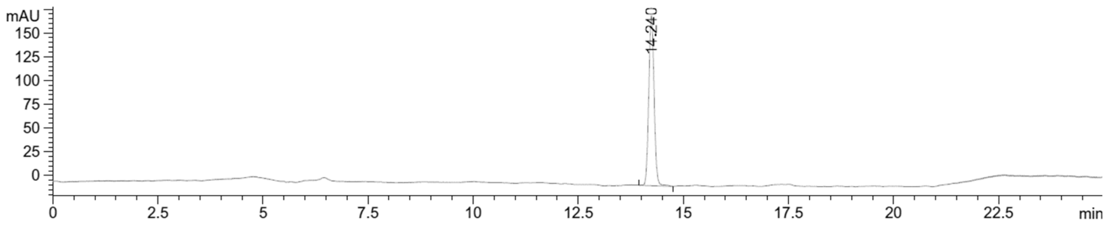

Figure S84. HPLC analysis of **29**

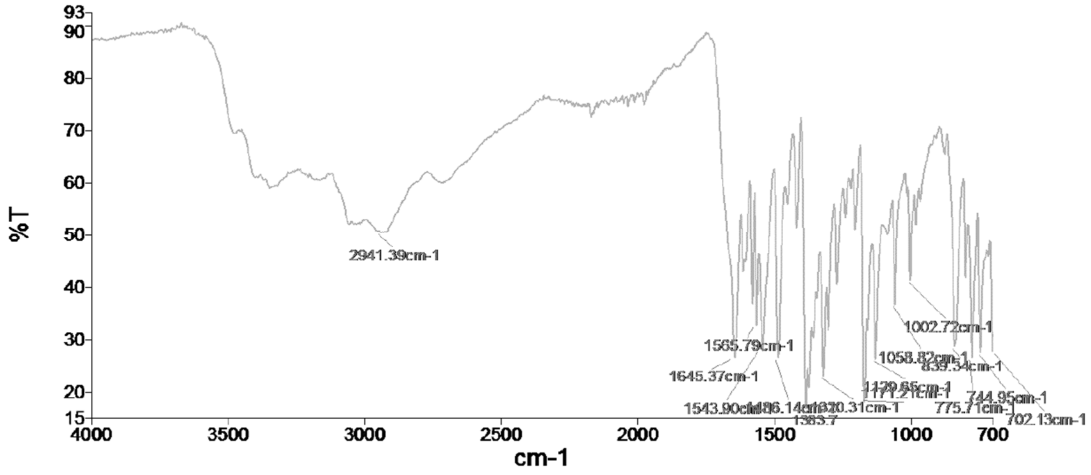

Figure S85. IR spectra of **29**

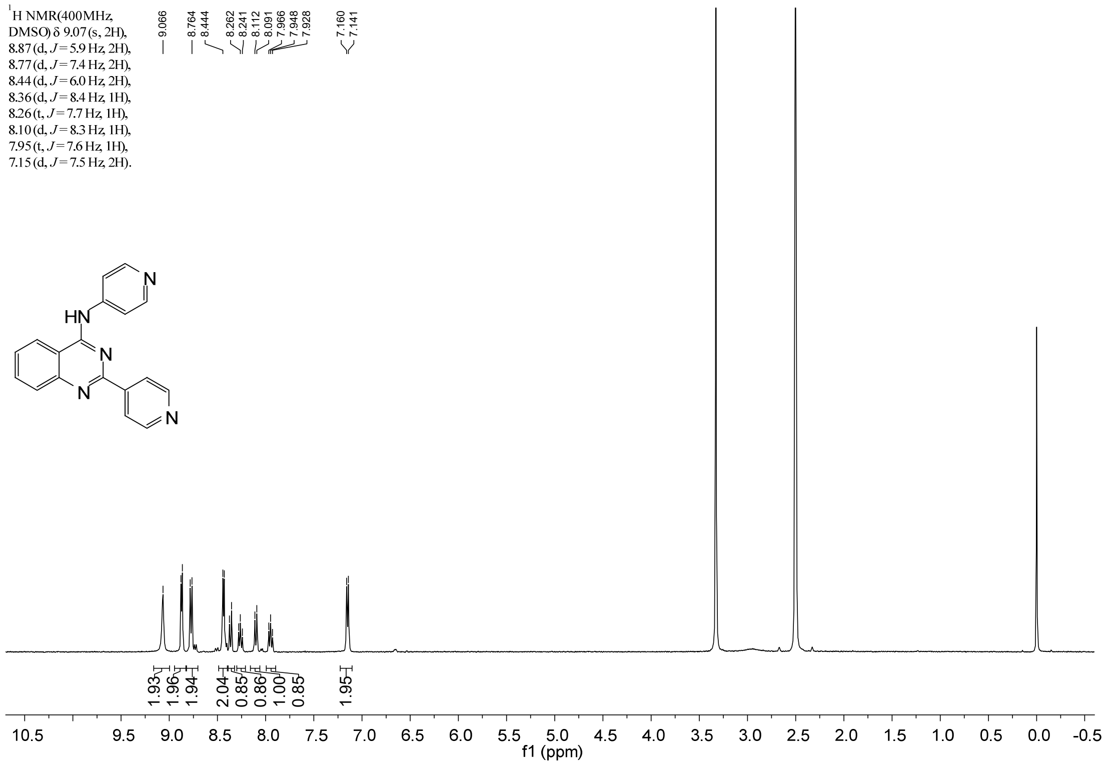

Figure S86. ^{1}H NMR (400 MHz, d_6-DMSO) of **29**

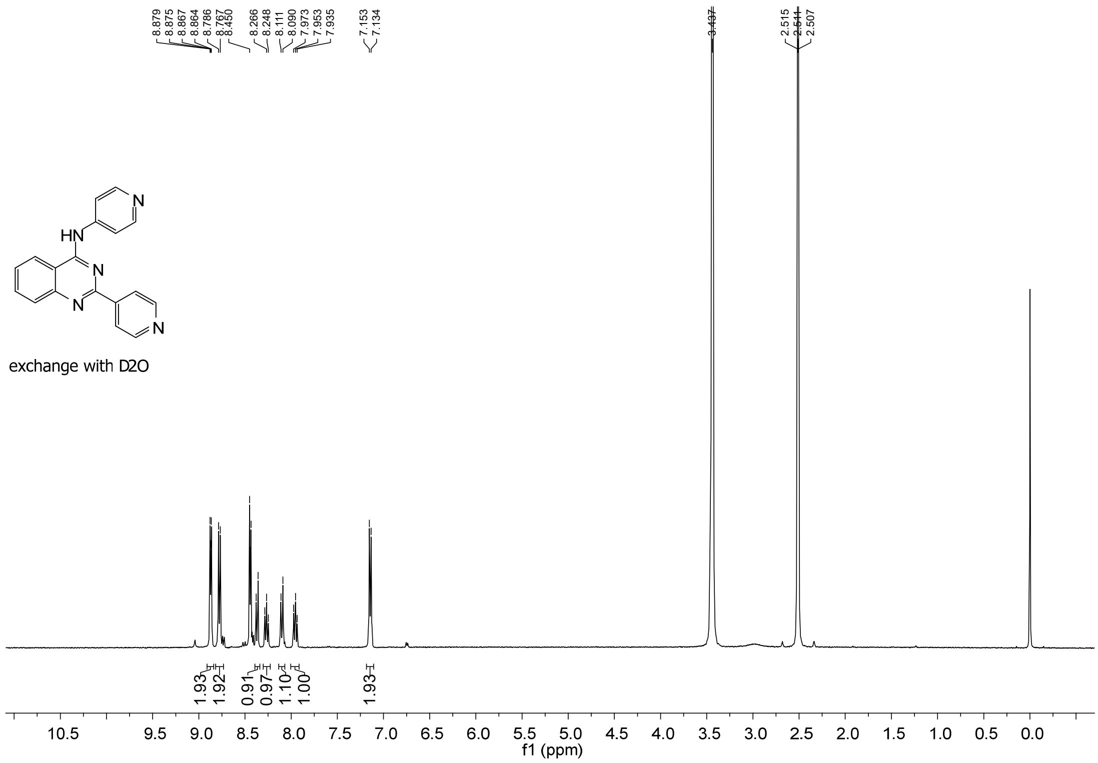

Figure S87. ^{1}H NMR (400 MHz, d_6-DMSO) of **29**, exchange with D_2O

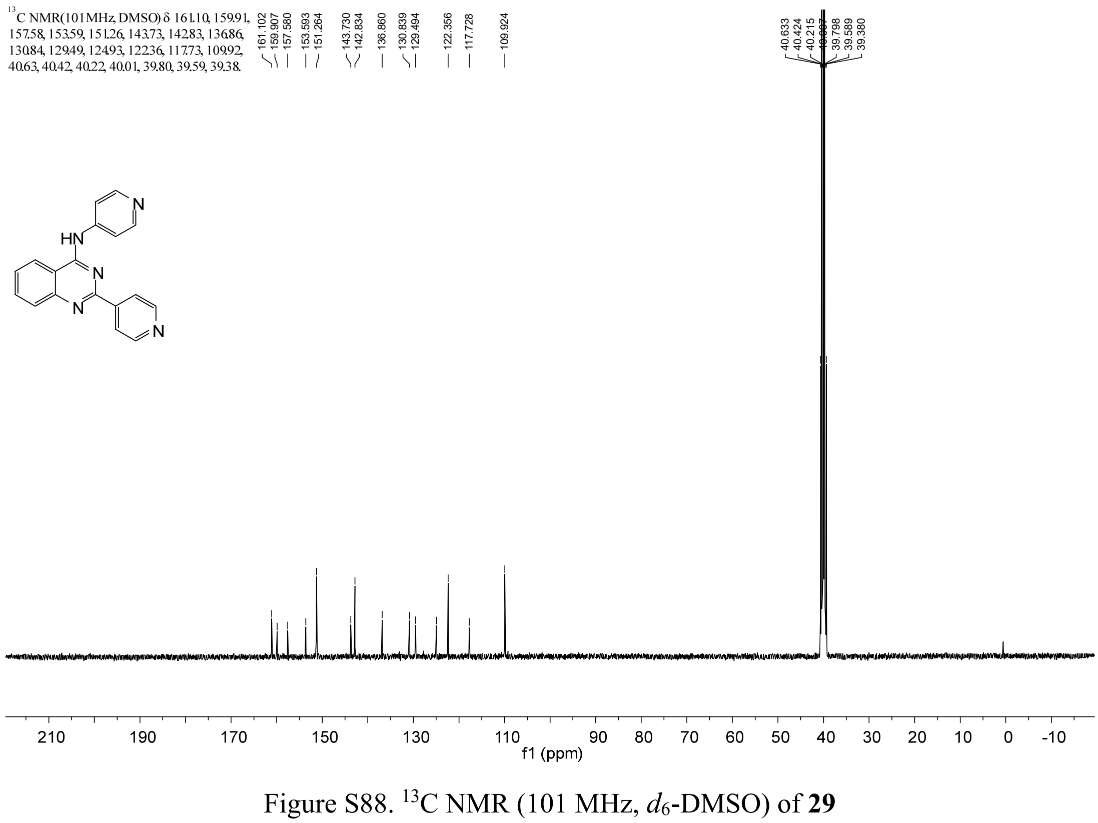

Figure S88. ^{13}C NMR (101 MHz, d_6-DMSO) of **29**

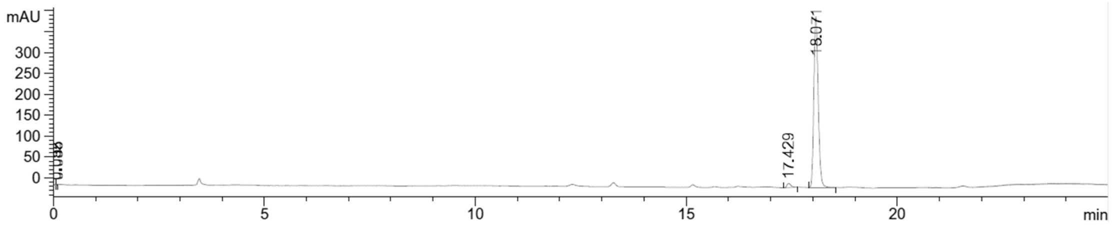

Figure S89. HPLC analysis of **30**

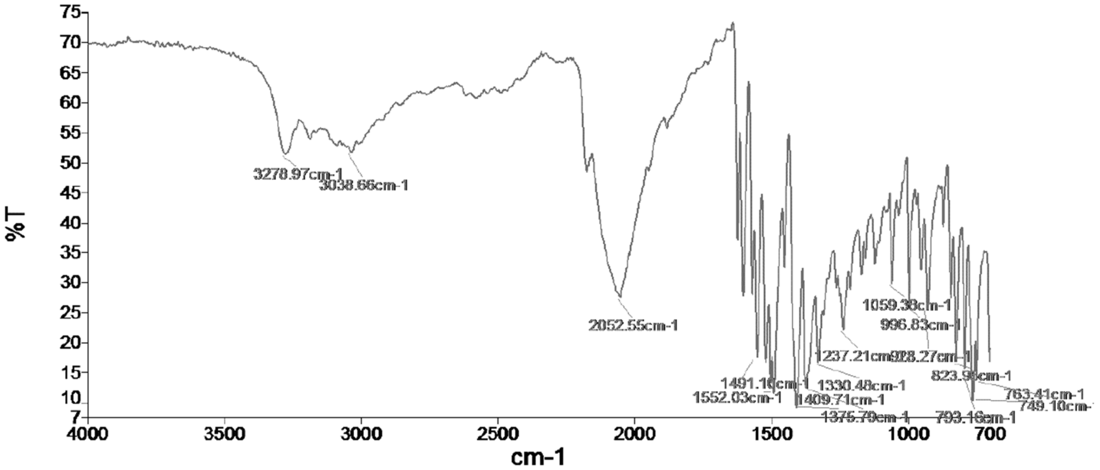

Figure S90. IR spectra of **30**

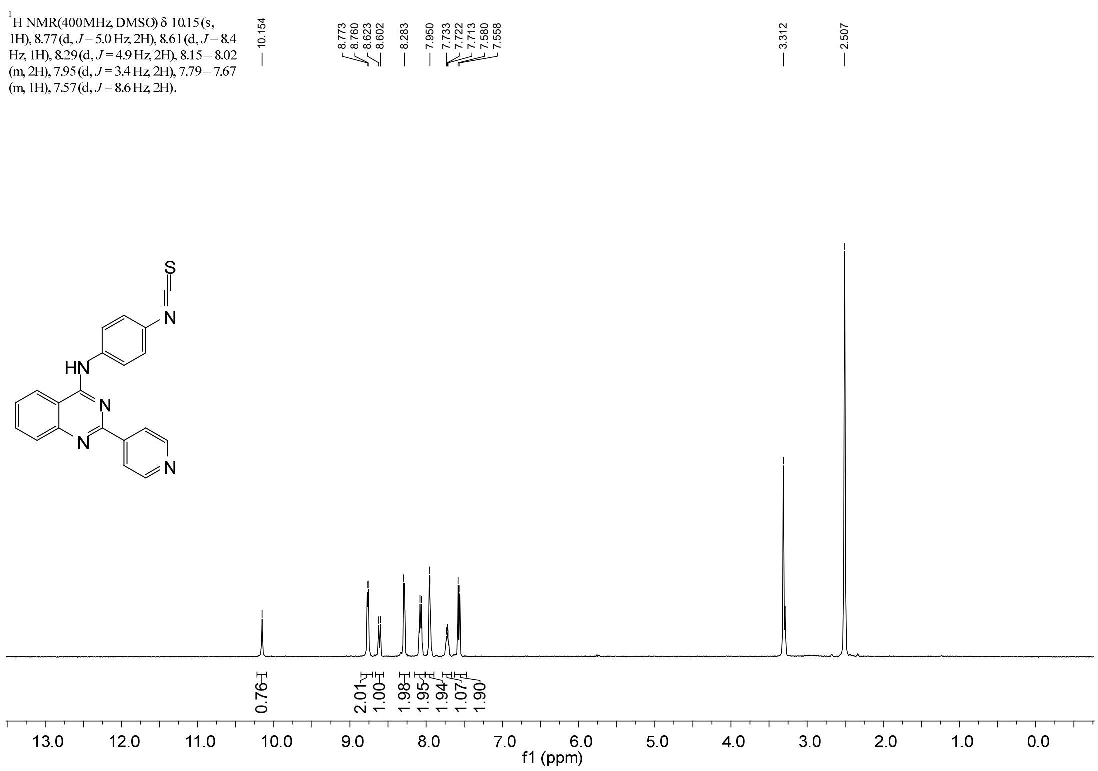

Figure S91. ^{1}H NMR (400 MHz, d_6-DMSO) of **30**

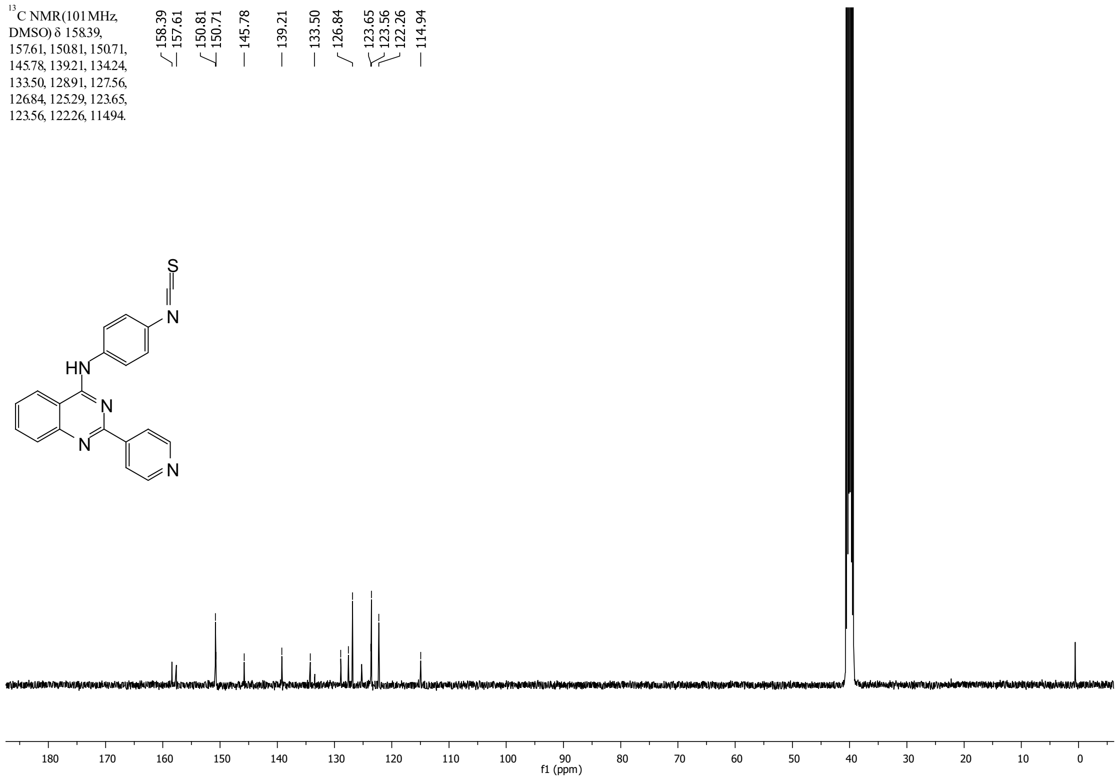

Figure S92. ^{13}C NMR (101 MHz, d_6-DMSO) of **30**

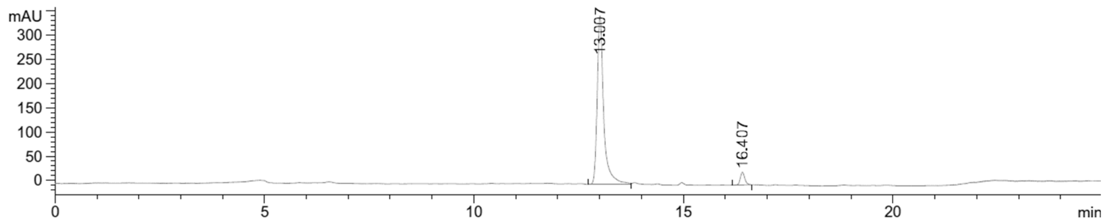

Figure S93. HPLC analysis of **31**

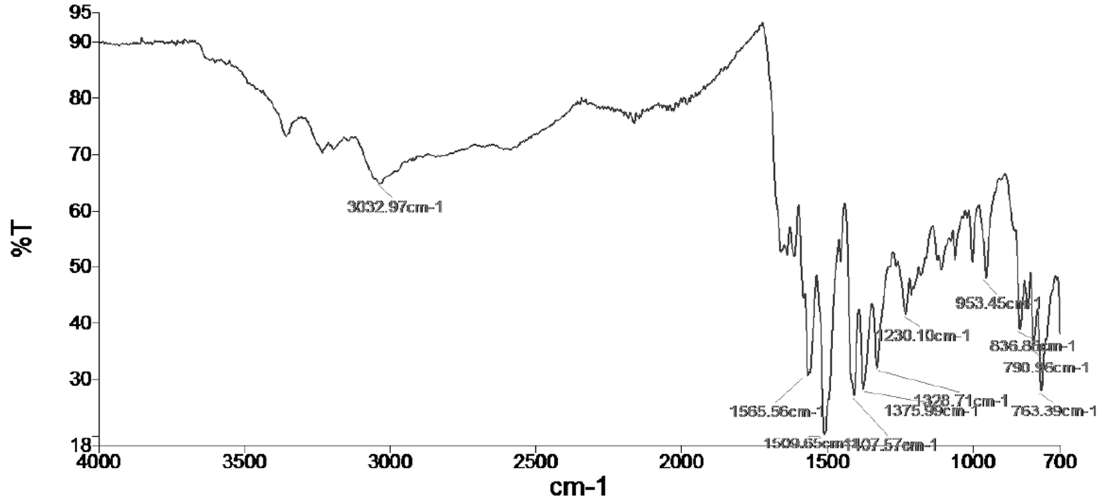

Figure S94. IR spectra of **31**

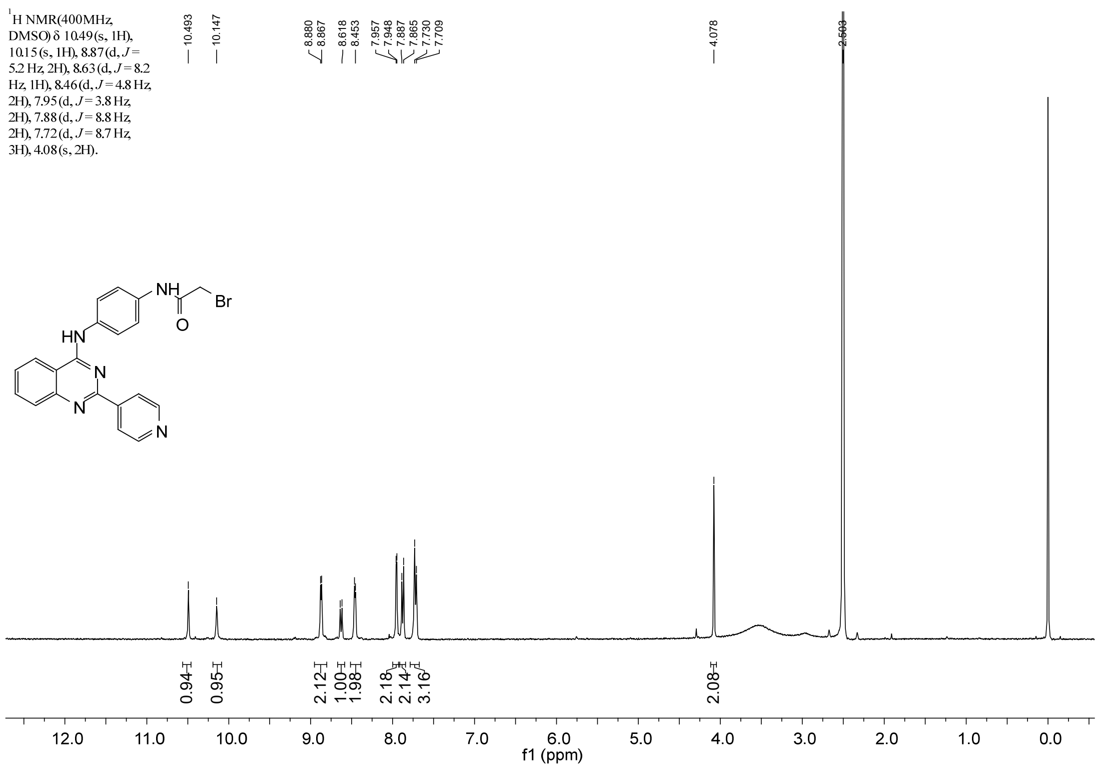

Figure S95. ^{1}H NMR (400 MHz, d_6-DMSO) of **31**

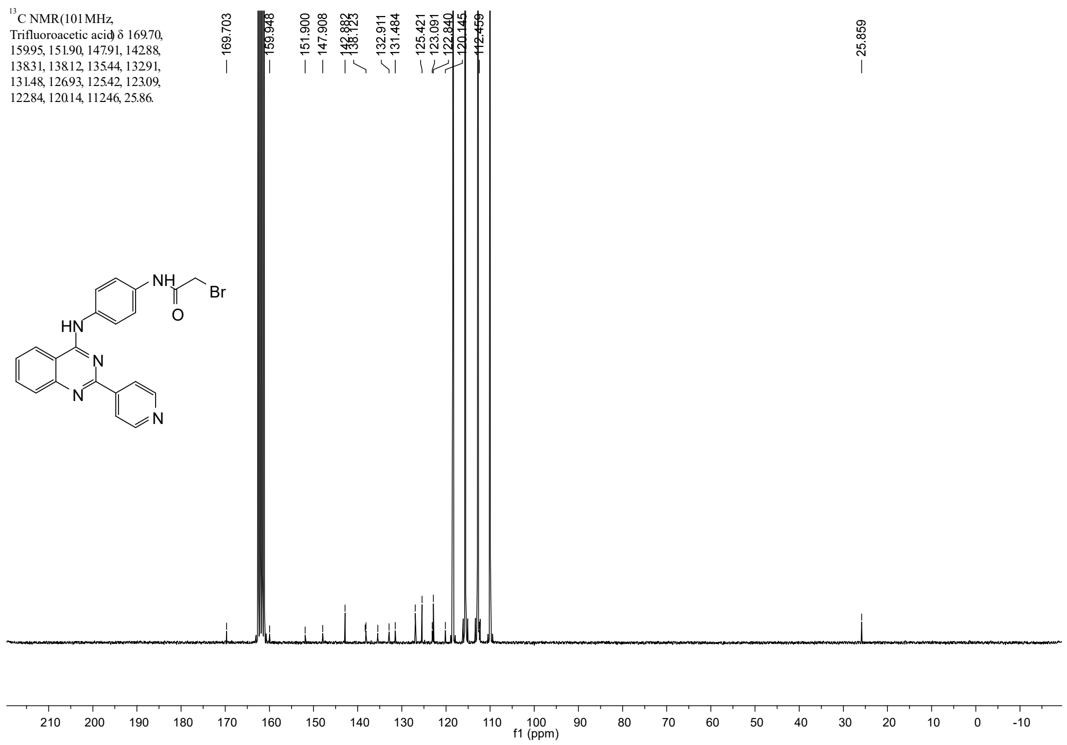

Figure S96. ^{13}C NMR (101 MHz, d_1-CF_3COOD) of **31**

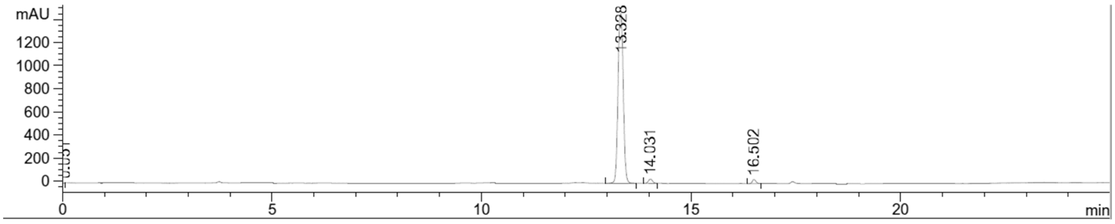

Figure S97. HPLC analysis of **32**

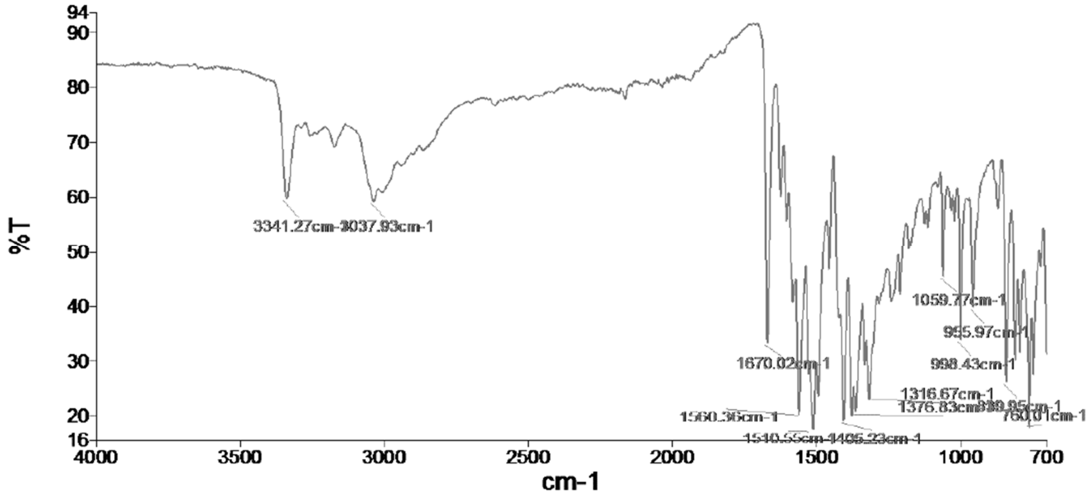

Figure S98. IR spectra of **32**

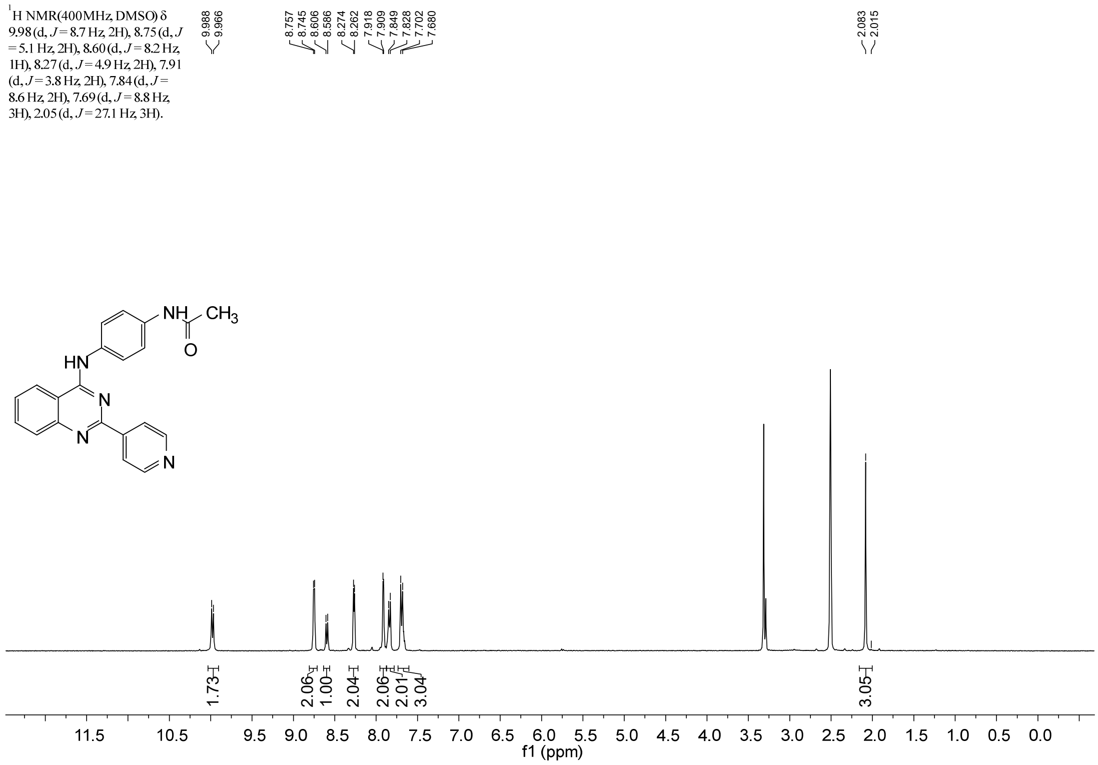

Figure S99. ^{1}H NMR (400 MHz, d_6-DMSO) of **32**

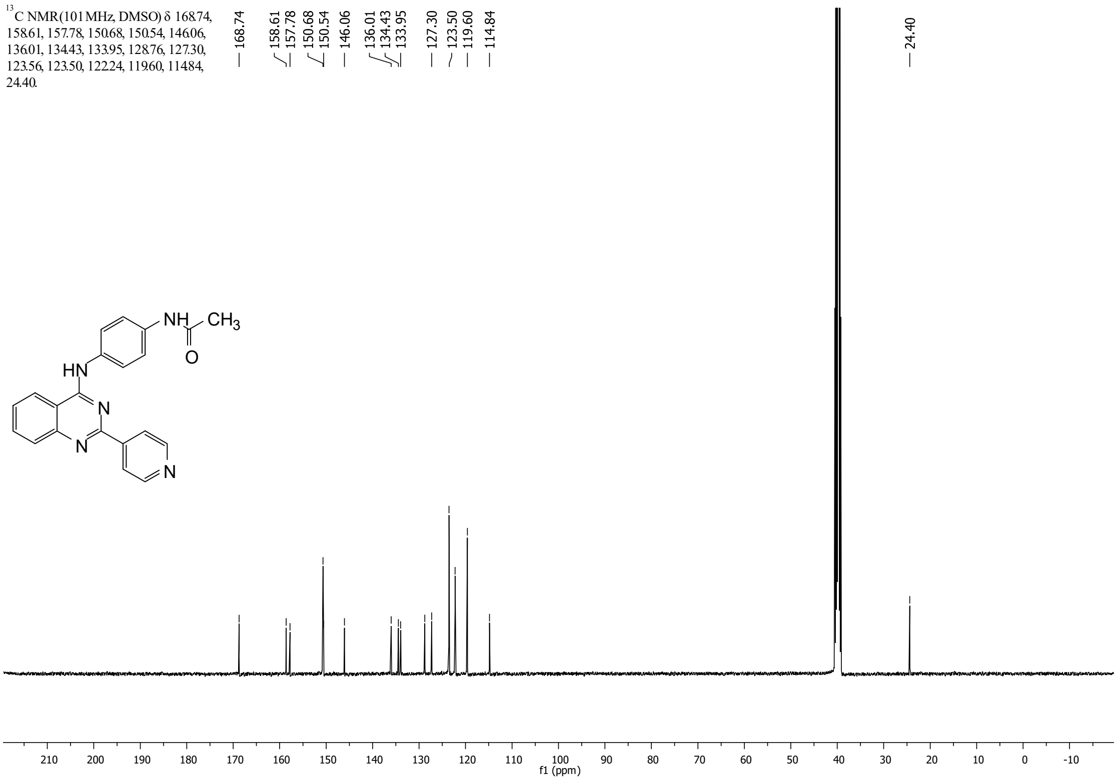

Figure S100. ^{13}C NMR (101 MHz, d_6-DMSO) of **32**

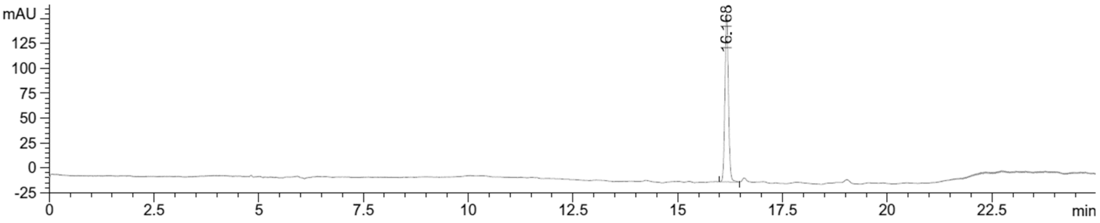

Figure S101. HPLC analysis of **33**

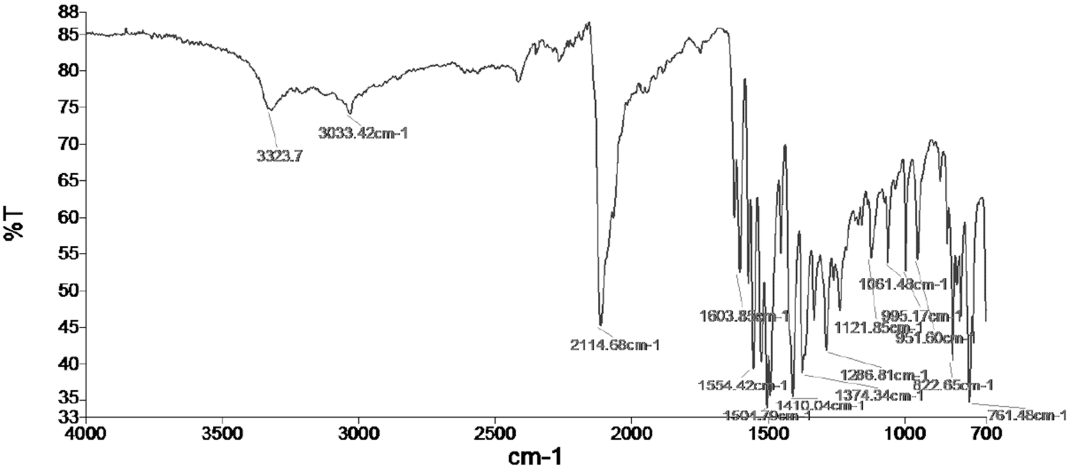

Figure S102. IR spectra of **33**

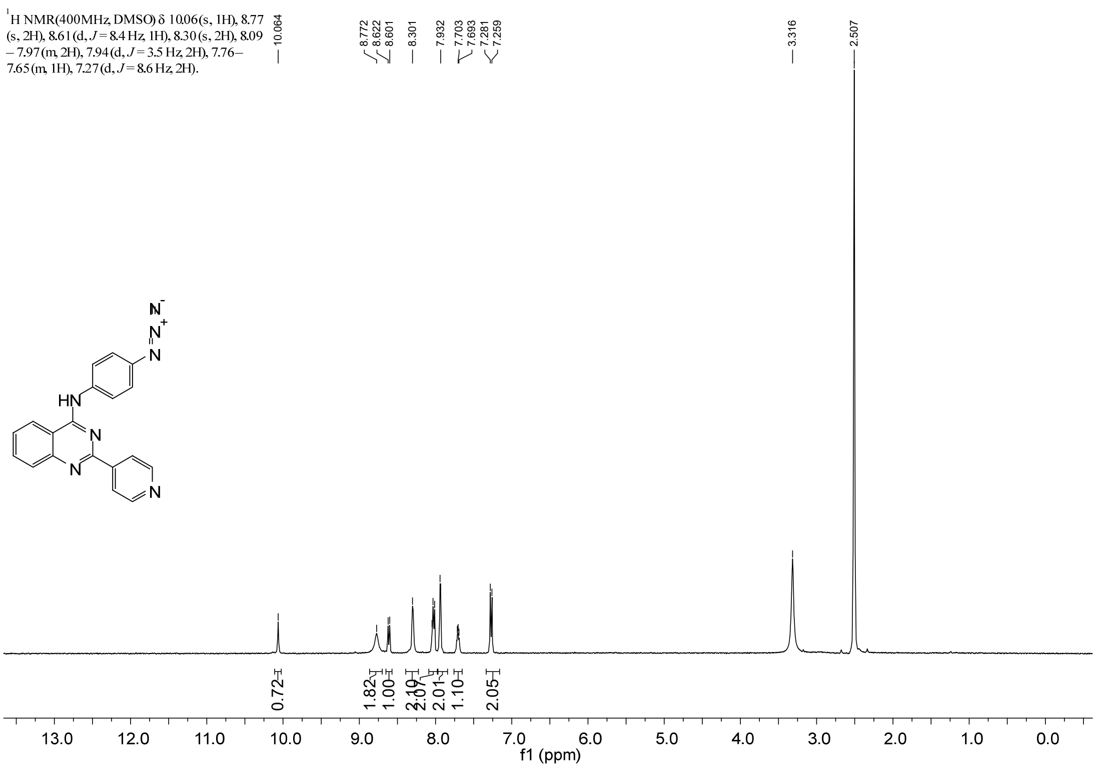

Figure S103. ^{1}H NMR (400 MHz, d_6-DMSO) of **33**

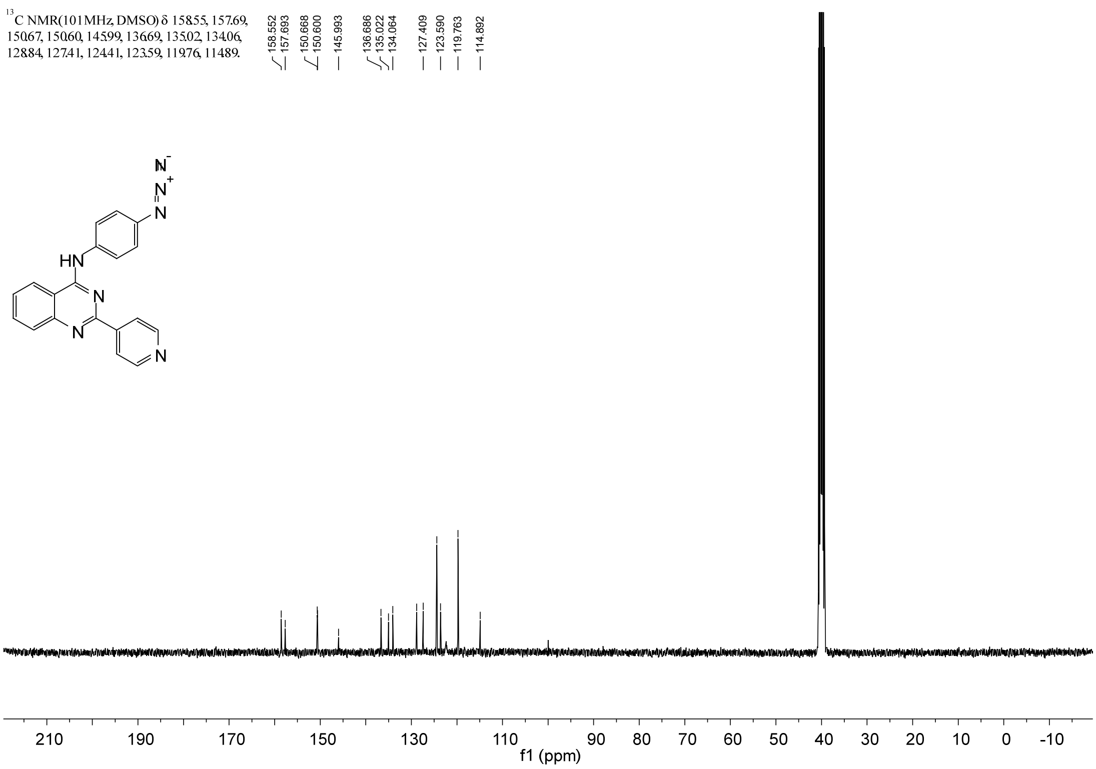

Figure S104. ^{13}C NMR (101 MHz, d_6-DMSO) of **33**
